# Solution and Solid State Studies of Urea Derivatives of DITIPIRAM Acting as Powerful Anion Receptors

**DOI:** 10.3390/molecules26061788

**Published:** 2021-03-22

**Authors:** Patryk Niedbała, Kajetan Dąbrowa, Agnieszka Cholewiak-Janusz, Janusz Jurczak

**Affiliations:** Institute of Organic Chemistry Polish Academy of Sciences, Kasprzaka 44/52, 01-224 Warsaw, Poland; pniedbala@icho.edu.pl (P.N.); kdabrowa@icho.edu.pl (K.D.); cholewiakagnieszka@gmail.com (A.C.-J.)

**Keywords:** anion recognition, acyclic receptors, supramolecular chemistry, DITIPIRAM

## Abstract

Herein, we present the synthesis and anion binding studies of a family of homologous molecular receptors **4**–**7** based on a DITIPIRAM (8-propyldithieno-[3,2-b:2′,3′-e]-pyridine-3,5-di-amine) platform decorated with various urea *para*-phenyl substituents (NO_2_, F, CF_3_, and Me). Solution, X-ray, and DFT studies reveal that the presented host–guest system offers a convergent array of four urea NH hydrogen bond donors to anions allowing the formation of remarkably stable complexes with carboxylates (acetate, benzoate) and chloride anions in solution, even in competitive solvent mixtures such as DMSO-*d*_6_/H_2_O 99.5/0.5 (*v*/*v*) and DMSO-*d*_3_/MeOH-*d*_3_ 9:1 (*v*/*v*). The most effective derivatives among the series turned out to be receptors **5** and **6** containing electron-withdrawing F- and -CF_3_ *para*-substituents, respectively.

## 1. Introduction

For over twenty years, many groups working in the field of supramolecular chemistry have made substantial efforts to restore the parity between the molecular chemistry of anions and cations. As a result, many monographic studies and literature reviews have been published [1,2,3,4,5,6,7,8,9,10,11,12,13,14,15,16]. Still, the design and synthesis of receptors capable of strong and selective binding of negatively charged molecules is an essential problem considering the great role that anions play in many biological and chemical processes. Moreover, binding of anions using receptors with precisely selected properties opens the way to many important applications in medicine, pharmacy, catalysis, or transport processes [17,18,19,20,21,22,23,24,25,26,27,28,29]. Despite the high demand for the above-mentioned hosts for anions, numerous studies, and the development of computational techniques, the prediction of the binding properties of artificial hosts is still not a trivial task, sometimes even impossible when the anionic guest is chiral. It is, therefore, necessary to perform their synthesis and then time-consuming and expensive measurements. 

Despite the shortage of basic research aimed at defining factors affecting both the efficiency and the selectivity of binding of anions, there are some useful rules that can be collected based on the previous studies. First of all, neutral anion receptors have generally an advantage over the positively charged systems due to their much higher selectivity, despite the lower stabilities of receptor–anion complexes. These result from the presence of binding groups such as hydrogen bond donors, which allow for the establishment of highly directional interactions with the anion [30,31,32,33,34,35,36]. Furthermore, the unquestionable advantage of acyclic receptors, compared to their cyclic analogues, is their relatively simpler synthesis due to the lack of a problematic and low-yielding macrocyclization step. Additionally, the structure of acyclic receptors is generally tunable, allowing facile post-functionalization (to increase the binding affinity or to modify selectivity) compared to macrocyclic architectures. Among other factors, this turned researchers’ attention to the acyclic systems in the molecular recognition of anions [37,38,39,40]. 

In designing non-macrocyclic receptors, one should take into consideration the correlation between the rigidity of the central unit (platform) and the efficiency of the anion binding process. Moreover, very often, small changes in the structure of the receptor’s binding site have a significant effect on the receptor’s anion binding properties. In addition, symmetry of the receptor is as crucial as the rest of the factors defining its structure. Receptors with C2 symmetry are very efficient when binding anions such as Y-shaped carboxylates, spherical halides, or tetrahedral phosphates. Simultaneous study of both the anion binding properties and structural modifications of the host–guest complexes allows for deeper understanding of the mechanisms controlling the anion recognition phenomenon, however, it is still necessary to apply the iterative synthetic approach due to the complex relationship between the structure and anion binding properties of the designed artificial systems. 

**Figure 1:**
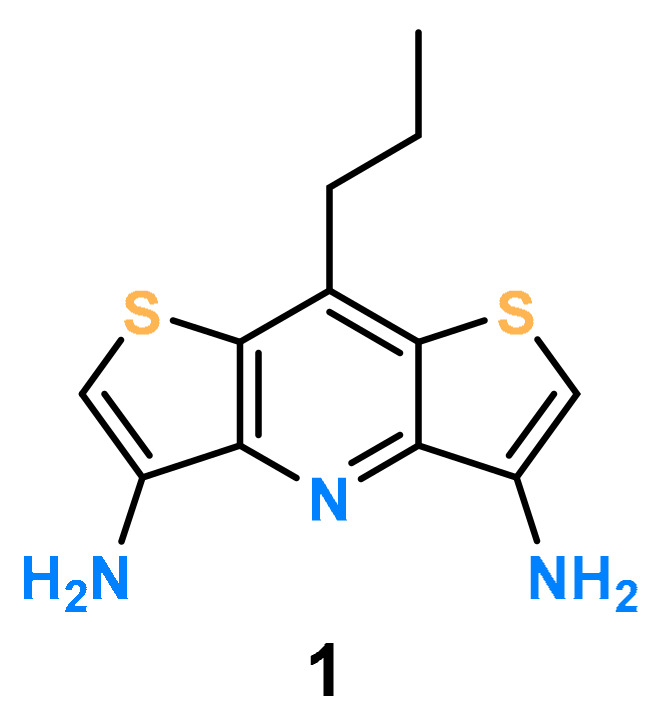
Structure of the 8-propyldithieno [3,2-b:2′,3′-e]pyridine-3,5-diamine (DITIPIRAM) platform.

Considering the above-mentioned conditions, 8-propyldithieno [3,2-b:2′,3′-e]pyridine-3,5-diamine (DITIPIRAM) (**1**) fits perfectly to the role of the platform for designing potent acyclic receptors for anions (Figure 1).

This unique building block was recently introduced by us into supramolecular chemistry [41]. Utilization of DITIPIRAM provides a highly preorganized geometry of anion binding sites for the design of acyclic receptors decorated with functionalized urea arms. Our results proved DITIPIRAM to be an excellent choice as a platform for putative anion receptors.

## 2. Results and Discussion

**Figure 2:**
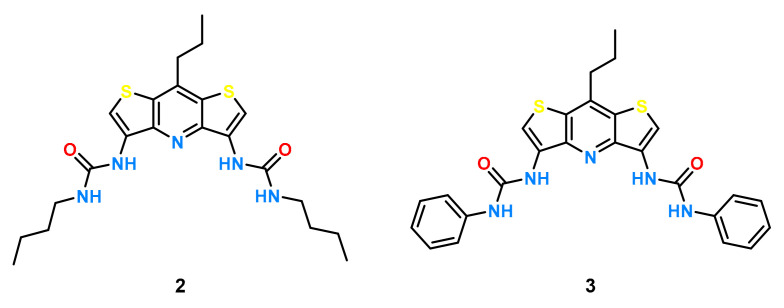
Structures of receptors **2** and **3**.

In our recent study [41] with model urea receptors incorporating the DITIPIRAM building block, we showed significantly higher efficiency in anion binding by the receptor with phenyl substituents **3** compared to that with analogue **2** decorated with butyl substituents (Figure 2).

In the present publication, we decided to extend our research on the anion recognition properties of molecular receptors built on the DITIPIRAM platform. Here, we investigated the impact of the aromatic substituents on the anion binding affinity and selectivity of novel analogs.

To analyze the influence of this change, it was necessary to limit our research to two variables, i.e., modification of the size and electron properties of the *para*-substituent relative to the urea group by using various groups with electron withdrawing (EWG) and electron donating (EDG) characters. Different substitution patterns could introduce, among other effects, steric hindrance, which might inhibit the binding of anions in the receptors’ gap, and thus the impact of substituents would be impossible to elucidate. 

**Scheme 1:**
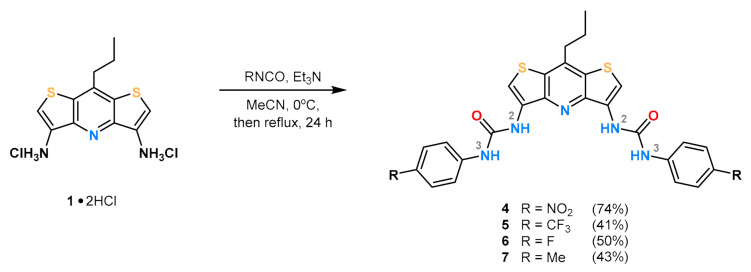
Synthesis of acyclic receptors **4**–**7** varying in *para*-substituent (R) on the aryl rings.

The designed receptors were prepared as described in Scheme 1. In each case, the synthesis of aromatic urea receptors containing the DITIPIRAM core included the use of 8-propyldithieno[3,2-b:2*′*,3*′*-e]pyridine-3,5-diamine dichydrochloride (**1** × 2HCl), triethylamine, and the corresponding isocyanate. For details see the Materials and Methods section.

Subsequently, we tested the binding properties of receptors **5**–**7** using ^1^H-NMR titration experiments, keeping the concentration of the receptor (~10^−2^ M) constant. Unfortunately, receptor **4** turned out to be insoluble in the tested solvent, preventing the determination of its binding properties. Thankfully, the rest of receptors readily dissolved in the studied solvent mixtures (DMSO-*d_6_*, DMSO-*d_6_*/H_2_O 99.5/0.5 *v*/*v* and DMSO-*d_6_*/CD_3_OH 9:1 *v*/*v*). The highest solubility was exhibited by receptors **5** and **6** equipped with trifluoromethyl and fluorine substituents, respectively. As model anionic guests, we selected chloride (Cl*^−^*) as the model spherical guest (p*K*_a,H2O_ < 1), acetate (MeCO_2_*^−^*, p*K*_a,H2O_ = 4.76) and benzoate (PhCO_2_*^−^*, p*K*_a,H2O_ = 4.20) as Y-shaped carboxylates with a geometry match to the urea group, and dihydrogen phosphate (H_2_PO_4_*^−^*, p*K*_a,H2O_ = 2.14), as both a donor and acceptor of hydrogen bonds. This set of anions is routinely employed in anion recognition studies. All anions were added as corresponding salts with a bulky and diffused tetrabutylammonium (TBA) cation to ensure complete dissolution of salt and to limit the eventual impact of cations on the molecular recognition process by synthetized receptors. In general, the addition of anion aliquots caused substantial shifts of the signal of urea NH and aryl CH protons and the binding isotherms were fitted using a 1:1 (host/guest) binding model. However, in the case of the H_2_PO_4_^−^ guest, the binding isotherm could not be fitted to either 1:1 or 2:1 anion/receptor binding models, suggesting proton transfer processes such as deprotonation of the bound anionic species by aliquots of free H_2_PO_4_^−^ [42]. Despite the fact that it was not possible to fit the data from ^1^H-NMR titrations with dihydrogenphosphate to any tested models, the experiments clearly indicated the strong binding of H_2_PO_4_^−^ with receptors **5**–**7**.

The results from the performed titrations are collected in Table 1 along with the data for parent receptor ***3*** for comparison [43].

The data presented in Table 1 demonstrate that receptors tend to bind tested anions with high affinity and show the expected selectivity for MeCO_2_^−^ over PhCO_2_^−^ and Cl^−^. The stability constants for complexes **3** and **5**–**7** with carboxylates in a highly competitive DMSO-*d*_6_ + 0.5% H_2_O (*v*/*v*) solvent mixture were too high to be determined accurately using the ^1^H-NMR titration technique (K > 10,000 M^−1^), and an even more competitive solvent mixture (DMSO-*d*_6_ + 10% CD_3_OH *v*/*v*) was employed to determine their binding properties. In these solvents mixtures the receptors have to compete with water and methanol (added in large excess to the receptor) for anion binding. In addition, DMSO is a strong hydrogen bond acceptor, able to interact strongly with receptor urea NH protons (as well as water and methanol solvents).

**Figure 3:**
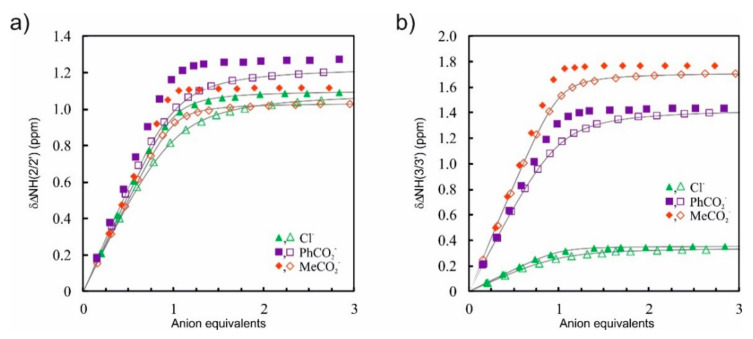
Representative comparison of chemical shift changes (Δδ) for urea protons NH(2/2′) (**a**) and NH(3/3′) (**b**) upon addition of TBA^+^X^–^ (Cl^−^, PhCO_2_^−^, MeCO_2_^−^) to the solution of **6** in DMSO-*d*_6_ + 0.5% H_2_O (closed symbols) and DMSO-*d*_6_ + 10% CD_3_OH (open symbols); fitted binding isotherms (gray lines).

Interestingly, the differences between binding constants of MeCO_2_^−^ vs. PhCO_2_^−^ (*K*_MeCO2−_/*K*_PhCO2−_ = 2.6–3.1) and in particular PhCO_2_^−^ vs. Cl^−^ (*K*_PhCO2−_/*K*_Cl−_ = 1.6–2.3) in this solvent mixture were not as high as expected when comparing only the relative anion basicity (MeCO_2_^−^ > PhCO_2_^−^ >> Cl^−^). This suggests that a preorganized binding pocket of the DITIPIRAM platform ensures the formation of strong complexes with anions with different sizes, geometries, and basicity, and this is clearly manifested by the marginal changes, in particular for carboxylates, in the chemical shifts of urea and aryl protons after the addition of one equivalent of anionic salts (Figure 3).

Upon addition of TBACl to the solution of receptors **3** and **5**–**7**, the resonances of urea NH protons 3/3′ experienced a much smaller downfield shift compared with NH protons 2/2′ (Δδ_NH3-NH2_ = 0.74–0.84 ppm). This behavior reveals that the chloride anion, which is the smallest anion among the series, interacts more strongly with urea NH protons 2/2′ than with protons 3/3′, whereas for larger carboxylates this difference is much less noticeable.

In addition, receptor **7** that incorporates methyl substituents shows the lowest binding affinity among the series. This might be rationalized by the decreased acidity of urea NH protons resulting from the transfer of electron density to the adjacent aromatic ring by the methyl group. Association constants for this receptor are even lower than for receptor **3,** which bears phenyl groups. Receptors **5** and **6,** equipped with electron-withdrawing *para*-substituents (-CF_3_ and -F groups, respectively), exhibit, as expected, higher affinity toward anions than that of both receptors **3** and **7**. Interestingly, however, anion binding properties of **5** and **6** are similar within the experimental error. On the basis of a simple comparison of the corresponding Hammet constants (σ_para_ = 0.54 and 0.06 for the -CF_3_ and -F groups, respectively), one can assume that receptor **5** should exhibit higher affinity to anions due to the stronger electron-withdrawing effect of the trifluoromethyl group.

These experimental results demonstrate that the prediction of the exact binding properties of even simple and rigid systems, as studied here for receptors **5** and **6**, is still very challenging since many subtle factors have an impact on the overall binding free energy of anion–receptor complexes. It is possible the more bulky CF_3_ group interacts more strongly with the solvent molecules, especially with water and methanol, than with the fluorine atom, thus disturbing the solvation shell around the receptor and/or the bound anion. Nevertheless, the comparison of titration data for **5** vs. **6** indicates that receptor **5** produces a slightly stronger complex with the benzoate anion, possibly due to the stronger π-π interaction between *p*-C_6_H_4_CF_3_ aryls and the phenyl of benzoate.

**Figure 4:**
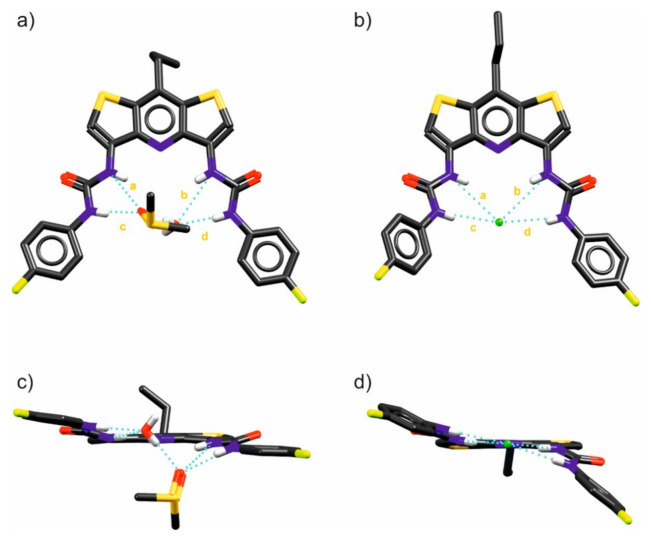
X-ray structures of **6**@(DMSO∙H_2_O) solvate (**a**,**c**) and the **6**@(Cl)TBA anionic complex (**b**,**d**). Non-acidic protons and disorder were omitted for clarity; the hydrogen bonds are shown as dashed blue lines. For hydrogen bond lengths (**a**–**d**), see the inset table in Figure 5.

To further evaluate the binding properties of DITIPIRAM-based receptors, we studied the structures of the DMSO-H_2_O solvate of receptor **6** and its complex with chloride (added as a TBA salt). The crystals suitable for X-ray diffraction studies were grown by a slow diffusion of water into DMSO/H_2_O solutions of **6** and **6** with an excess of TBACl. Analyzing the X-ray structure of receptor **6** solvate reveals that the binding pocket of **6** is too large to accommodate H_2_O or DMSO molecules alone, resulting in binding of the DMSO-H_2_O dimer. The DMSO is located above the binding pocket, interacting with one urea arm and with a water molecule via a hydrogen bond, whereas the water molecule is localized inside the binding pocket, also forming three hydrogen bonds: two with the second urea arm of the receptor and one with a DMSO. It is notable that the receptor is preorganized to bind anions due to the favorable *syn-syn* conformation of the urea arms (Figure 4a,c).

The X-ray structure of the chloride complex with receptor **6** reveals that the receptor interacts strongly with this anionic guest by means of all four available urea NH hydrogen bonds. The chloride anion is located at the center of the binding site, confirming the great complementarity of the receptors’ binding pocket to anions. The urea arms are in *syn-syn* conformation, which enables a comparative binding of the two urea groups (Figure 4b,d).

**Figure 5:**
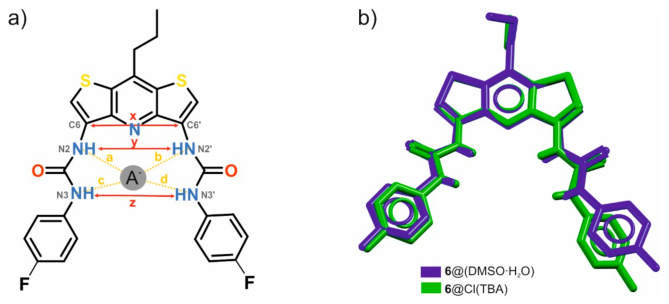
Geometrical descriptors of the binding pocket of the DITIPIRAM-based receptor **6** (**a**), superposition of crystal structures of **6**∙(DMSO∙H_2_O) solvate (colored in blue) and **6**∙TBACl (colored in green) (**b**).

Comparing the individual lengths of the hydrogen bonds and the distances shown in Figure 5 and Table 2, it may be noted that despite the very similar geometry and size of the binding pocket (compare the distances marked with the letters y and z in the mentioned figure), in the case of complexation of the water and DMSO molecules, the urea arms of receptor **6** expand to a greater extent than they do in chloride anion bonding. The distances between the urea nitrogen atoms adjacent to the *p*-fluorophenyl substituents differ for both complexes by as much as 0.58 Å despite the evident similarity between these structures (Figure 5b).

Comparing the lengths of the NH∙∙∙Cl^−^ hydrogen bonds (a–b vs. c–d) in the crystal structure of **6**∙TBACl (Figure 5, Table 2) with the shift changes of urea NH protons (2/2′ vs. 3/3′) upon addition of TBACl to a solution of receptor (Figure 3) it could be noticed that host **6**, binding the chloride anion, behaves differently in solution than in a solid state. Specifically, in solution, the NH protons (2/2′) are involved in stronger interactions with the anion as compared to with the protons (3/3′), whereas the opposite situation occurs in the solid state, i.e., the lengths of the hydrogen bonds a–b are longer than with c–d, indicating a weaker interaction. This seemingly contradictory result could be attributed to the crystal packing forces that do not allow the chloride anion to enter too deeply inside the binding pocket [43].

**Figure 6:**
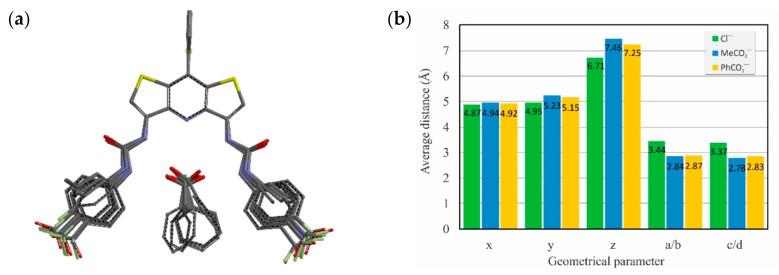
Superposition of DFT-calculated structures of receptors **2−7** and their complexes with chloride, acetate, and benzoate exemplifies the high preorganization of the DITIPIRAM moiety (**a**) and average values of geometrical parameters describing anion complexes (**b**); see Figure 5a for labels.

To achieve better insights into the binding mode of receptors **2**–**7** toward anions in solution, we performed DFT calculations using M06-2X combined with a 6-31G(d) basis set and C-PCM model (DMSO, ε = 46.83) to approximate the solvent effects. The superposition of energy-minimized conformations of receptors and their complexes with Cl^−^, MeCO_2_^−^, and PhCO_2_^−^ along with the average geometrical parameters and lengths of hydrogen bond interactions in the complexes are demonstrated in Figure 6.

The results of DFT calculations are in line with the experimental data from the solution and solid state, showing that molecular receptors based on the DITIPIRAM platform provide well-preorganized binding sites for anion recognition. In addition, only minimal structural rearrangement of the receptor conformation is necessary prior to anion binding.

## 3. Materials and Methods

### 3.1. Reagents and General Methods

All reagents were used as received. The solvents were dried by distillation over the appropriate drying agents. All solvents were obtained from common suppliers and used as received. TLC was carried out on Merck Kieselgel F254 plates (Merck, Germany). The NMR spectra were recorded on a Bruker Mercury 400 instrument (Bruker, Ettlingen, Germany). Chemical shifts are reported in ppm (δ) and were set to the solvent residue peak. *J* coupling constants values are reported in Hz. Mass spectral analyses were performed with the ESI-TOF technique on a Mariner mass spectrometer from PerSeptive Biosystem (Waltham, MA, USA). The lowest energy conformations of the complexes of receptor **3**–**7** with chloride, acetate, and benzoate were found after conducting a conformational search analysis. The selected conformers with the lowest energies were then optimized without any constrains at the DFT/M06-2X/6-31G(d)/C-PCM:DMSO level of theory using program Spartan’18 Parallel Suite (see Supplementary Information for details) [44,45,46,47,48].

### 3.2. Synthetic Procedures

#### 3.2.1. Synthesis of 1-(4-nitrophenyl)-3-(12-{[(4-nitrophenyl)carbamoyl]amino}-8-propyl-6,10-dithia-2-azatricyclo[7.3.0.0^3^,⁷]dodeca-1,3(7),4,8,11-pentaen-4-yl)urea (4)

To a suspension of **1**∙2HCl (0.60 g, 1.78 mmol) in MeCN (100 mL) were added dropwise Et_3_N (0.75 mL, 5.35 mmol) and *p-*nitrophenyl isocyanate (0.88 mL, 5.35 mmol) at 0 °C. The mixture was stirred for 8 h at rt and the solvent was evaporated off, giving the crude product that was dissolved in DMSO and precipitated with water, filtered off, washed with water (~200 mL), and dried under vacuum to yield product **4** (0.78 g, 1.32 mmol, 74%) in the form of a yellow solid (mp 257–258 °C, decomposition).

^1^H-NMR (400 MHz, DMSO-*d_6_*) δ 10.33 (s, 2H), 9.16 (s, 2H), 8.25 (d, *J* = 9.2 Hz, 4H), 8.14 (s, 2H), 7.79 (d, *J* = 9.2 Hz, 4H), 3.14 (t, *J* = 7.5 Hz, 2H), 2.0–1.81 (m, 2H), 1.00 (t, *J* = 7.3 Hz, 3H). ^13^C{^1^H} NMR (100 MHz, DMSO-*d_6_*) δ 151.8, 146.6, 146.1, 141.2, 140.3, 129.4, 128.1, 125.3, 117.4, 111.8, 34.6, 20.9, 14.0. HRMS ESI *(m/z)* calc. for C_26_H_21_N_7_O_6_S_2_Na [M+Na]^+^: 614.0892, found: 614.0897. Anal (%) calc. for C_26_H_21_N_7_O_6_S_2_·DMSO: C 50.21, H 4.06, N 14.64, found: C 50.12, H 4.23, N 14.87. IR (KBr) 3345, 3083, 2960, 1714, 1551, 1496, 1412, 1328, 1212, 1109, 1007, 949, 850, 749, 705, 610 527 cm^−1^.

#### 3.2.2. Synthesis of 3-[8-propyl-12-({[4-(trifluoromethyl)phenyl]carbamoyl}amino)-6,10-dithia-2-azatricyclo[7.3.0.0^3^,^7^]dodeca-1,3(7),4,8,11-pentaen-4-yl]-1-[4-(trifluoromethyl)phenyl]urea (5)

To a suspension of **1**∙2HCl (0.60 g, 1.78 mmol) in MeCN (100 mL) were added dropwise Et_3_N (0.75 mL, 5.35 mmol) and *p-*(trifluoromethyl)phenyl isocyanate (0.75 mL, 5.35 mmol) at 0 °C. The mixture was stirred for 12 h in rt and the solvent was evaporated, giving the crude product that was recrystallized from EA and yielded after drying under vacuum product **5** (0.47 g, 0.74 mmol, 41%) in the form of a colorless solid (mp 287–288 °C, decomposition).

^1^H-NMR (400 MHz, DMSO-*d_6_*) δ 10.02 (s, 2H), 9.06 (s, 2H), 8.11 (s, 2H), 7.73 (dd, *J* = 29.8, 8.7 Hz, 8H), 3.13 (t, *J* = 7.5 Hz, 2H), 2.02–1.81 (m, 2H), 1.00 (t, *J* = 7.3 Hz, 3H). ^13^C{^1^H} NMR (100 MHz, DMSO-*d_6_*) δ 152.1, 146.6, 143.3, 140.3, 129.7, 128.1, 126.24 (d, *J* = 3.2 Hz), 124.5 (**C**F_3_, q, *J* = 271.1 Hz), 122.0 (q, *J* = 31.5 Hz), 117.7, 110.6, 34.6, 20.9, 14.00. HRMS ESI *(m/z)* calc. for C_28_H_21_F_6_N_5_O_2_S_2_Na [M+Na]^+^: 660.0939, found: 660.0931. Anal (%) calc. for C_28_H_21_F_6_N_5_O_2_S_2_∙H_2_O: C 51.29, H 3.54, N 10.68, found: C 51.13, H 3.45, N 10.66. IR (KBr) 3653, 3302, 3089, 2962, 1906, 1711, 1674, 1607, 1563, 1411, 1362, 1320, 1166, 1114, 863, 689, 507 cm^−1^.

#### 3.2.3. Synthesis of 1-(4-fluorophenyl)-3-(12-{[(4-fluorophenyl)carbamoyl]amino}-8-propyl-6,10-dithia-2-azatricyclo[7.3.0.0^3^,^7^]dodeca-1,3(7),4,8,11-pentaen-4-yl)urea (6)

To a suspension of **1**∙2HCl (0.60 g, 1.78 mmol) in MeCN (100 mL) were added dropwise Et_3_N (0.75 mL, 5.35 mmol) and *p-*fluorophenyl isocyanate (0.61 mL, 5.35 mmol) at 0 °C. The mixture was stirred for 12 h in rt and the solvent was evaporated, giving a crude product which was dissolved in EA (~30 mL) and washed with water (3 × 30 mL). The organic phase was dried over anhydrous Na_2_SO_4_, the solvent was evaporated, and the remaining residue was dried under vacuum, yielding product **6** (0.48 g, 0.89 mmol, 50%) in the form of a colorless solid (mp 269 °C, decomposition).

^1^H-NMR (400 MHz, DMSO-*d_6_*) 9.64 (s, 2H), 8.93 (s, 2H), 8.04 (s, 2H), 7.80–7.40 (m, 4H), 7.17 (t, *J* = 8.9 Hz, 4H), 3.11 (t, *J* = 7.5 Hz, 2H), 2.01–1.80 (m, 2H), 0.99 (t, *J* = 7.3 Hz, 3H). ^13^C{^1^H} NMR (100 MHz, DMSO-*d_6_*) 158.6, 156.2, 152.4, 146.6, 140.1, 136.0, 130.0, 128.0, 119.7 (x2), 115.5, 115.3, 109.8, 34.6, 20.9, 14.0. HRMS ESI *(m/z)* calc. for C_26_H_21_F_2_N_5_O_2_S_2_Na [M+Na]^+^: 560.1002, found: 560.1000. Anal (%) calc. for C_26_H_21_F_2_N_5_O_2_S_2_: C 58.09, H 3.94, N 13.03, found: C 57.47, H 3.96, N 12.59. IR (KBr) 3327, 3097, 2958, 2932, 2871, 1867, 1697, 1655, 1615, 1569, 1532, 1506, 1365, 1216, 1099, 828, 786, 659, 514, 486 cm^−1^.

#### 3.2.4. Synthesis of 1-(4-methylphenyl)-3-(12-{[(4-methylphenyl)carbamoyl]amino}-8-propyl-6,10-dithia-2-azatricyclo[7.3.0.0^3^,^7^]dodeca-1,3(7),4,8,11-pentaen-4-yl)urea (7)

To a suspension of **1**∙2HCl (0.60 g, 1.78 mmol) in MeCN (100 mL) were added dropwise Et_3_N (0.75 mL, 5.35 mmol) and *p-*methylphenyl isocyanate (0.67 mL, 5.35 mmol) at 0 °C. The mixture was stirred for 24 h at rt and the solvent was evaporated off giving a crude product that was recrystallized from the mixture of EA and acetone. Then, the solid was filtered off and dried under vacuum yielding product **7** (0.41 g, 0.77 mmol, 43%) in the form of a colorless solid (mp 256–257 °C (decomposition).

^1^H-NMR (400 MHz, DMSO-d_6_) 9.48 (s, 2H), 8.91 (s, 2H), 8.03 (s, 2H), 7.43 (d, *J* = 8.2 Hz, 4H), 7.13 (d, *J* = 8.1 Hz, 4H), 3.12 (t, *J* = 7.5 Hz, 2H), 2.27 (s, 6H), 2.05 – 1.74 (m, *J* = 14.8, 7.3 Hz, 2H), 0.99 (t, *J* = 7.3 Hz, 3H). ^13^C{^1^H} NMR (100 MHz, DMSO-d_6_) 152.3, 146.6, 140.1, 137.0, 130.8, 130.2, 129.3, 128.0, 118.1, 109.5, 34.6, 20.9, 20.3, 14.0. HRMS ESI (*m*/*z*) calc. for C_28_H_27_N_5_O_2_S_2_Na [M+Na]^+^: 552.1504, found: 552.1507. Anal (%) calc. for C_28_H_27_N_5_O_2_S_2_∙0.5H_2_O: C 62.43, H 5.24, N 13.00, found: C 62.29, H 4.80, N 12.88. IR (KBr) 3299, 3107, 2958, 2919, 2869, 1712, 1603, 1560, 1514, 1359, 1310, 1202, 1173 1045, 815, 647, 508 cm*^−^*^1^.

## 4. Conclusions

The obtained receptors **4**–**7,** containing a DITIPIRAM core decorated with urea-aryl tethers, constitute a class of extremely effective anion receptors. Solution studies demonstrate very high affinity of **5**–**7** to carboxylates (acetate, benzoate) and even much less basic chloride in demanding solvent mixtures (DMSO-*d*_6_/H_2_O 99.5:0.5 *v/v* and DMSO-*d*_6_/CD_3_OH 9:1 *v*/*v*). The most effective derivatives among the series turned out to be those containing electron withdrawing *para*-substituents: trifluoromethyl (**5**) and fluoride (**6**). Solution, X-ray, and DFT studies reveal that the binding pocket of the receptors is highly preorganized, allowing cooperative binding of anions by four hydrogen bond donor groups.

## Figures and Tables

**Table 1:** Stability constants K_a_ (M^−1^) for 1:1 (host/guest) complexes of receptors **3** and **5**–**7** with anions ^a^.

Receptor	R^b^	Solvent System	Cl^−^	MeCO_2_^−^	PhCO_2_^−^
**3**	H	DMSO-*d_6_* + 0.5% H_2_O	3350 ± 60	>10,000 ^c^	>10,000 ^c^
		DMSO-*d_6_* + 10% CD_3_OH	950 ± 10	4200 ± 90	1560 ± 20
**5**	CF_3_	DMSO-*d_6_* + 0.5% H_2_O	3400 ± 80	>10,000 ^c^	>10,000 ^c^
		DMSO-*d_6_* + 10% CD_3_OH	1040 ± 20	6300 ± 300	2400 ± 80
**6**	F	DMSO-*d_6_* + 0.5% H_2_O	4650 ± 250	>10,000 ^c^	>10,000 ^c^
		DMSO-*d_6_* + 10% CD_3_OH	1250 ± 20	6400 ± 300	2050 ± 40
**7**	Me	DMSO-*d_6_* + 0.5% H_2_O	2500 ± 50	>10,000 ^c^	n.d. ^d^
		DMSO-*d_6_* + 10% CD_3_OH	915 ± 10	2950 ± 150	n.d. ^d^

^a^ Determined using ^1^H-NMR titration at 298 K and HypNMR 2008 software for nonlinear fitting of the binding data [42]; anions added as tetrabutylammonium (TBA) salts; estimated errors <10%; each titration was performed at least twice. ^b^ *Para*-substituent on the aryl moiety, see Scheme 1. ^c^ Stability constants above the limit of the ^1^H-NMR titration technique (≥10,000). ^d^ Not determined due to precipitation of the complex.

**Table 2:** Exact distances x, y, z and a–d describing the discussed structures.

Scheme	Geometrical Descriptors (Å)			Hydrogen Bond Lengths (Å)			
	x	y	z	a	b	c	d
**6**∙(DMSO∙H_2_O)	4.89	5.11	7.12	3.18	3.19	2.84	2.83
**6**∙TBACl ^a^	4.82	4.87	6.54	3.39	3.46	3.29	3.30
Difference solvate—complex (Å)	0.07	0.24	0.58	−0.21	−0.27	−0.45	−0.47

^a^ Two nonequivalent structures of complexes exist in the crystal cell; the parameters for conformer #1 are given, see SI for details.

## Data Availability

Not applicable.

## Supplementary Materials

The following are available online at https://www.mdpi.com/1420-3049/26/6/1788/s1. File 1: Copies of ^1^H- and ^13^C-NMR spectra of all the compounds and relevant geometrical descriptors, hydrogen bond lengths, and calculated Gibbs free energies of energy-optimized complexes of receptors **2**–**7** with anions, X-ray crystallographic data, and titration experiments for receptors **5**–**7**. File 2 and File 3: checkCIF files for compound **6**@(DMSO∙H2O) and **6**@(Cl)TBA. CCDC 2063422 and 2063423 contains the supplementary crystallographic data for this paper. These data can be obtained free of charge via www.ccdc.cam.ac.uk/data_request/cif, or by emailing data_request@ccdc.cam.ac.uk, or by contacting The Cambridge Crystallographic Data Centre, 12 Union Road, Cambridge CB2 1EZ, UK; fax: + 44 1223 336033.

## Sample Availability

Samples of the compounds are available in limited quantity from the authors.
