# Supplementary material for: Solution and Solid State Studies of Urea Derivatives of DITIPIRAM Acting as Powerful Anion Receptors
Source: Molecules. 2021 Mar 22;26(6):1788. doi: 10.3390/molecules26061788 (PMC8004752; doi:10.3390/molecules26061788)

checkCIF/PLATON report

You have not supplied any structure factors. As a result the full set of tests cannot be run.

THIS REPORT IS FOR GUIDANCE ONLY. IF USED AS PART OF A REVIEW PROCEDURE FOR PUBLICATION, IT SHOULD NOT REPLACE THE EXPERTISE OF AN EXPERIENCED CRYSTALLOGRAPHIC REFEREE.

No syntax errors found. CIF dictionary Interpreting this report

Datablock: 5B_TBACl

```
Bond precision:  C-C = 0.0053 A          Wavelength=0.71073

Cell:           a=8.4288(4)    b=28.6479(13)        c=17.9789(8)
                alpha=90       beta=99.7682(13)     gamma=90
Temperature:    100 K

                Calculated                  Reported
Volume          4278.4(3)                   4278.4(3)
Space group     P 21                        P 1 21 1
Hall group      P 2yb                       P 2yb
                2(C26 H21 F2 N5 O2 S2),
Moiety formula  C14.93 H33.32 N, C16 H36    ?
                N, 0.537(C2 H5
Sum formula     C84 H114 Cl2 F4 N12 O4 S4   C42 H57 Cl F2 N6 O2 S2
Mr              1631.01                     815.50
Dx,g cm-3       1.266                       1.266
Z               2                           4
Mu (mm-1)       0.238                       0.238
F000            1736.0                      1736.0
F000'           1738.23
h,k,lmax        10,34,21                    10,34,21
Nref            15144[ 7738]                15117
Tmin,Tmax       0.932,0.992                 0.920,0.990
Tmin'           0.932

Correction method= # Reported T Limits: Tmin=0.920 Tmax=0.990
AbsCorr = MULTI-SCAN

Data completeness= 1.95/1.00      Theta(max)= 25.050

R(reflections)= 0.0365( 13305)    wR2(reflections)= 0.0807( 15117)

S = 1.028                   Npar= 1098
```

The following ALERTS were generated. Each ALERT has the format
test-name_ALERT_alert-type_alert-level.
Click on the hyperlinks for more details of the test.

Alert level C

```
STRVA01_ALERT_4_C           Flack test results are ambiguous.
           From the CIF: _refine_ls_abs_structure_Flack    0.350
           From the CIF: _refine_ls_abs_structure_Flack_su    0.050
PLAT089_ALERT_3_C Poor Data / Parameter Ratio (Zmax < 18) ........       7.05 Note
PLAT220_ALERT_2_C Non-Solvent Resd 1   C   Ueq(max)/Ueq(min) Range        3.8 Ratio
PLAT222_ALERT_3_C Non-Solvent Resd 1   H Uiso(max)/Uiso(min) Range        5.3 Ratio
PLAT340_ALERT_3_C Low Bond Precision on  C-C Bonds ...............    0.00534 Ang.
PLAT352_ALERT_3_C Short   N-H (X0.87,N1.01A)  N2B    -   H2NB   ..       0.74 Ang.
PLAT431_ALERT_2_C Short Inter HL..A  Contact Cl1A   ..  S2B     ..       3.44 Ang.
```

Alert level G

```
PLAT002_ALERT_2_G Number of Distance or Angle Restraints on AtSite         30 Note
PLAT005_ALERT_5_G No Embedded Refinement Details found  in the CIF     Please Do !
PLAT033_ALERT_4_G Flack x Value Deviates > 3.0 * sigma from Zero .      0.350 Note
PLAT045_ALERT_1_G Calculated and Reported Z Differ by a Factor ...       0.50 Check
PLAT301_ALERT_3_G Main Residue  Disorder ..............(Resd  1)..         41 % Note
PLAT302_ALERT_4_G Anion/Solvent/Minor-Residue Disorder (Resd  3)..         18 % Note
PLAT302_ALERT_4_G Anion/Solvent/Minor-Residue Disorder (Resd  5)..        100 % Note
PLAT304_ALERT_4_G Non-Integer Number of Atoms (  49.24) in Resd. #          3 Check
PLAT304_ALERT_4_G Non-Integer Number of Atoms (   3.76) in Resd. #          5 Check
PLAT720_ALERT_4_G Number of Unusual/Non-Standard Labels ..........         29 Note
PLAT773_ALERT_2_G Check long C-C Bond in CIF: C40B   --  C41B    .       1.78 Ang.
PLAT860_ALERT_3_G Number of Least-Squares Restraints ............          43 Note
```

---

0 **ALERT level A** = Most likely a serious problem - resolve or explain
0 **ALERT level B** = A potentially serious problem, consider carefully
7 **ALERT level C** = Check. Ensure it is not caused by an omission or oversight
12 **ALERT level G** = General information/check it is not something unexpected

1 ALERT type 1 CIF construction/syntax error, inconsistent or missing data
4 ALERT type 2 Indicator that the structure model may be wrong or deficient
6 ALERT type 3 Indicator that the structure quality may be low
7 ALERT type 4 Improvement, methodology, query or suggestion
1 ALERT type 5 Informative message, check

It is advisable to attempt to resolve as many as possible of the alerts in all categories. Often the minor alerts point to easily fixed oversights, errors and omissions in your CIF or refinement strategy, so attention to these fine details can be worthwhile. In order to resolve some of the more serious problems it may be necessary to carry out additional measurements or structure refinements. However, the purpose of your study may justify the reported deviations and the more serious of these should normally be commented upon in the discussion or experimental section of a paper or in the "special_details" fields of the CIF. checkCIF was carefully designed to identify outliers and unusual parameters, but every test has its limitations and alerts that are not important in a particular case may appear. Conversely, the absence of alerts does not guarantee there are no aspects of the results needing attention. It is up to the individual to critically assess their own results and, if necessary, seek expert advice.

Publication of your CIF in IUCr journals

A basic structural check has been run on your CIF. These basic checks will be run on all CIFs submitted for publication in IUCr journals (*Acta Crystallographica*, *Journal of Applied Crystallography*, *Journal of Synchrotron Radiation*); however, if you intend to submit to *Acta Crystallographica Section C* or *E* or *IUCrData*, you should make sure that full publication checks are run on the final version of your CIF prior to submission.

Publication of your CIF in other journals

Please refer to the *Notes for Authors* of the relevant journal for any special instructions relating to CIF submission.

PLATON version of 27/03/2017; check.def file version of 24/03/2017

Datablock 5B_TBACl - ellipsoid plot

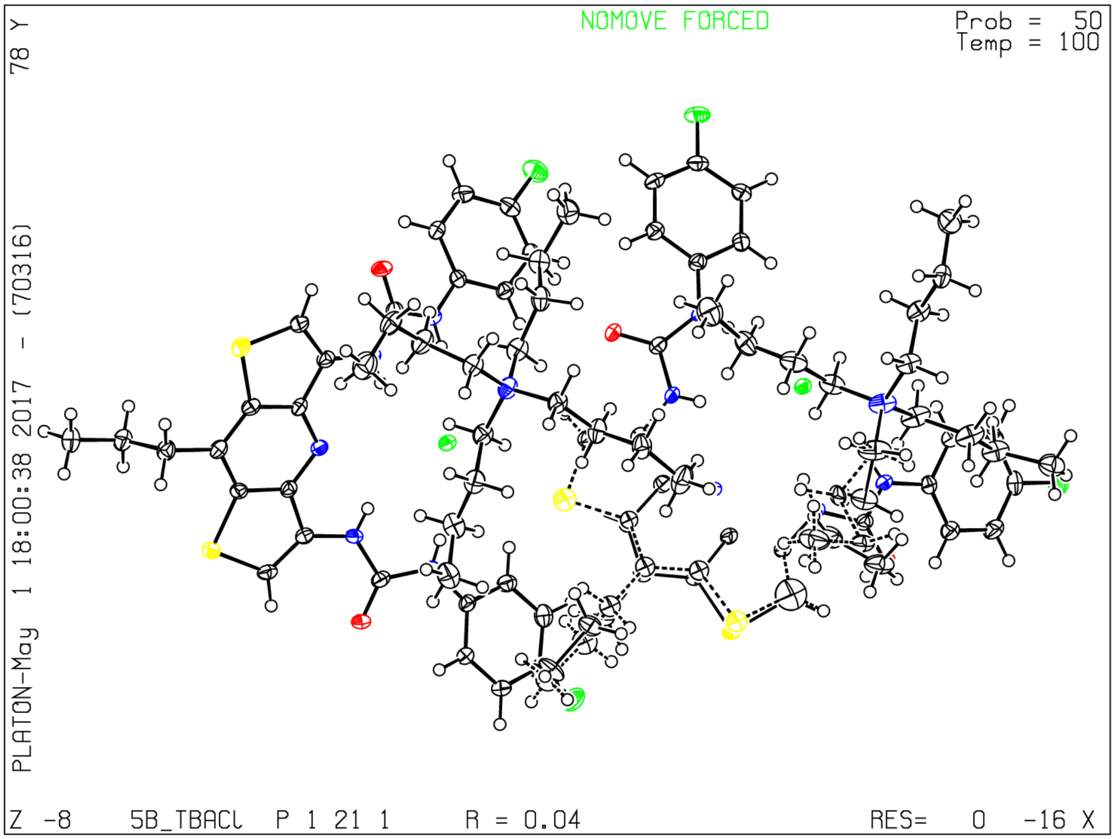

Supplement: Supplementary file 1 [file molecules-26-01788-s001.zip › 6_(Cl)TBA-checkcif.pdf]
