# Supplementary material for: Solution and Solid State Studies of Urea Derivatives of DITIPIRAM Acting as Powerful Anion Receptors
Source: Molecules. 2021 Mar 22;26(6):1788. doi: 10.3390/molecules26061788 (PMC8004752; doi:10.3390/molecules26061788)
Supplement: Supplementary file 1 [file molecules-26-01788-s001.zip › DITIPIRAM_SI.pdf]

Supporting Information

Solution and solid state studies of urea derivatives of DITIPIRAM acting as powerful anion receptors

Patryk Niedbała, Kajetan Dąbrowa, Agnieszka Cholewiak-Janusz, and Janusz Jurczak*

Institute of Organic Chemistry, Polish Academy of Sciences, Kasprzaka 44/52, 01-224 Warsaw, Poland.

E-mail: jurczak_group@icho.edu.pl

Contents

**1. CRYSTAL DATA**	**2**
**2. COMPUTATIONAL STUDIES**	**4**
**3. COPIES OF ^{1}H AND ^{13}C NMR SPECTRA**	**5**
**4. TITRATION EXPERIMENTS**	**9**
**6. CARTESIAN COORDINATES OF CALCULATED STRUCTURES**	**32**
**7. REFERENCES**	**47**

1. Crystal Data

Diffractometer and data collection

The X-ray measurements of **6**@(DMSO·H2O) and **6**@(Cl)TBA were performed at 100(2) K on a Bruker D8 Venture Photon100 diffractometer equipped with a TRIUMPH monochromator and a MoKα fine focus sealed tube (λ = 0.71073 Å) using Bruker APEX2 program [1]. Data were corrected for absorption effects using the multi-scan method (SADABS) [2]. The structure was solved and refined using SHELXTL Software Package [3].
All non-hydrogen atoms were refined anisotropically. Most of hydrogen atoms were placed in calculated positions and refined within the riding model. Position and temperature factors of four hydrogen atoms engaged in hydrogen bonds were refined. The temperature factors of all other hydrogen atoms were not refined and were set to be either 1.2 or 1.5 times larger than Ueq of the corresponding heavy atom. The atomic scattering factors were taken from the International Tables [4]. Thermal ellipsoids parameters are presented at 50% probability level.

Table S1. Crystal data and structure refinement details for **6**@(DMSO·H2O) and **6**@(Cl)TBA.

Compound	**6**@(DMSO·H2O)	**6**@(Cl)TBA
Empirical formula	$C_{28}H_{29}F_2N_5O_4S_3$	$C_{42}H_{57}ClF_2N_6O_2S_2$
Formula weight	633.74	815.50
CCDC No.	2063422	2063423
Wavelength	1.54184	1.54184
Crystal system	monoclinic	monoclinic
Space group	$P\ 2_1/n$	$P\ 2_1$
Unit cell dimensions	a = 10.2142(5) Å<br>b = 22.3169(11) Å<br>c = 12.6528(6) Å<br>β = 90.0136(12)°	a = 8.4288(4) Å<br>b = 28.6479(13) Å<br>c = 17.9789(8) Å<br>β = 99.7682(13)°
Volume	2884.2 Å^3	4278.4 Å^3
Z	4	4
Density Calc.	1.459 g/cm^3	1.266 g/cm^3
F(000)	1320	1736
Crystal size	0.104 × 0.273 × 0.559 mm	0.034 x 0.246 x 0.296 mm
Index ranges	-12 ≤ h ≤ 12<br>-26 ≤ k ≤ 26<br>-15 ≤ l ≤ 15	-10 ≤ h ≤ 10<br>-34 ≤ k ≤ 34<br>-21 ≤ l ≤ 21
Reflections collected (all / independent)	38247/5095	70952/15117
Absorption correction	multi-scan	multi-scan
Restraints / parameters	5095/407	15117/1098
Goodness-of-fit on F^2	1.040	1.028

Thermal ellipsoid plots (ORTEP) for reported crystal structures with the thermal ellipsoids shown at a 50% probability level

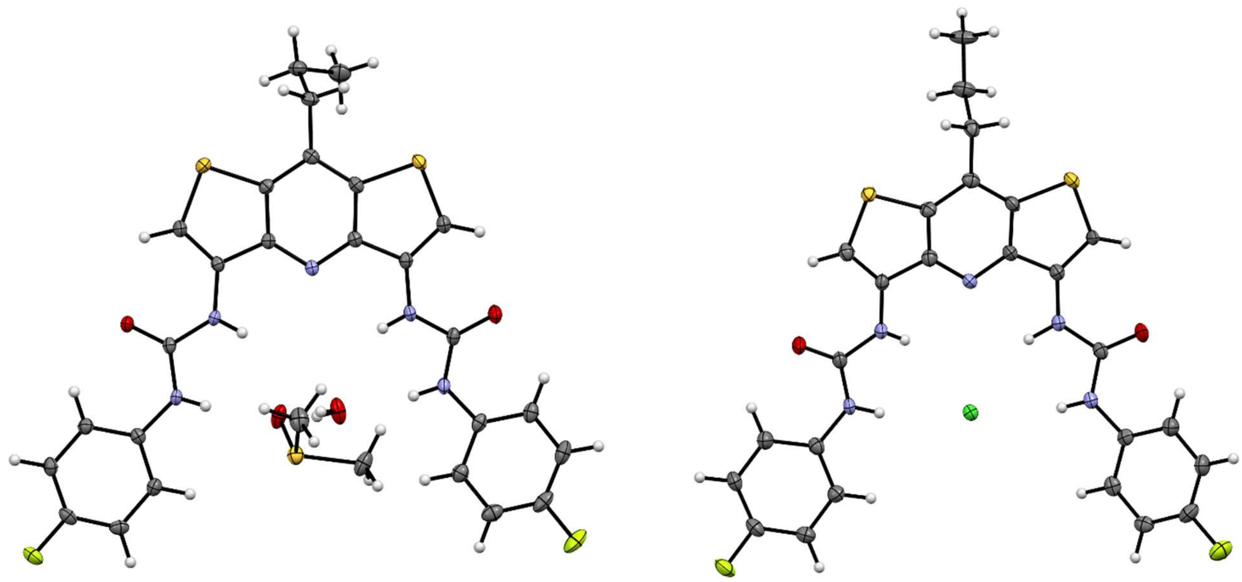

Figure S1. Thermal ellipsoid plot (ORTEP) diagram for structures **6**@(DMSO·H2O) (left) and **6**@(Cl)TBA (right).

2. Computational studies

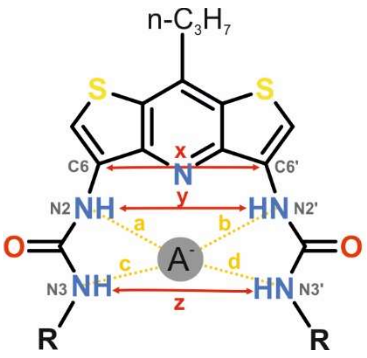

a-d - hydrogen bonds
x, y, z - distances between given atoms
R = Alk, aryl

Table S2. Relevant geometrical descriptors, hydrogen bond lengths, and calculated Gibbs free energies of energy-optimized complexes of receptors **2-7** with anions[a]

Structure	Descriptors (Å)			Hydrogen bond lengths (Å)				$\Delta(\Delta G^0)$[b]
	x	y	z	a	b	c	d	
**2**@Cl^-	4.88	4.96	6.81	3.45	3.44	3.43	3.42	-28.3
**3**@Cl^-	4.87	4.95	6.71	3.43	3.44	3.36	3.37	-31.4
**4**@Cl^-	4.87	4.94	6.67	3.42	3.42	3.34	3.35	**-39.3**
**5**@Cl^-	4.87	4.94	6.68	3.43	3.43	3.36	3.35	-34.5
**6**@Cl^-	4.87	4.94	6.70	3.43	3.43	3.36	3.36	-31.4
**6**@Cl^{-}[a]	4.82	4.87	6.54	3.39	3.46	3.29	3.30	-
**7**@Cl^-	4.87	4.94	6.68	3.46	3.45	3.35	3.36	-31.8
**2**@$MeCO_2^-$	4.92	5.14	7.30	2.86	2.86	2.85	2.87	-52.1
**3**@$MeCO_2^-$	4.96	5.32	7.65	2.83	2.82	2.75	2.75	-73.7
**4**@$MeCO_2^-$	4.96	5.31	7.62	2.82	2.81	2.73	2.73	-72.4
**5**@$MeCO_2^-$	4.92	5.16	7.27	2.86	2.85	2.80	2.78	**-75.6**
**6**@$MeCO_2^-$	4.96	5.31	7.64	2.83	2.83	2.74	2.74	-65.1
**7**@$MeCO_2^-$	4.92	5.16	7.28	2.86	2.85	2.80	2.82	-70.9
**2**@$PhCO_2^-$	4.91	5.10	7.19	2.92	2.92	2.93	2.89	-48.4
**3**@$PhCO_2^-$	4.93	5.18	7.30	2.80	2.85	2.80	2.82	-60.2
**4**@$PhCO_2^-$	4.93	5.17	7.26	2.87	2.83	2.78	2.81	**-65.3**
**5**@$PhCO_2^-$	4.93	5.18	7.29	2.88	2.84	2.78	2.81	-61.8
**6**@$PhCO_2^-$	4.91	5.12	7.19	2.86	2.90	2.83	2.83	-60.6
**7**@$PhCO_2^-$	4.92	5.14	7.25	2.87	2.87	2.83	2.85	-64.7

[a] The structures were found using conformational search analysis; the final geometry optimization (without constrains) and energy calculations was calculated at DFT/M06-2X/6-31G(d)/C-PCM:DMSO level of theory using Spartan'18 Parallel Suite. [b] Free binding energy $\Delta(\Delta G^0) = \Delta G^0_{complex} - (\Delta G^0_{receptor} + \Delta G^0_{anion})$ in kJ/mol.

3. Copies of ^{1}H and ^{13}C NMR spectra

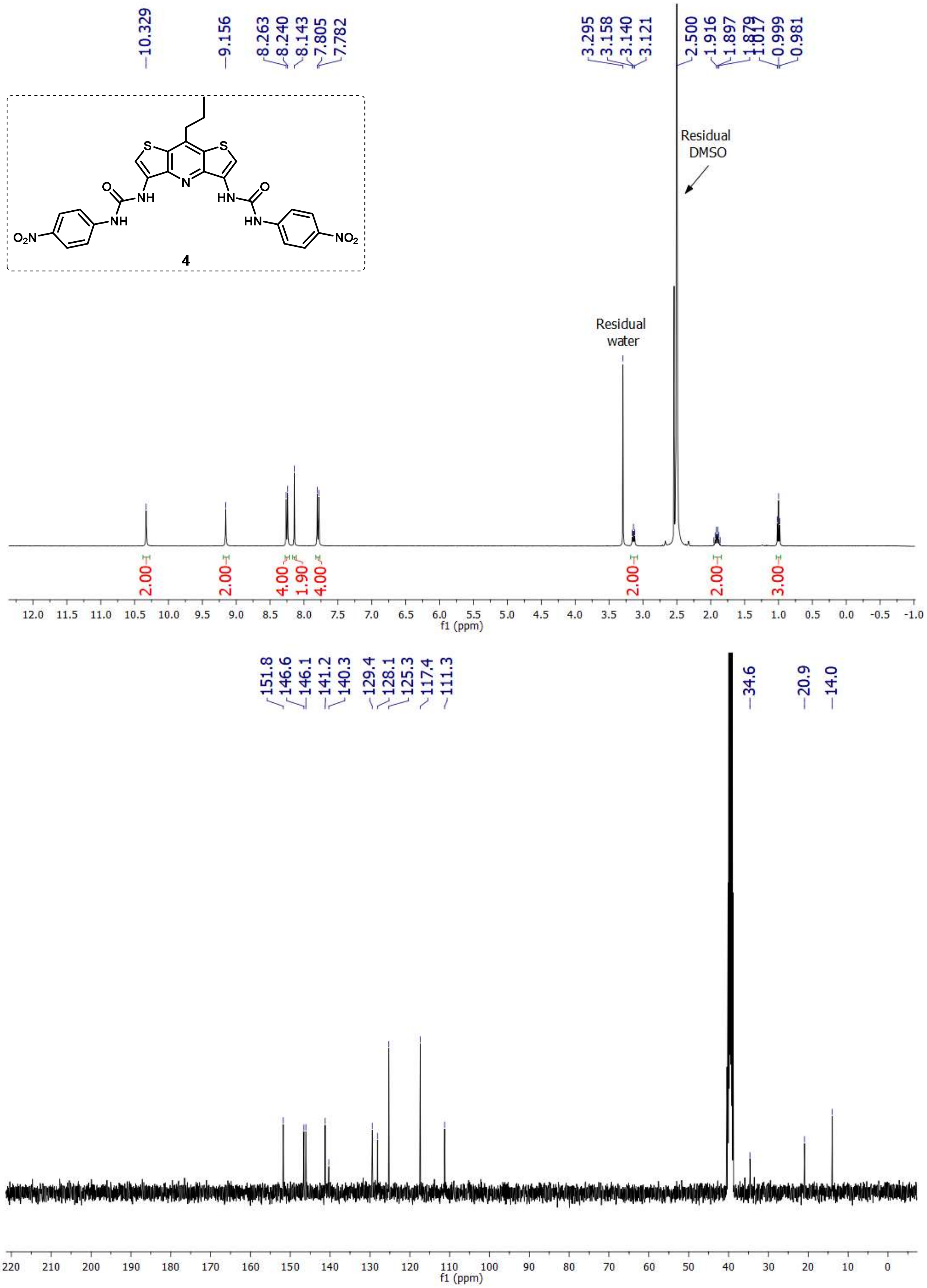

Figure S2. ^{1}H NMR (400 MHz) and ^{13}C NMR (100 MHz) spectra of compound **4** in DMSO-d_6.

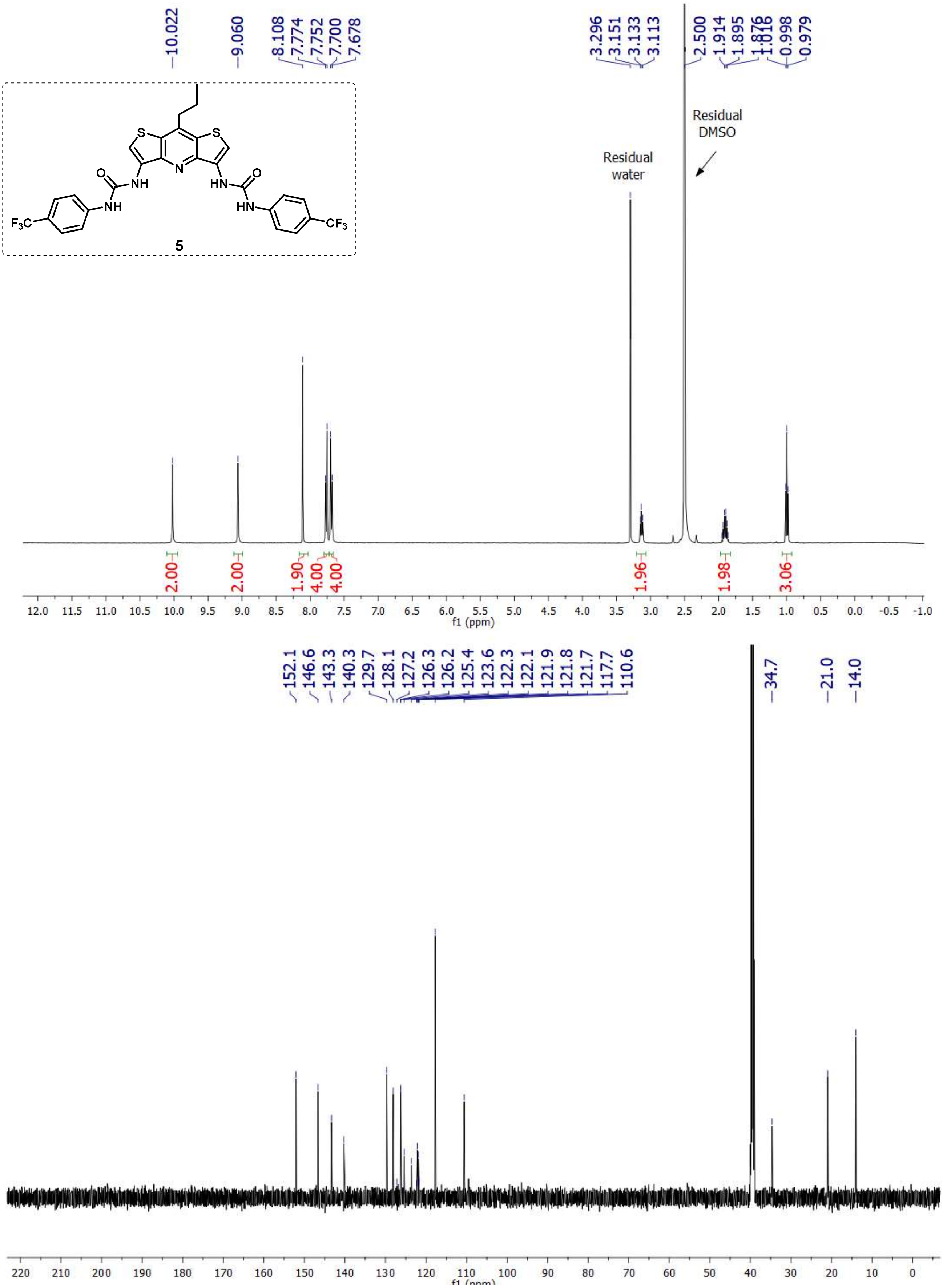

Figure S3. ^{1}H NMR (400 MHz) and ^{13}C NMR (100 MHz) spectra of compound **5** in DMSO-d_6.

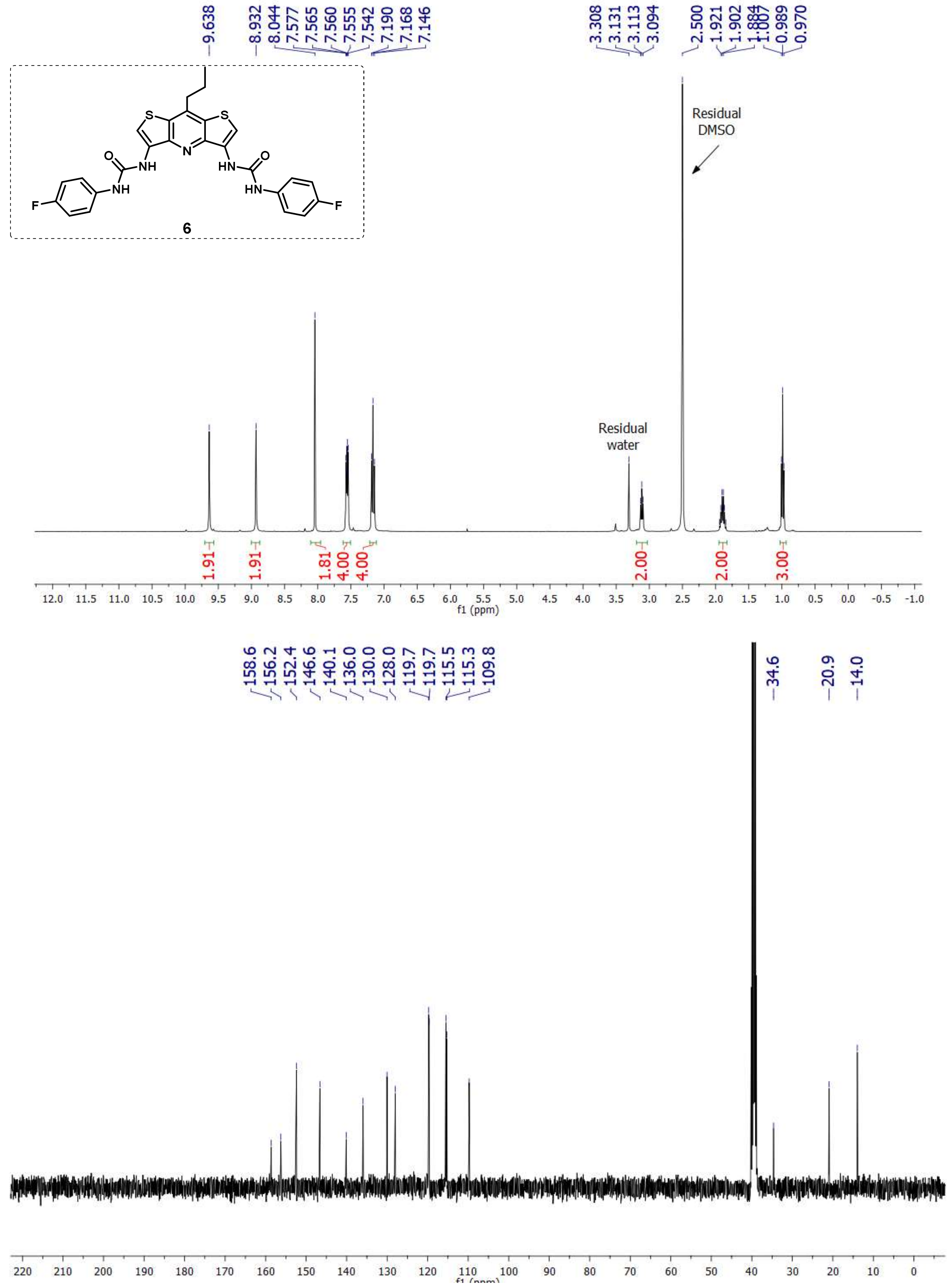

Figure S4. ^{1}H NMR (400 MHz) and ^{13}C NMR (100 MHz) spectra of compound **6** in DMSO-d_6.

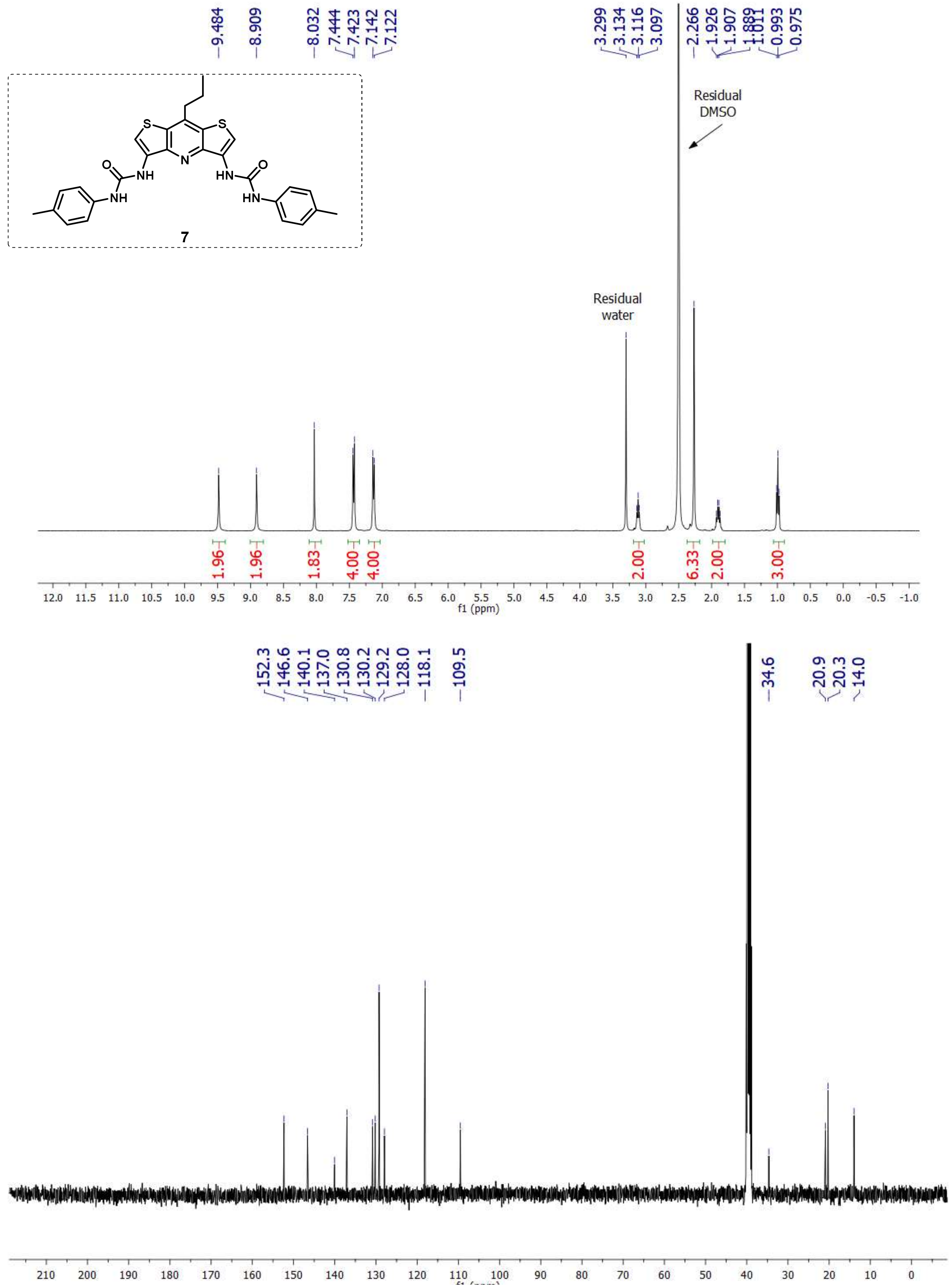

Figure S5. ^{1}H NMR (400 MHz) and ^{13}C NMR (100 MHz) spectra of compound **7** in DMSO-d_6.

4. Titration experiments

As the source of anions, commercially available tetrabutylammonium salts were used. The host solution was titrated in a NMR tube with the solution of the respective TBA salt in receptor aliquots (details are given in Table S3 and S4). The binding constants were calculated from the changes in chemical shifts of ligand protons which were shifted during titration. Nonlinear curve fitting was carried out with HypNMR 2008 [5] (Version 4.0.71) program with fitting to the appropriate global binding model (see Table S3 and S4).

Table. S3. Titration details and global stability constants K_a (M^{-1}) for Receptors **5-7** with various anions in DMSO-d_6 + 0.5% H_2O[a]

Entry	Receptor	Anion	C_{Host} [M]	C_{Guest} [M]	K_a [M^{-1}]
1	**5**	Cl^-	0.01021	0.11190	3400±80
2		$MeCO_2^-$		0.11144	>10000 [b]
3		$PhCO_2^-$		0.08957	>10000 [b]
4		$H_2PO_4^-$		0.13135	- [c]
5	**6**	Cl^-	0.01039	0.12261	4650±250
6		$MeCO_2^-$		0.09386	> 10000 [b]
7		$PhCO_2^-$		0.09747	> 10000 [b]
8		$H_2PO_4^-$		0.09582	- [c]
9	**7**	Cl^-	0.01011	0.12144	2500±50
10		$MeCO_2^-$		0.09195	> 10000 [b]
11		$H_2PO_4^-$		0.12078	- [c]

[a] Values determined by ^1H NMR titration experments at T = 303K using HypNMR 2008 software [5], errors < 10%, TBA salts as the source of anions. [b] Stability constants above the limit of the ^1H NMR titration technique (≥ 10000). [c] Not determined.

Tab. S4. Titration details and global stability constants K_a (M^{-1}) for Receptors **5-7** with various anions in DMSO-d_6 + 10% CD_3OH [a]

Entry	Receptor	Anion	C_{Host} [M]	C_{Guest} [M]	K_a [M^{-1}]
1	**5**	Cl^-	0.01024	0.10714	1040±20
2		$MeCO_2^-$		0.09991	6300±300
3		$PhCO_2^-$		0.09874	2400±80
4		$H_2PO_4^-$		0.14096	- [b]
5	**6**	Cl^-	0.01023	0.12818	1250±20
6		$MeCO_2^-$		0.09904	6400±300
7		$PhCO_2^-$		0.10073	2050±40
8		$H_2PO_4^-$		0.09361	- [b]
9	**7**	Cl^-	0.01003	0.13525	915±10
10		$MeCO_2^-$		0.09795	2950±150
11		$H_2PO_4^-$		0.10495	- [b]

[a] Values determined by ^1H NMR titration experments at T = 303K using HypNMR 2008 software [5], errors < 10%, TBA salts as the source of anions. [b] Not determined.

Figure S6. Labeling of the selected proton peaks of the receptors **5-7**.

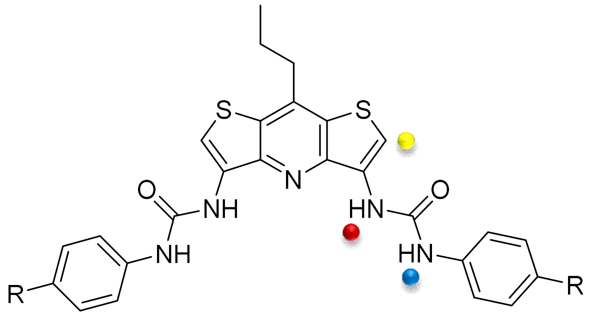

Table S4. Corresponding experimental chemical shift changes (symbols) and calculated binding isotherms (lines) assuming 1:1 binding model; Stacked plots from ^{1}H NMR titrations of Receptor **5** with increasing amount of n-Bu_4NCl in DMSO-d_6 + 0.5% H_2O (v/v).

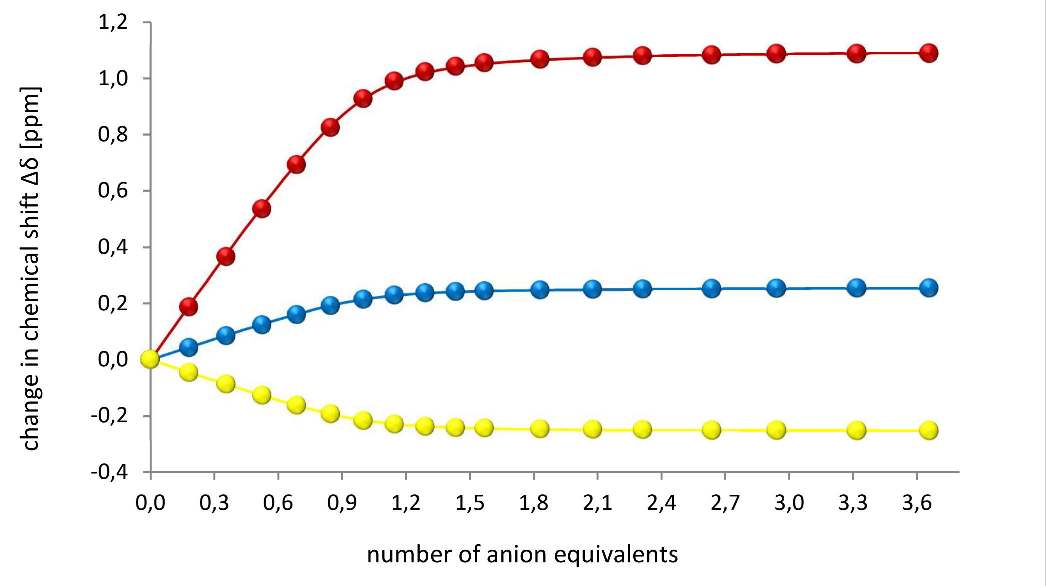

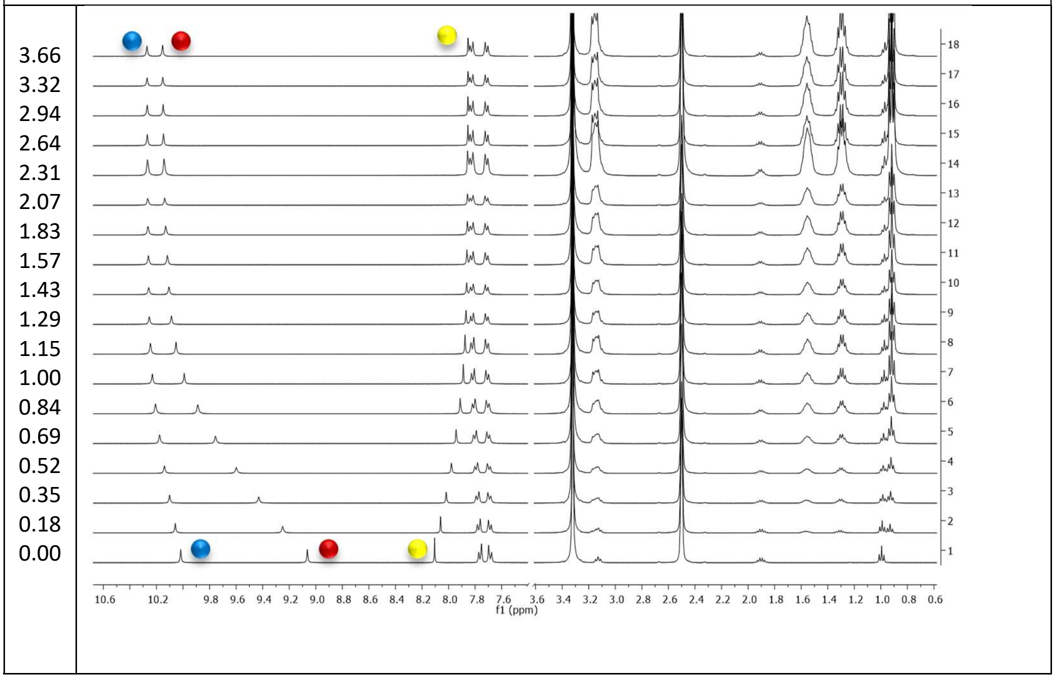

Table S5. Corresponding experimental chemical shift changes (symbols) and calculated binding isotherms (lines) assuming 1:1 binding model; Stacked plots from ^{1}H NMR titrations of Receptor **5** with increasing amount of n-Bu4NCO2Me in DMSO-d_6 + 0.5% H_2O (v/v).

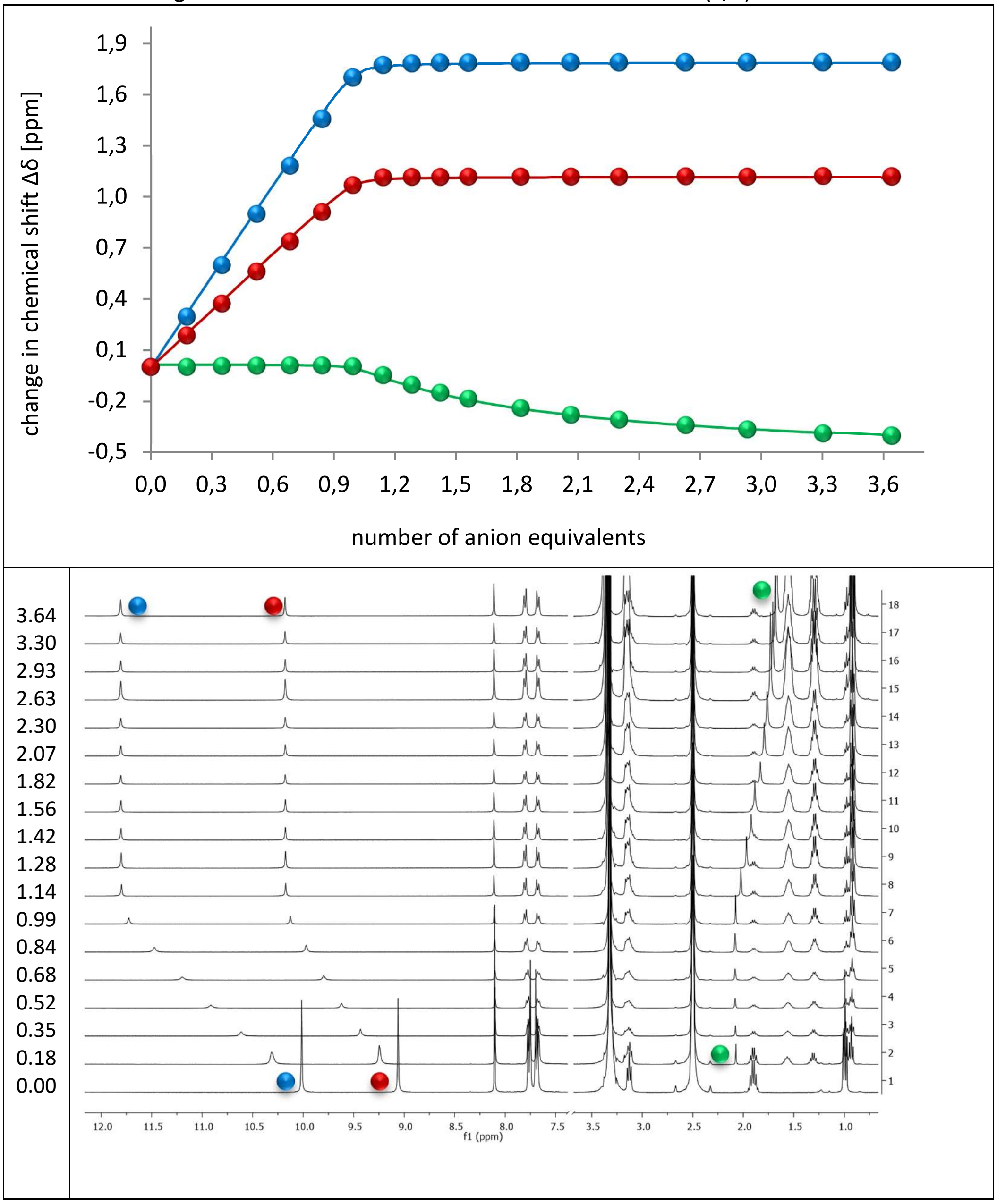

Table S6. Corresponding experimental chemical shift changes (symbols) and calculated binding isotherms (lines) assuming 1:1 binding model; Stacked plots from ^{1}H NMR titrations of Receptor **5** with increasing amount of n-Bu_4NCO_2Ph in DMSO-d_6 + 0.5% H_2O (v/v).

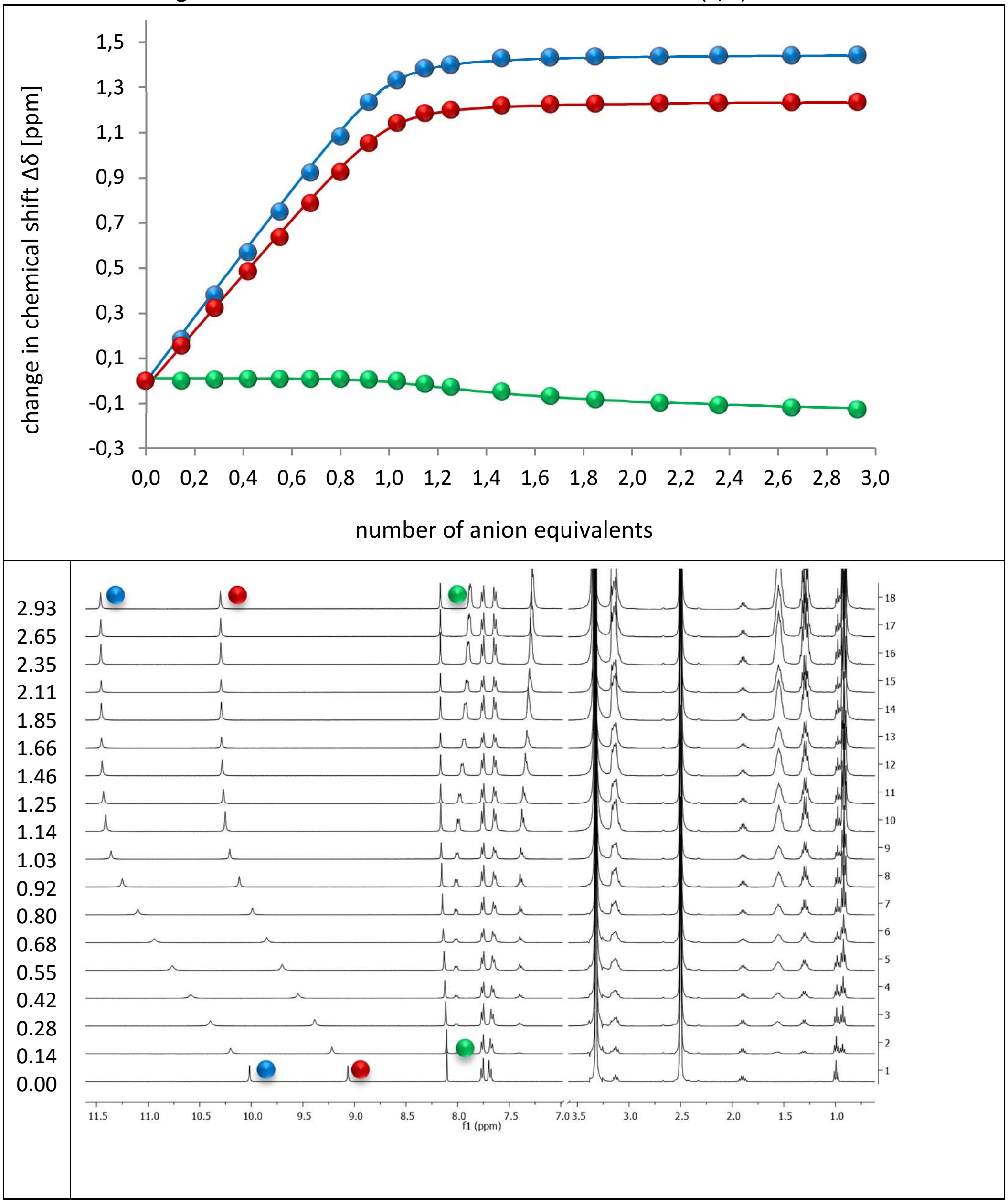

Table S7. Corresponding experimental chemical shift changes (symbols) and calculated binding isotherms (lines) assuming 1:1 binding model; Stacked plots from ^{1}H NMR titrations of Receptor **5** with increasing amount of n-$Bu_4NH_2PO_4$ in DMSO-d_6 + 0.5% H_2O (v/v).

<table>
<tr><td colspan="2">Fitting failed due to a complex binding model</td></tr>
<tr><td>4.26
3.86
3.43
3.07
2.69
2.42
2.13
1.83
1.67
1.50
1.34
1.16
0.98
0.80
0.61
0.41
0.21
0.00</td><td>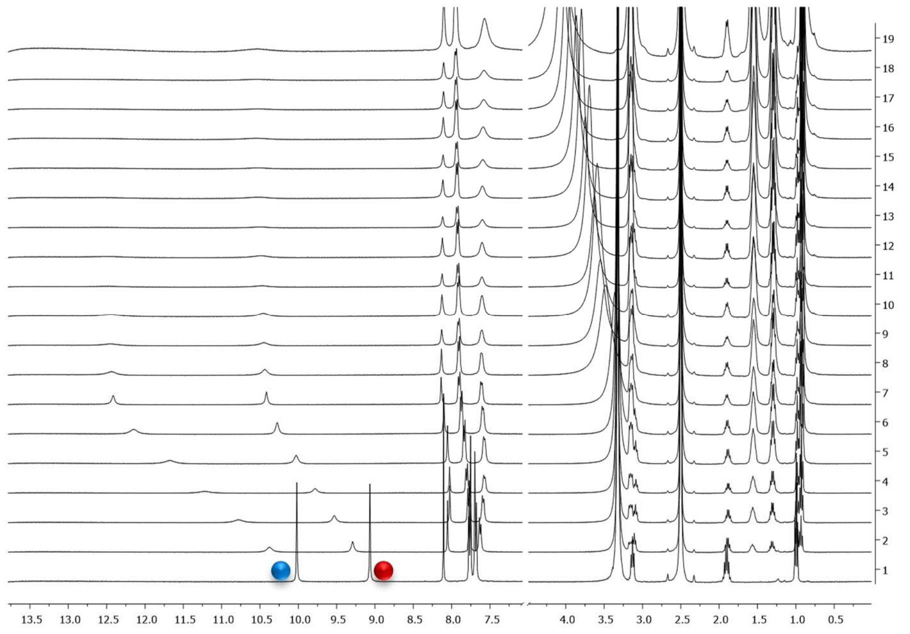
</td></tr>
</table>

Table S8. Corresponding experimental chemical shift changes (symbols) and calculated binding isotherms (lines) assuming 1:1 binding model; Stacked plots from ^{1}H NMR titrations of Receptor **5** with increasing amount of n-Bu_4NCl in DMSO-d_6 + 10% CD_3OH (v/v).

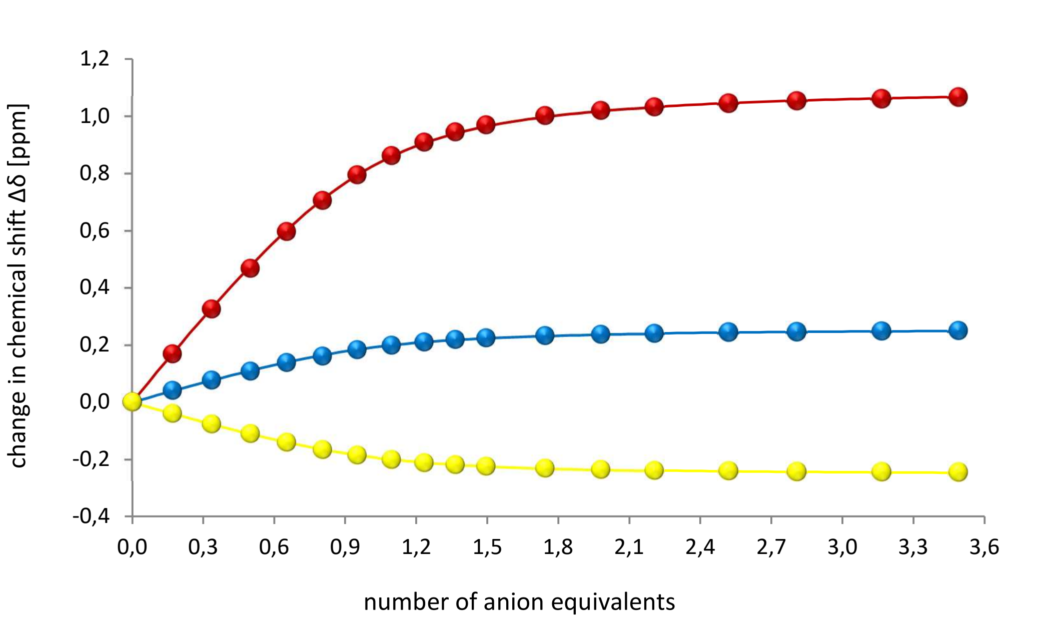

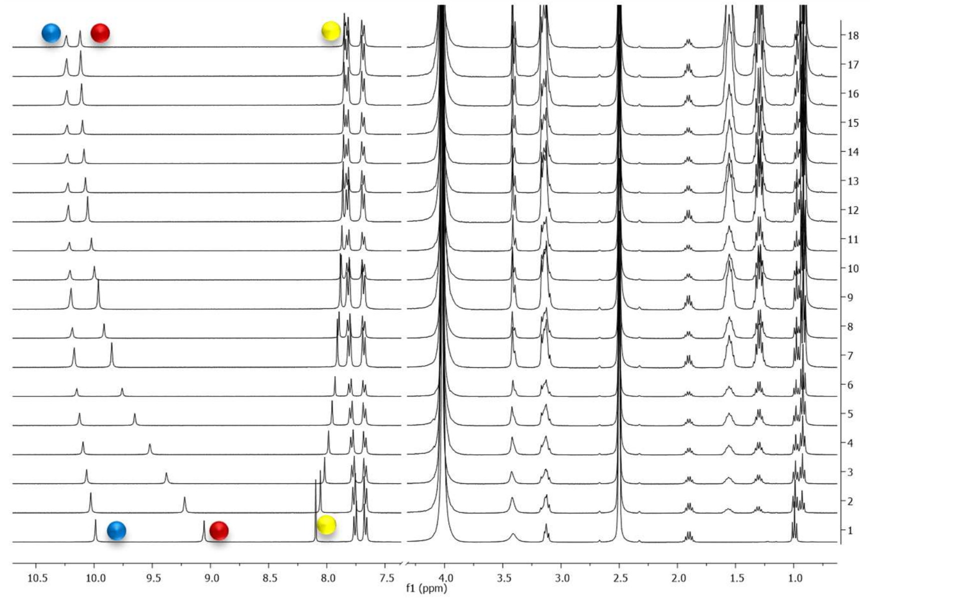

Table S9. Corresponding experimental chemical shift changes (symbols) and calculated binding isotherms (lines) assuming 1:1 binding model; Stacked plots from ^{1}H NMR titrations of Receptor **5** with increasing amount of n-Bu4NCO$_2$Me in DMSO-d_6 + 10% CD_3OH (v/v).

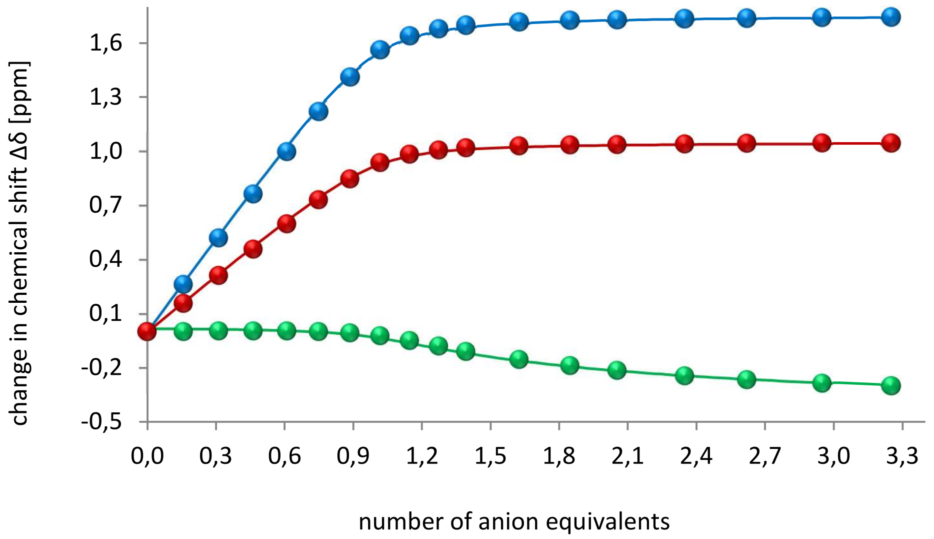

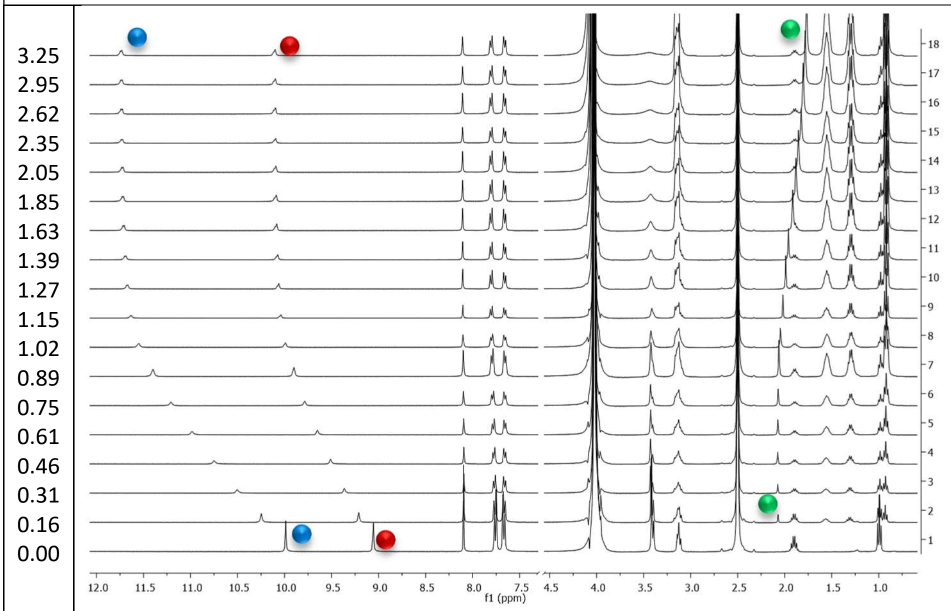

Table S10. Corresponding experimental chemical shift changes (symbols) and calculated binding isotherms (lines) assuming 1:1 binding model; Stacked plots from ^{1}H NMR titrations of Receptor **5** with increasing amount of n-Bu_4NCO_2Ph in DMSO-d_6 + 10% CD_3OH (v/v).

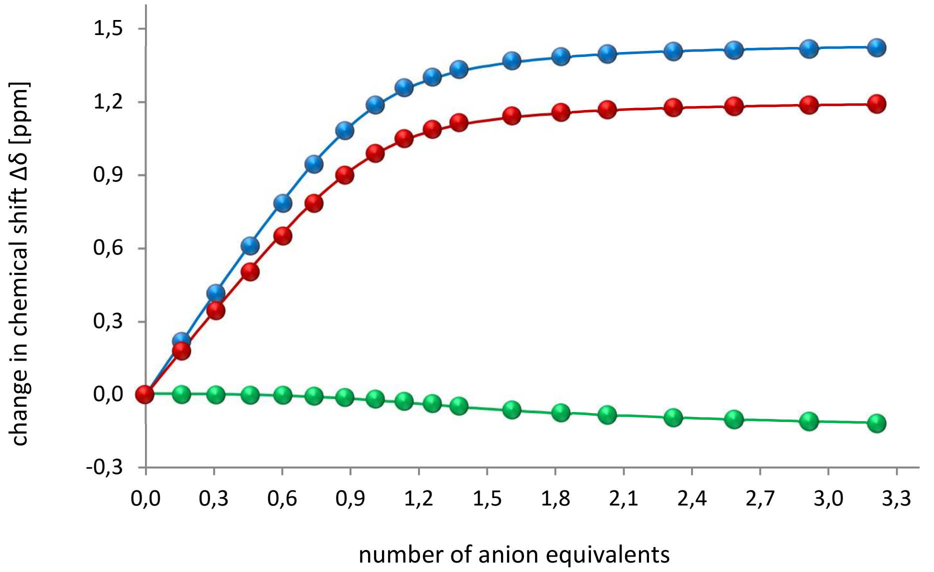

3.21
2.92
2.59
2.32
2.03
1.82
1.61
1.38
1.26
1.13
1.01
0.88
0.74
0.60
0.46
0.31
0.16
0.00

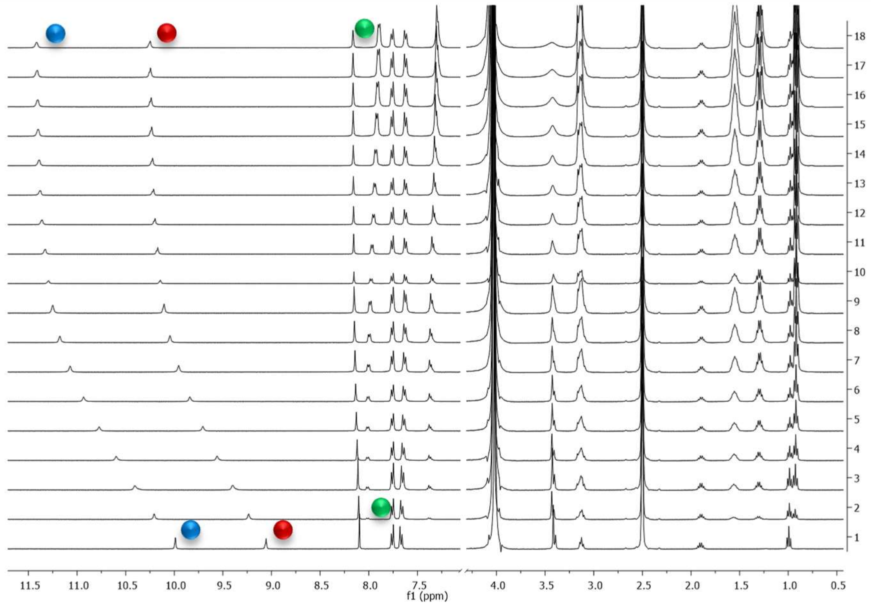

Table S11. Corresponding experimental chemical shift changes (symbols) and calculated binding isotherms (lines) assuming 1:1 binding model; Stacked plots from ^{1}H NMR titrations of Receptor **5** with increasing amount of n-$Bu_4NH_2PO_4$ in DMSO-d_6 + 10% CD_3OH (v/v).

Fitting failed due to a complex binding model	
5.01<br>4.62<br>4.19<br>3.72<br>3.33<br>2.92<br>2.62<br>2.31<br>1.98<br>1.81<br>1.63<br>1.45<br>1.26<br>1.07<br>0.87<br>0.66<br>0.45<br>0.23<br>0.00	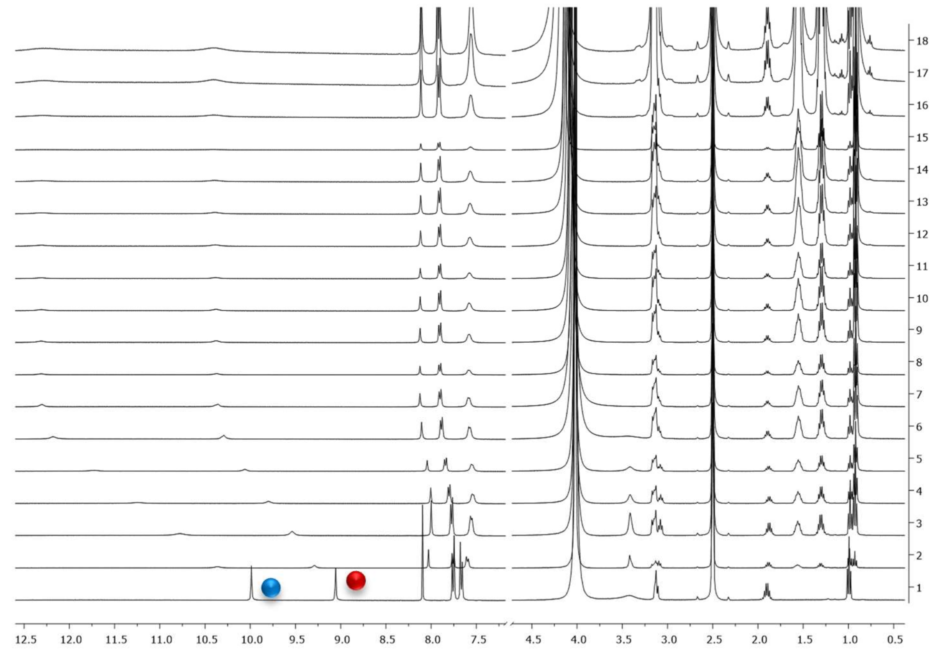

Table S12. Corresponding experimental chemical shift changes (symbols) and calculated binding isotherms (lines) assuming 1:1 binding model; Stacked plots from ^{1}H NMR titrations of Receptor **6** with increasing amount of n-Bu_4NCl in DMSO-d_6 + 0.5% H_2O (v/v).

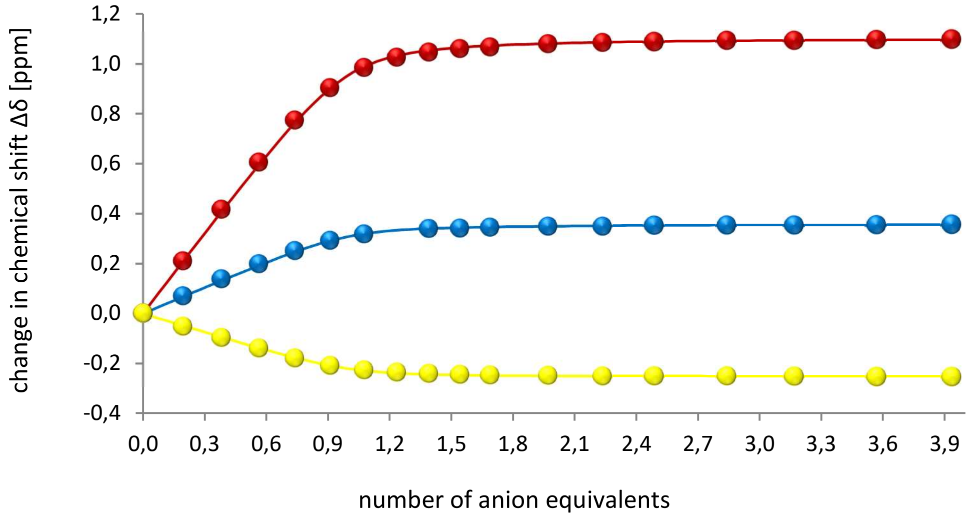

3.93
3.57
3.17
2.84
2.48
2.23
1.97
1.69
1.54
1.39
1.23
1.07
0.91
0.74
0.56
0.38
0.19
0.00

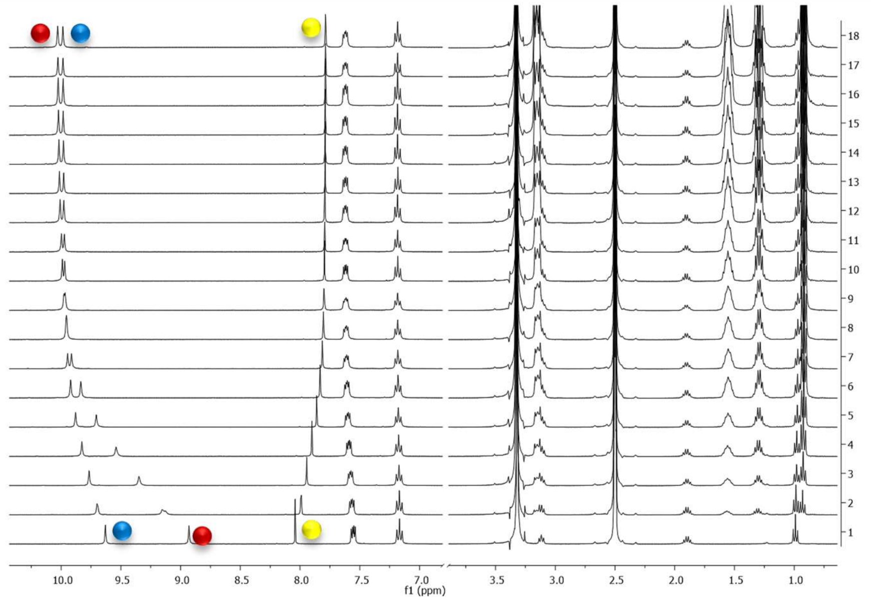

Table S13. Corresponding experimental chemical shift changes (symbols) and calculated binding isotherms (lines) assuming 1:1 binding model; Stacked plots from ^{1}H NMR titrations of Receptor **6** with increasing amount of n-Bu4NCO$_2$Me in DMSO-*d$_6$* + 0.5% H_2O (v/v).

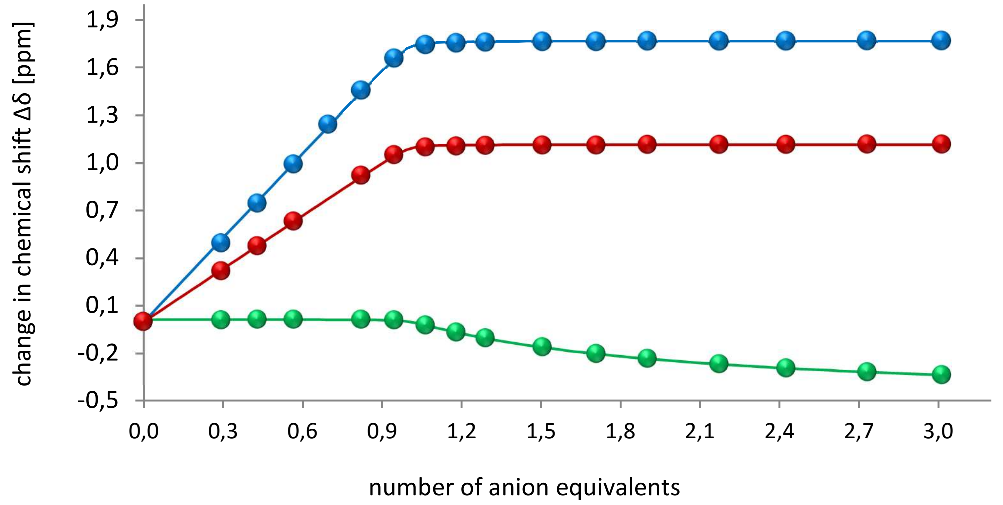

3.01
2.73
2.42
2.17
1.90
1.71
1.51
1.29
1.18
1.06
0.94
0.82
0.69
0.56
0.43
0.29
0.15
0.00

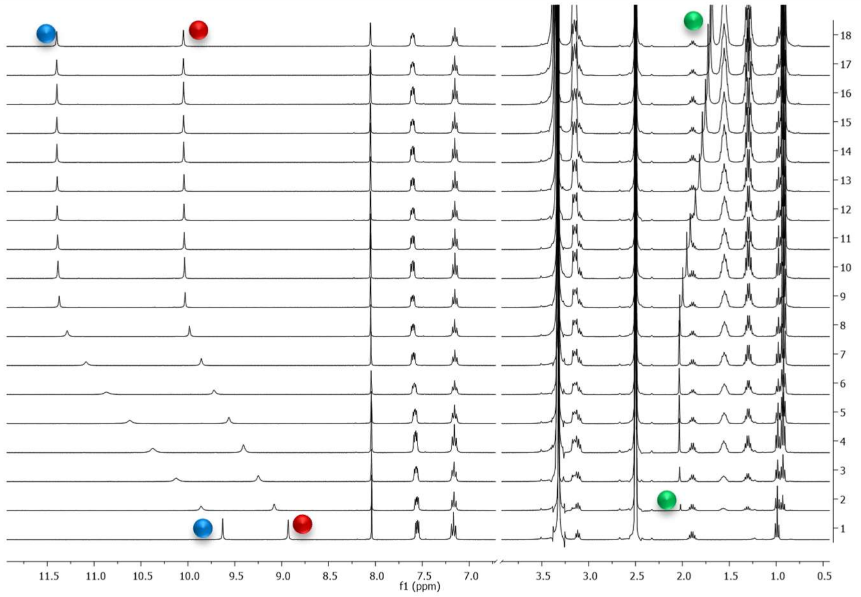

Table S14. Corresponding experimental chemical shift changes (symbols) and calculated binding isotherms (lines) assuming 1:1 binding model; Stacked plots from ^{1}H NMR titrations of Receptor **6** with increasing amount of n-Bu_4NCO_2Ph in DMSO-*d*$_6$ + 0.5% H_2O (v/v).

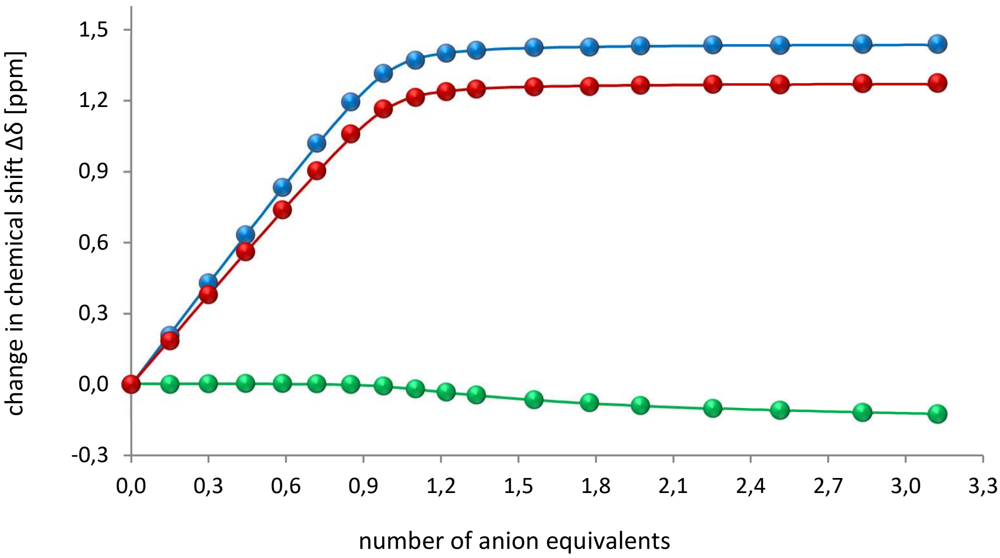

3.13
2.83
2.52
2.25
1.97
1.77
1.56
1.34
1.22
1.10
0.98
0.85
0.72
0.59
0.45
0.30
0.15
0.00

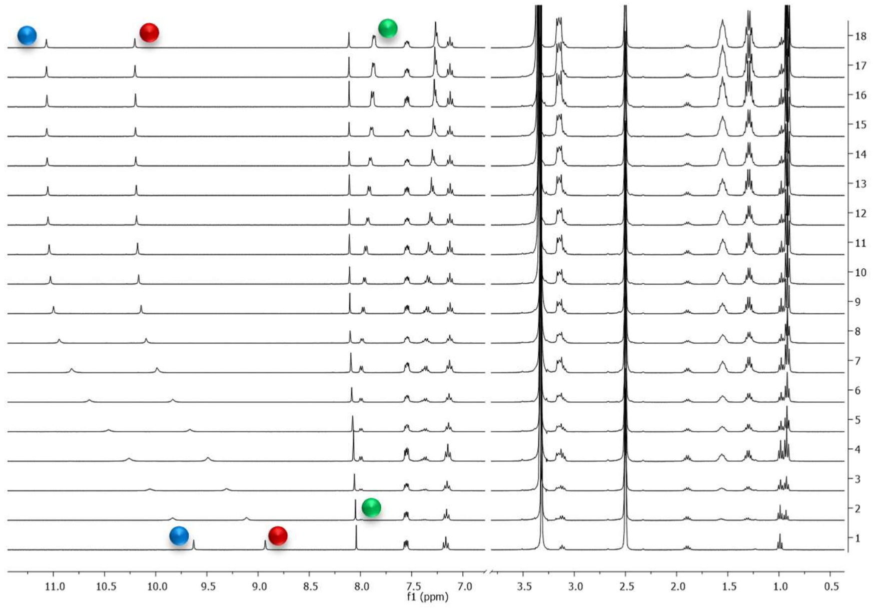

Table S15. Corresponding experimental chemical shift changes (symbols) and calculated binding isotherms (lines) assuming 1:1 binding model; Stacked plots from ^{1}H NMR titrations of Receptor **6** with increasing amount of n-$Bu_4NH_2PO_4$ in DMSO-*d_6* + 0.5% H_2O (v/v).

Fitting failed due to a complex binding model	
3.07<br>2.79<br>2.47<br>2.22<br>1.94<br>1.74<br>1.54<br>1.32<br>1.20<br>1.08<br>0.96<br>0.84<br>0.71<br>0.58<br>0.44<br>0.30<br>0.15<br>0.00	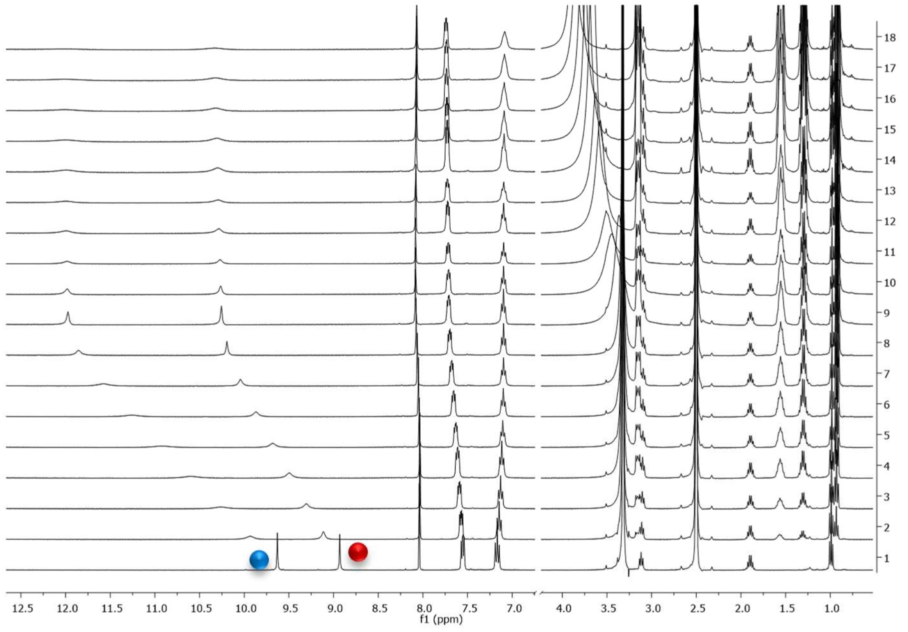

Table S16. Corresponding experimental chemical shift changes (symbols) and calculated binding isotherms (lines) assuming 1:1 binding model; Stacked plots from ^{1}H NMR titrations of Receptor **6** with increasing amount of n-Bu_4NCl in DMSO-d_6 + 10% CD_3OH (v/v).

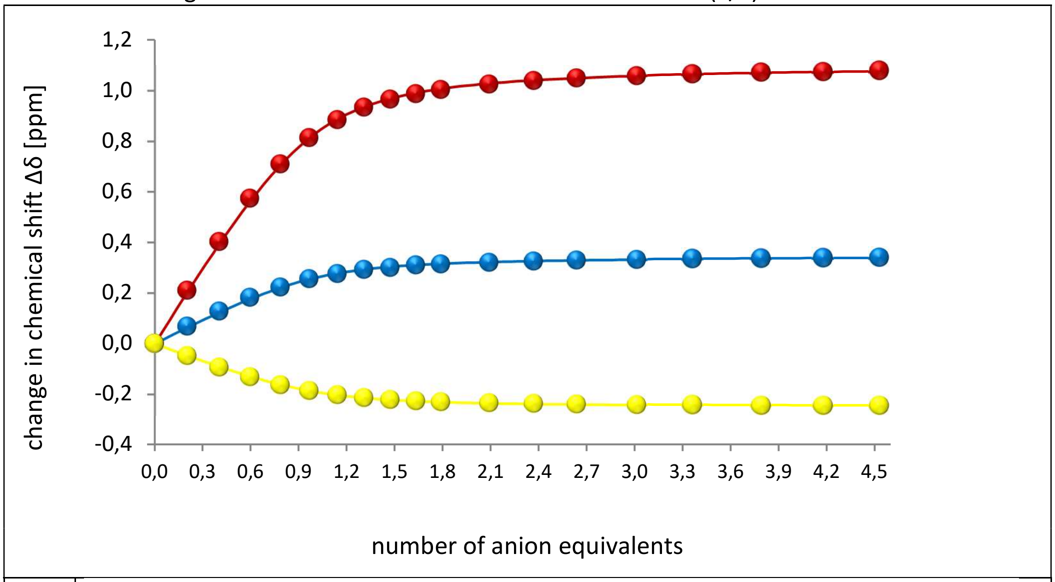

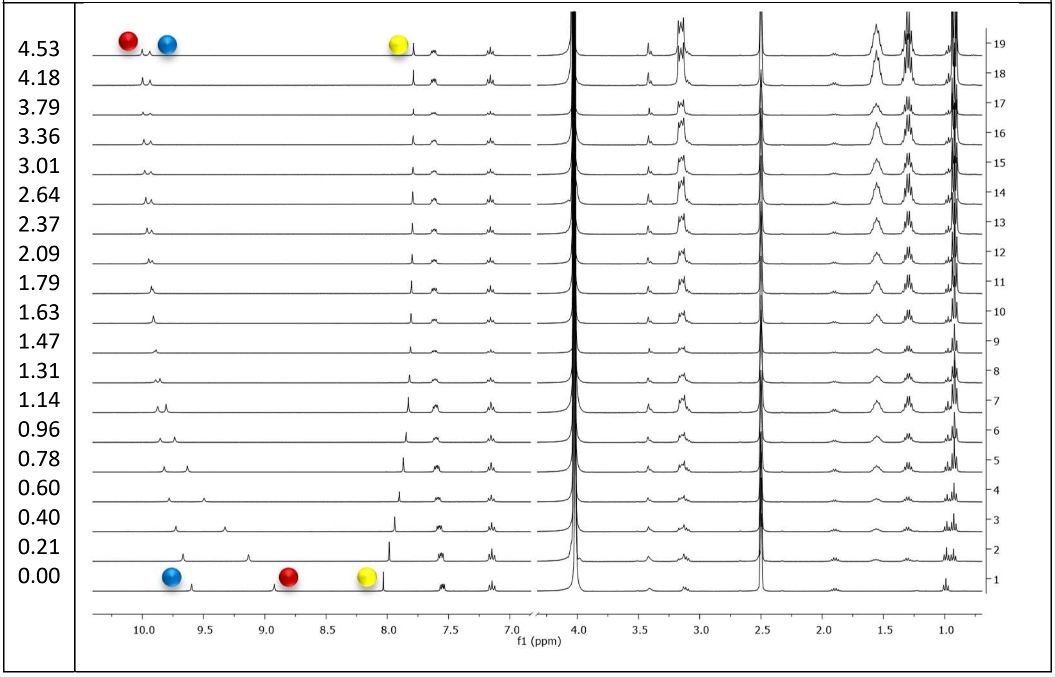

Table S17. Corresponding experimental chemical shift changes (symbols) and calculated binding isotherms (lines) assuming 1:1 binding model; Stacked plots from ^{1}H NMR titrations of Receptor **6** with increasing amount of n-Bu4NCO₂Me in DMSO-*d*₆ + 10% CD₃OH (v/v).

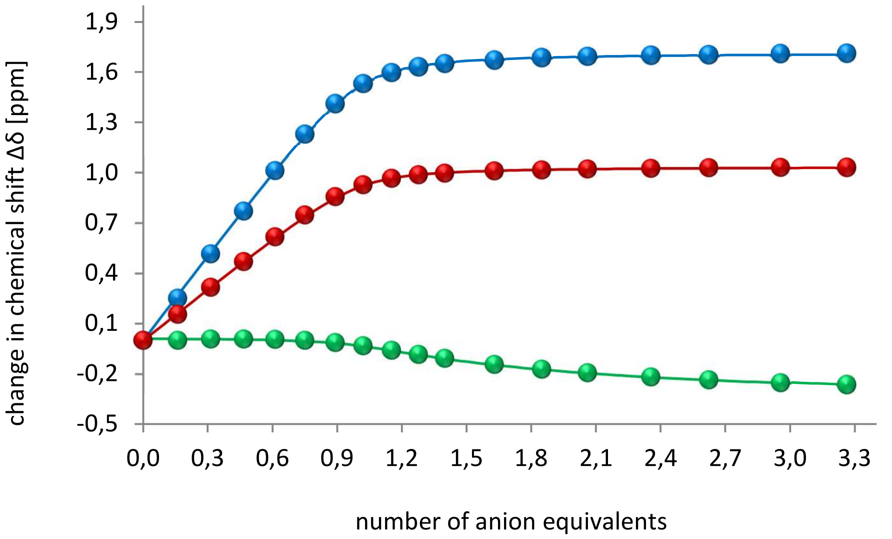

Equivalents	Spectrum
3.26<br>2.96<br>2.63<br>2.35<br>2.06<br>1.85<br>1.63<br>1.40<br>1.28<br>1.15<br>1.02<br>0.89<br>0.75<br>0.61<br>0.47<br>0.32<br>0.16<br>0.00	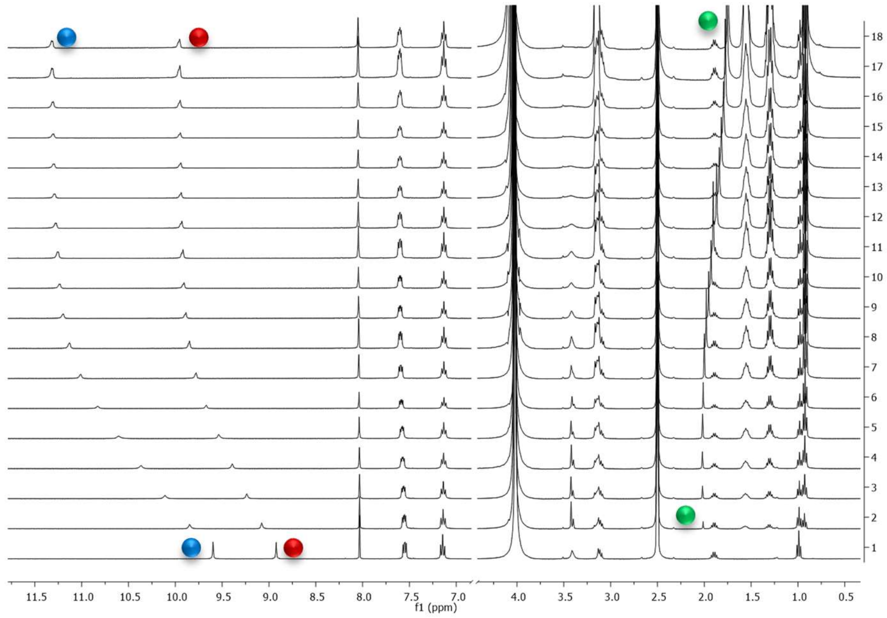

Table S18. Corresponding experimental chemical shift changes (symbols) and calculated binding isotherms (lines) assuming 1:1 binding model; Stacked plots from ^{1}H NMR titrations of Receptor **6** with increasing amount of n-Bu_4NCO_2Ph in DMSO-d_6 + 10% CD_3OH (v/v).

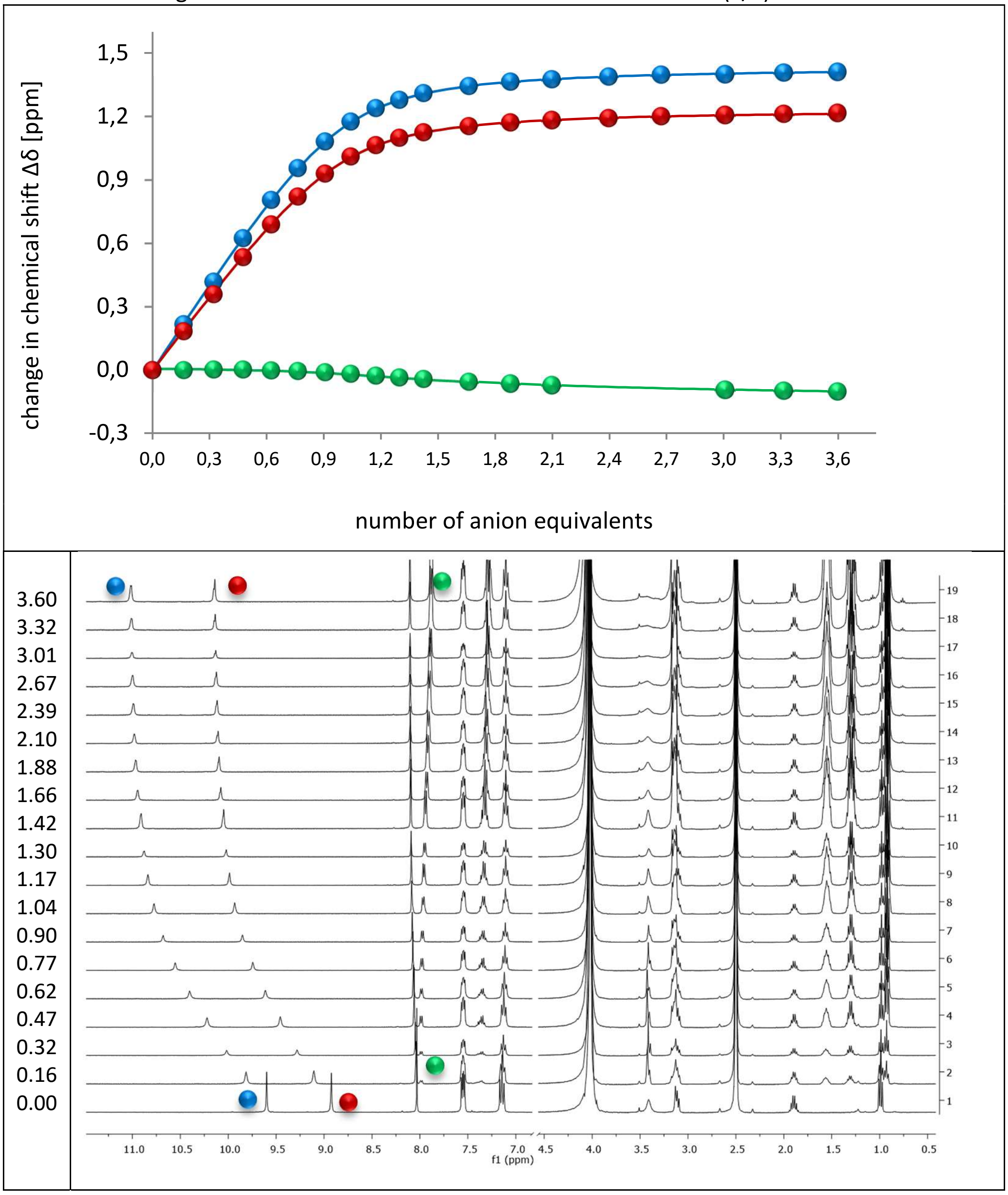

Table S19. Corresponding experimental chemical shift changes (symbols) and calculated binding isotherms (lines) assuming 1:1 binding model; Stacked plots from ^{1}H NMR titrations of Receptor **6** with increasing amount of n-$Bu_4NH_2PO_4$ in DMSO-d_6 + 10% CD_3OH (v/v).

<table>
<tr><td colspan="2">Fitting failed due to a complex binding model</td></tr>
<tr><td>3.31
3.05
2.77
2.45
2.20
1.93
1.73
1.52
1.31
1.19
1.08
0.96
0.83
0.70
0.57
0.44
0.30
0.15
0.00</td><td>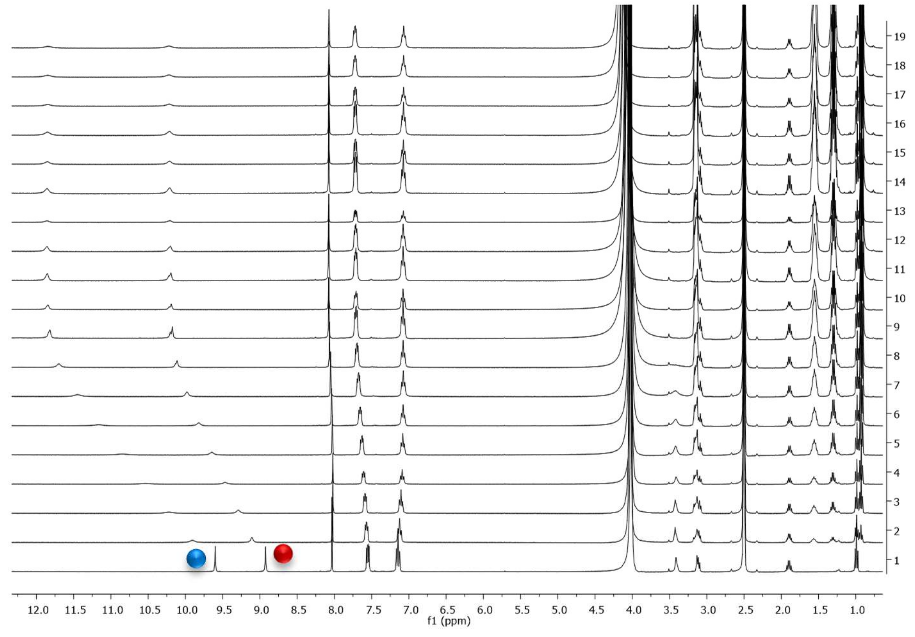
</td></tr>
</table>

Table S20. Corresponding experimental chemical shift changes (symbols) and calculated binding isotherms (lines) assuming 1:1 binding model; Stacked plots from ^{1}H NMR titrations of Receptor **7** with increasing amount of n-Bu_4NCl in DMSO-d_6 + 0.5% H_2O (v/v).

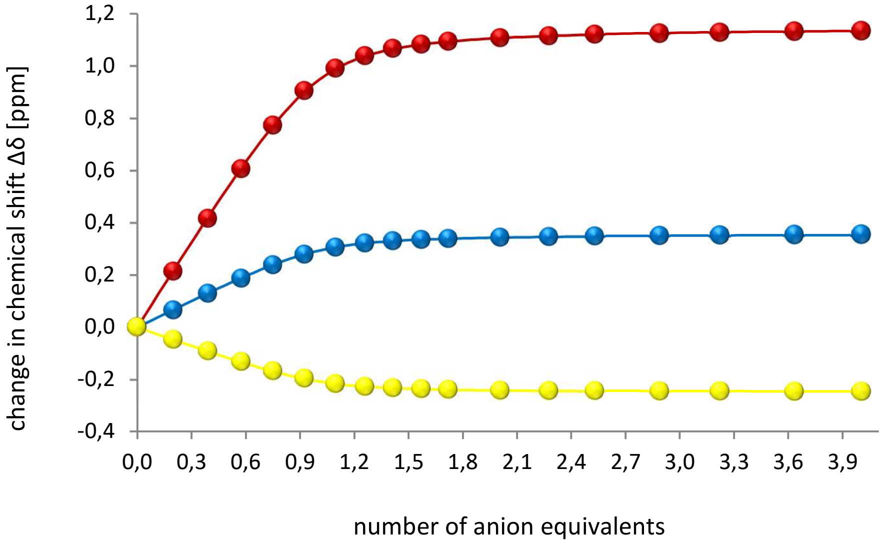

4.00
3.63
3.22
2.89
2.53
2.27
2.00
1.72
1.57
1.41
1.25
1.09
0.92
0.75
0.57
0.39
0.20
0.00

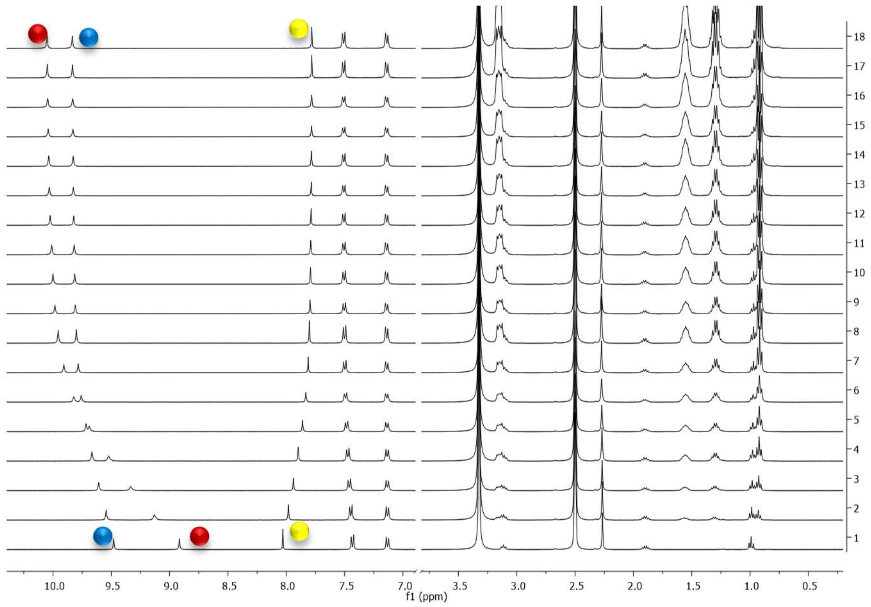

Table S21. Corresponding experimental chemical shift changes (symbols) and calculated binding isotherms (lines) assuming 1:1 binding model; Stacked plots from ^{1}H NMR titrations of Receptor **7** with increasing amount of n-Bu4NCO$_2$Me in DMSO-d_6 + 0.5% H_2O (v/v).

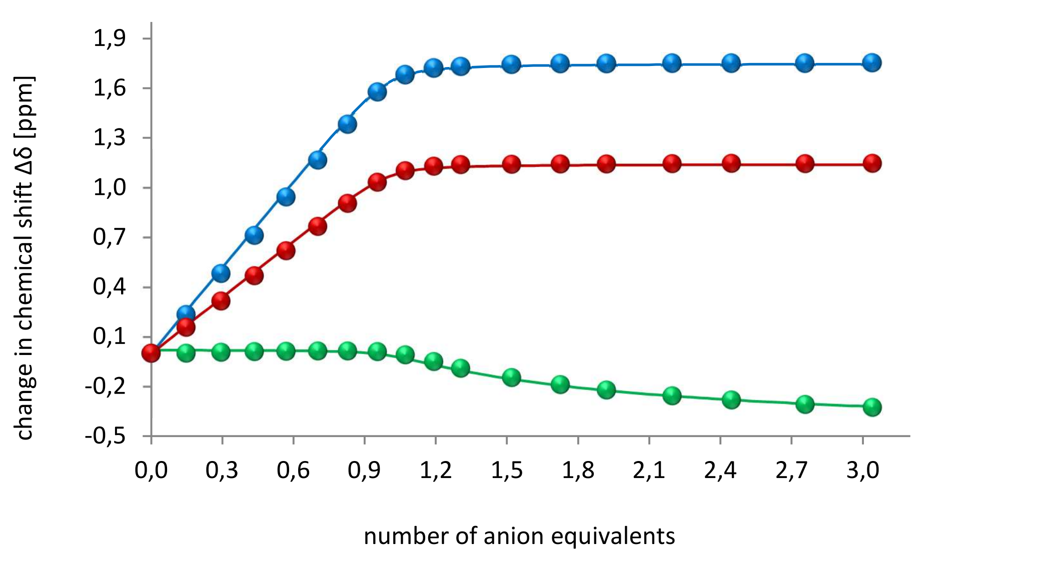

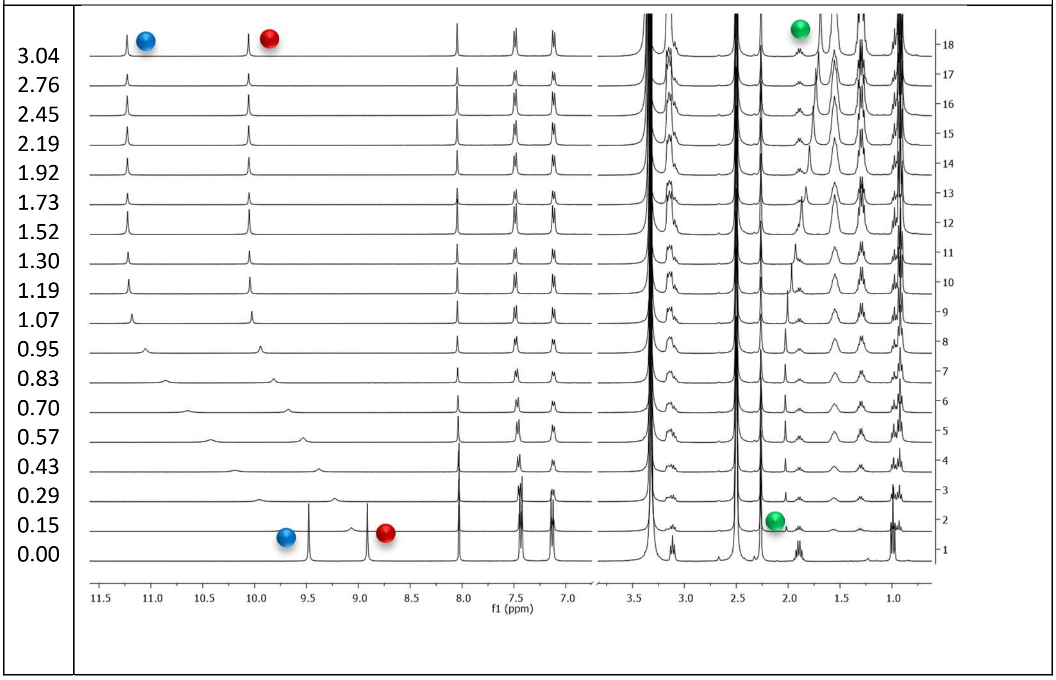

Table S22. Corresponding experimental chemical shift changes (symbols) and calculated binding isotherms (lines) assuming 1:1 binding model; Stacked plots from ^{1}H NMR titrations of Receptor **7** with increasing amount of n-$Bu_4NH_2PO_4$ in DMSO-d_6 + 0.5% H_2O (v/v).

Fitting failed due to a complex binding model	
4.32<br>3.98<br>3.61<br>3.21<br>2.87<br>2.52<br>2.26<br>1.99<br>1.71<br>1.56<br>1.41<br>1.25<br>1.09<br>0.92<br>0.75<br>0.57<br>0.39<br>0.20<br>0.00	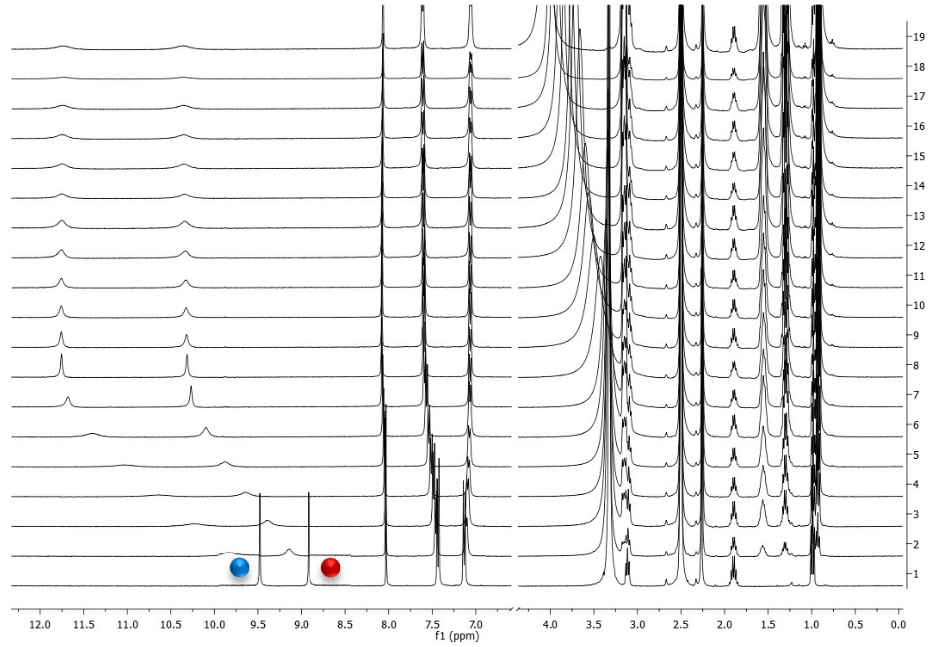

Table S23. Corresponding experimental chemical shift changes (symbols) and calculated binding isotherms (lines) assuming 1:1 binding model; Stacked plots from ^{1}H NMR titrations of Receptor **7** with increasing amount of n-Bu_4NCl in DMSO-d_6 + 10% CD_3OH (v/v).

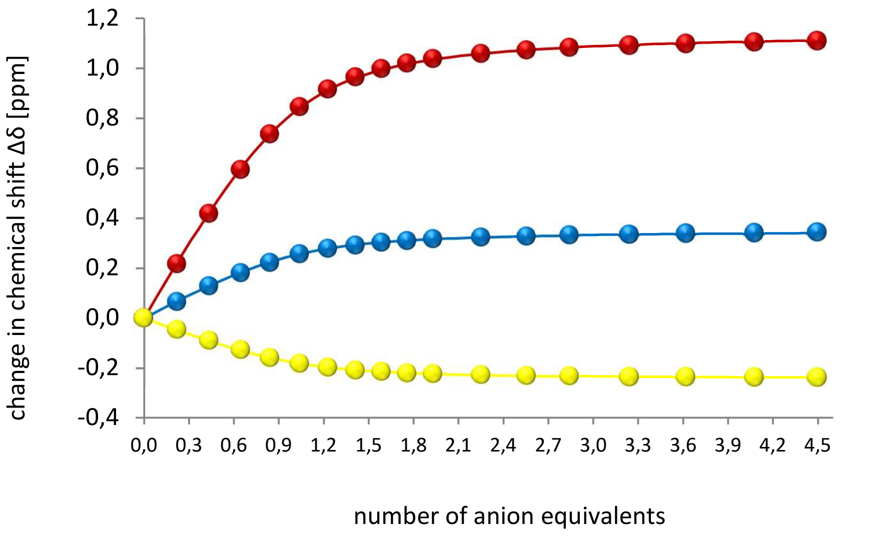

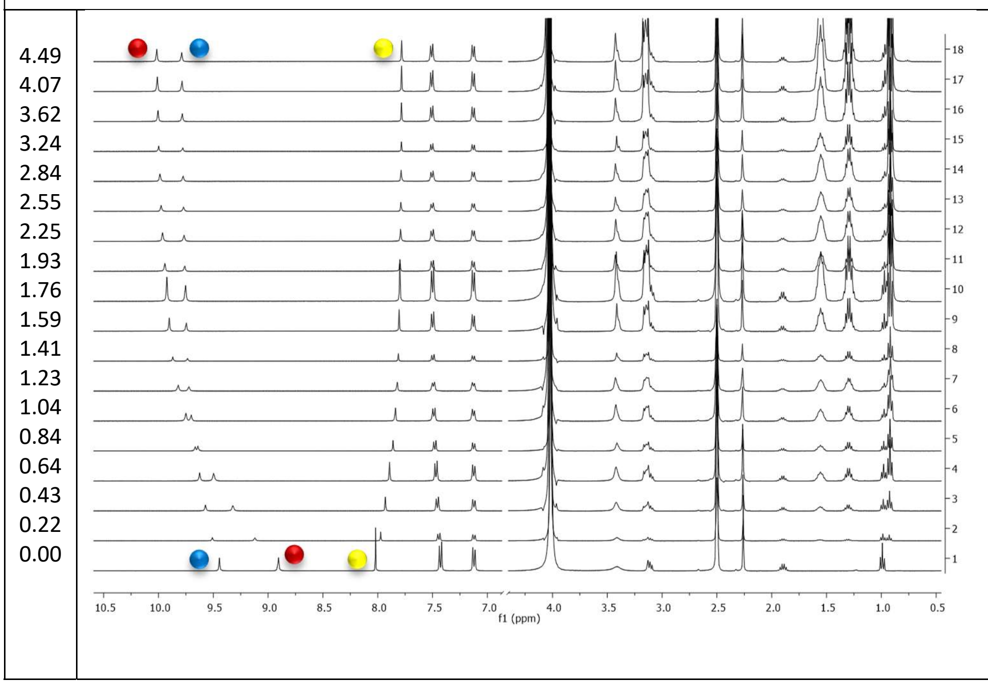

Table S24. Corresponding experimental chemical shift changes (symbols) and calculated binding isotherms (lines) assuming 1:1 binding model; Stacked plots from ^{1}H NMR titrations of Receptor **7** with increasing amount of n-Bu4NCO$_2$Me in DMSO-d_6 + 10% CD$_3$OH (v/v).

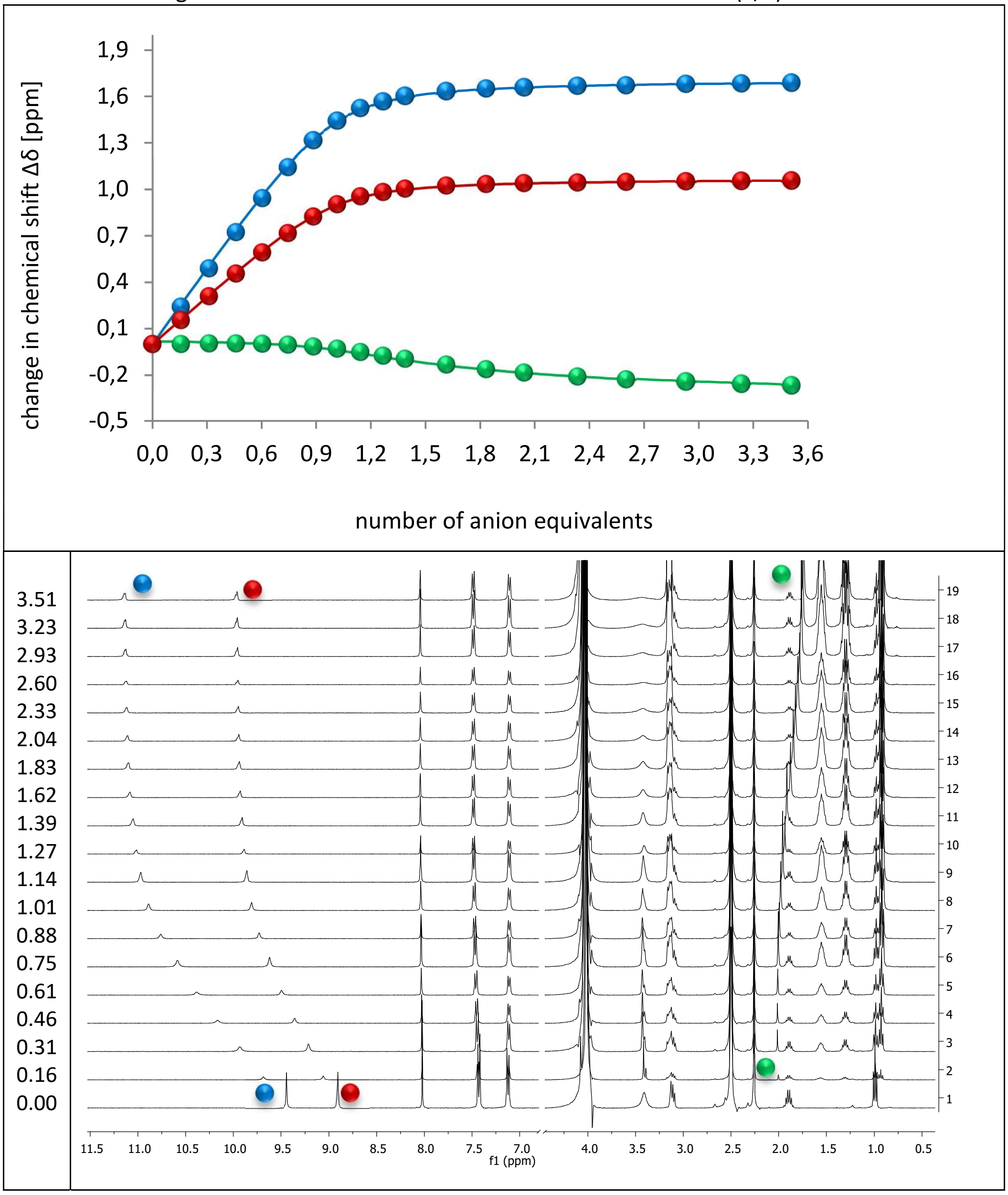

Table S25. Corresponding experimental chemical shift changes (symbols) and calculated binding isotherms (lines) assuming 1:1 binding model; Stacked plots from ^{1}H NMR titrations of Receptor **7** with increasing amount of n-$Bu_4NH_2PO_4$ in DMSO-d_6 + 10% CD_3OH (v/v).

Fitting failed due to a complex binding model

Equiv.	Spectrum
3.85<br>3.49<br>3.16<br>2.81<br>2.52<br>2.20<br>1.98<br>1.74<br>1.49<br>1.36<br>1.23<br>1.09<br>0.95<br>0.80<br>0.65<br>0.50<br>0.34<br>0.17<br>0.00	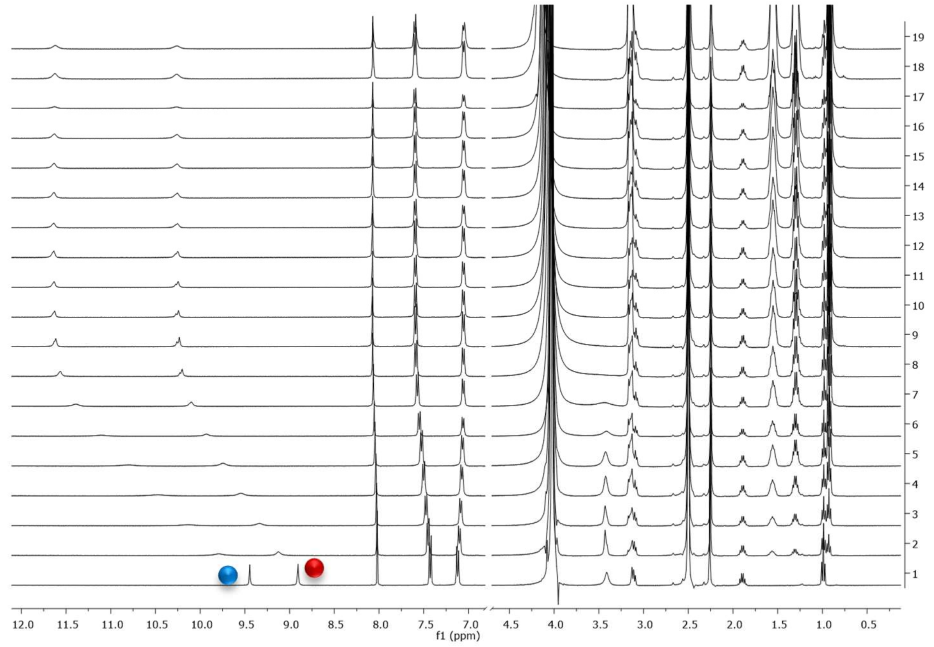

6. Cartesian coordinates of calculated structures

Table S26. Cartesian coordinates of the studied structures; all values in Hartrees (@298.15K); energies for Cl^-, $MeCO_2^-$, and $PhCO_2^-$ are -460.351275, -228.487930, and -420.111546, respectively.

Receptor **2** ΔG^0 = -2076.65198 au				Receptor **3** ΔG^0 = -2224.28751 au				Receptor **4** ΔG^0 = -2633.15986 au			
C	-0.104806	-0.001085	-4.315002	C	-0.405838	0.425268	-4.177602	C	-0.417267	0.468169	-4.602083
N	-0.610128	-0.000846	-1.471477	N	-0.261372	0.157942	-1.305093	N	-0.274284	0.198180	-1.728911
C	-0.236170	-1.172094	-3.567365	C	-0.590361	-0.791121	-3.517794	C	-0.617221	-0.745640	-3.942211
C	-0.237881	1.170016	-3.567763	C	-0.141844	1.500615	-3.327276	C	-0.139353	1.539526	-3.751417
C	-0.485842	1.126143	-2.174801	C	-0.081307	1.325771	-1.924230	C	-0.081079	1.363586	-2.348371
C	-0.483943	-1.127906	-2.174349	C	-0.510464	-0.878063	-2.107576	C	-0.536355	-0.833901	-2.532244
S	-0.137609	-2.816578	-4.136557	S	-0.927226	-2.350001	-4.223462	S	-0.975459	-2.299301	-4.648245
S	-0.140964	2.814323	-4.137688	S	0.150152	3.163883	-3.763890	S	0.173913	3.198937	-4.188186
C	-0.425619	-3.433696	-2.521103	C	-0.962985	-3.103045	-2.641586	C	-1.017262	-3.053142	-3.068440
H	-0.455766	-4.501137	-2.367338	H	-1.154322	-4.162737	-2.572380	H	-1.221316	-4.110609	-3.000061
C	-0.589869	-2.454721	-1.593073	C	-0.730132	-2.230246	-1.626991	C	-0.770649	-2.183779	-2.054561
C	-0.430612	3.431788	-2.522665	C	0.345269	3.613234	-2.082133	C	0.372741	3.646334	-2.507899
H	-0.462152	4.499245	-2.369268	H	0.559068	4.642350	-1.839052	H	0.597945	4.673183	-2.265148
C	-0.594038	2.453033	-1.594268	C	0.199229	2.567770	-1.227244	C	0.213075	2.602816	-1.653505
C	0.214845	-0.000545	-5.782163	C	-0.425338	0.553377	-5.673714	C	-0.433186	0.595392	-6.098086
H	-0.234790	0.880727	-6.256570	H	-0.791078	1.548908	-5.954405	H	-0.782352	1.596301	-6.380669
H	-0.231725	-0.882921	-6.257295	H	-1.127291	-0.175686	-6.097268	H	-1.145846	-0.123007	-6.521877
C	1.732623	0.002489	-6.033176	C	0.970180	0.332412	-6.282760	C	0.960118	0.350291	-6.702747
H	2.176688	-0.880776	-5.558067	H	1.332138	-0.663271	-5.999144	H	1.306056	-0.649285	-6.412609
H	2.174438	0.881903	-5.548917	H	1.668771	1.060779	-5.853368	H	1.668543	1.070194	-6.275242
C	2.051049	0.010921	-7.525701	C	0.941741	0.466597	-7.802891	C	0.937993	0.476287	-8.223582
H	3.131731	0.011192	-7.697606	H	1.936546	0.309924	-8.230649	H	1.931523	0.300684	-8.646902
H	1.630052	0.900409	-8.007417	H	0.598042	1.464149	-8.098232	H	0.611816	1.477550	-8.526014
H	1.628726	-0.871938	-8.018308	H	0.261548	-0.269477	-8.245524	H	0.247348	-0.251315	-8.663926
N	-0.832528	-2.542716	-0.227663	N	-0.671221	-2.440204	-0.253117	N	-0.706930	-2.399144	-0.680152
H	-0.927547	-1.651740	0.251053	H	-0.489866	-1.606020	0.298741	H	-0.506305	-1.569824	-0.127136
N	-0.838917	2.541645	-0.229218	N	0.275917	2.521360	0.160801	N	0.285324	2.559916	-0.263338
H	-0.933473	1.650984	0.250197	H	0.123325	1.602021	0.565998	H	0.119344	1.643820	0.145062
C	-1.003884	-3.718346	0.472045	C	-0.820839	-3.662586	0.358348	C	-0.862129	-3.619182	-0.078673
C	-1.003494	3.717955	0.471091	C	0.512470	3.609975	0.966931	C	0.524898	3.647698	0.533092
O	-0.931359	-4.819932	-0.080576	O	-1.047958	-4.687195	-0.281525	O	-1.111344	-4.640049	-0.710325
O	-0.923790	4.819390	-0.080862	O	0.698379	4.733603	0.504821	O	0.725186	4.768134	0.077229
C	-1.317703	-4.712863	2.687262	C	-0.778182	-4.676277	2.639961	C	-0.791268	-4.634529	2.193595
H	-1.912765	-4.426502	3.559600	C	-0.917433	-6.666800	4.619477	C	-0.913104	-6.615970	4.131667
H	-1.847947	-5.520413	2.174142	C	-1.143654	-5.984710	2.295511	C	-1.146302	-5.947719	1.835054
C	0.068233	-5.185037	3.123147	C	-0.485375	-4.374899	3.979750	C	-0.502502	-4.338443	3.541157
H	0.582444	-4.361638	3.637072	C	-0.556185	-5.360737	4.957182	C	-0.560968	-5.321025	4.510718
H	0.659180	-5.428007	2.230155	C	-1.207453	-6.963309	3.289470	C	-1.205577	-6.934352	2.809268
C	-0.004407	-6.402862	4.042544	H	-1.374574	-6.234258	1.269894	H	-1.372737	-6.192354	0.808155
H	-0.615964	-6.157381	4.921004	H	-0.202192	-3.360358	4.250411	H	-0.228960	-3.325074	3.820853
H	-0.520823	-7.218794	3.519960	H	-0.325782	-5.104152	5.987255	H	-0.336404	-5.090984	5.545570
C	1.377336	-6.873719	4.493075	H	-1.492086	-7.973483	3.007973	H	-1.478639	-7.948017	2.539796
H	1.898908	-6.080254	5.040161	H	-0.972755	-7.438147	5.381579	C	0.710513	4.227764	2.946938
H	1.994836	-7.146950	3.629990	C	0.720372	4.187815	3.387222	C	1.064034	5.820552	5.191230
H	1.308811	-7.747265	5.149131	C	1.097403	5.780008	5.672458	C	0.589692	3.681469	4.240736

C	-1.314143	4.713756	2.686175	C	0.603979	3.634396	4.672417	C	1.017521	5.592671	2.799211
H	-1.908013	4.428764	3.559756	C	1.029884	5.548041	3.251935	C	1.192546	6.383967	3.925662
H	-1.843410	5.522743	2.174273	C	1.213444	6.326554	4.396306	C	0.764469	4.469375	5.362013
C	0.073813	5.182576	3.119338	C	0.791566	4.423450	5.801008	H	0.354513	2.627364	4.357804
H	0.587095	4.357858	3.632057	H	0.364968	2.578855	4.780185	H	1.117373	6.028072	1.816322
H	0.663543	5.424363	2.225259	H	1.126093	5.990715	2.270944	H	1.428333	7.436487	3.818593
C	0.005772	6.400418	4.039010	H	1.452327	7.379818	4.276572	H	0.669006	4.046553	6.355242
H	-0.604664	6.156137	4.918596	H	0.696040	3.973136	6.784910	N	-0.707579	-3.576231	1.294015
H	-0.509757	7.217639	3.517556	H	1.243089	6.398021	6.553205	H	-0.472135	-2.678299	1.704479
C	1.389635	6.867827	4.486716	N	-0.691806	-3.611948	1.723065	N	0.515602	3.352050	1.883061
H	1.910187	6.073136	5.032954	H	-0.470651	-2.711221	2.133832	H	0.315396	2.392293	2.145019
H	1.324732	7.741768	5.142624	N	0.518527	3.310330	2.305399	N	1.244242	6.660097	6.363707
H	2.006027	7.139084	3.622220	H	0.327103	2.348875	2.565344	O	1.121711	6.148774	7.469132
N	-1.282569	-3.563773	1.793342					O	1.508839	7.843304	6.197701
H	-1.179720	-2.647163	2.211972					N	-0.975210	-7.657694	5.143227
N	-1.283484	3.564487	1.792251					O	-1.297133	-8.786686	4.797806
H	-1.188430	2.646879	2.210529					O	-0.702757	-7.362234	6.299444

Receptor **5** ΔG^0 = -2898.17201 au				Receptor **6** ΔG^0 = -2422.71753 au				Receptor **7** ΔG^0 = -2302.83125 au			
C	-0.420198	0.480790	-4.803589	C	-0.413275	0.427067	-4.179725	C	-0.420548	0.478890	-4.810129
N	-0.295492	0.217532	-1.929363	N	-0.262611	0.157004	-1.307990	N	-0.285943	0.214346	-1.937448
C	-0.607067	-0.734831	-4.143314	C	-0.569210	-0.794527	-3.522433	C	-0.610460	-0.736305	-4.149856
C	-0.164899	1.557678	-3.952834	C	-0.175699	1.506844	-3.327412	C	-0.157392	1.554666	-3.960296
C	-0.113967	1.384817	-2.549083	C	-0.110359	1.330103	-1.924637	C	-0.101236	1.381205	-2.556982
C	-0.536765	-0.819751	-2.732579	C	-0.487939	-0.882616	-2.112685	C	-0.535231	-0.821819	-2.739543
S	-0.935180	-2.295030	-4.850020	S	-0.869350	-2.359156	-4.232249	S	-0.946541	-2.295423	-4.855042
S	0.126759	3.220595	-4.390262	S	0.074041	3.177137	-3.760402	S	0.139563	3.216654	-4.397632
C	-0.980328	-3.046559	-3.268493	C	-0.888237	-3.116330	-2.652215	C	-0.987317	-3.047124	-3.272371
H	-1.169602	-4.106702	-3.199877	H	-1.055545	-4.180279	-2.585742	H	-1.180686	-4.106461	-3.203053
C	-0.756124	-2.172148	-2.253562	C	-0.676980	-2.240664	-1.635550	C	-0.756016	-2.173636	-2.258193
C	0.309371	3.673091	-2.708821	C	0.261954	3.627230	-2.078331	C	0.329650	3.667606	-2.715643
H	0.519732	4.703093	-2.466267	H	0.452196	4.660646	-1.833521	H	0.544921	4.696472	-2.473177
C	0.159456	2.628763	-1.853416	C	0.142719	2.576811	-1.225462	C	0.179049	2.623440	-1.859904
C	-0.426226	0.604358	-6.300004	C	-0.430513	0.552652	-5.675815	C	-0.430118	0.603956	-6.306438
H	-0.784611	1.600399	-6.588082	H	-0.806359	1.542880	-5.961441	H	-0.798693	1.596839	-6.592922
H	-1.127457	-0.123305	-6.727165	H	-1.122370	-0.186099	-6.099363	H	-1.124368	-0.130098	-6.733751
C	0.974188	0.373547	-6.893789	C	0.970039	0.343498	-6.277228	C	0.971908	0.387354	-6.901988
H	1.329334	-0.621250	-6.598395	H	1.341924	-0.645606	-5.983127	H	1.335773	-0.604954	-6.608748
H	1.671099	1.102677	-6.462940	H	1.657976	1.083261	-5.850181	H	1.663083	1.121407	-6.470437
C	0.961756	0.495115	-8.415101	C	0.947810	0.463646	-7.798465	C	0.957471	0.512030	-8.422965
H	1.959980	0.328479	-8.830987	H	1.946235	0.311888	-8.219434	H	1.956852	0.355033	-8.839761
H	0.627439	1.492091	-8.722781	H	0.596702	1.455072	-8.105342	H	0.614416	1.506787	-8.728165
H	0.281730	-0.240806	-8.858221	H	0.276774	-0.282945	-8.237386	H	0.283518	-0.228535	-8.867399
N	-0.703412	-2.383045	-0.878688	N	-0.614260	-2.452033	-0.262091	N	-0.698679	-2.381855	-0.884425
H	-0.520738	-1.549787	-0.325568	H	-0.447777	-1.615654	0.291306	H	-0.504443	-1.549340	-0.334752
N	0.226767	2.587406	-0.463673	N	0.224993	2.529700	0.162347	N	0.252126	2.577689	-0.471752
H	0.071800	1.669568	-0.055424	H	0.100380	1.605737	0.566477	H	0.086109	1.660975	-0.065940
C	-0.851404	-3.604709	-0.272602	C	-0.770298	-3.673116	0.349057	C	-0.842499	-3.605086	-0.271331
C	0.466719	3.676614	0.335356	C	0.465059	3.617812	0.967264	C	0.481927	3.667637	0.336289
O	-1.080352	-4.629235	-0.908900	O	-0.993554	-4.698908	-0.290382	O	-1.079918	-4.628068	-0.910720
O	0.660668	4.797650	-0.125591	O	0.631389	4.745140	0.506672	O	0.673855	4.790448	-0.126394
C	-0.801223	-4.620380	2.002748	C	-0.764162	-4.686455	2.628493	C	-0.788280	-4.621234	2.009039
C	-0.939559	-6.613572	3.959725	C	-0.968158	-6.648481	4.574911	C	-0.927533	-6.626928	4.004219
C	-1.162740	-5.928951	1.648878	C	-1.160250	-5.984343	2.278562	C	-1.139334	-5.934003	1.668092
C	-0.512248	-4.325895	3.346927	C	-0.471389	-4.396001	3.970176	C	-0.512984	-4.321345	3.350528
C	-0.579465	-5.312630	4.317201	C	-0.573583	-5.374473	4.951931	C	-0.584763	-5.309337	4.326367
C	-1.226137	-6.913950	2.630614	C	-1.260319	-6.969605	3.262053	C	-1.203432	-6.909132	2.663953
H	-1.393641	-6.174508	0.622888	H	-1.390497	-6.227894	1.251561	H	-1.362246	-6.191114	0.642365
H	-0.235317	-3.313114	3.627128	H	-0.163227	-3.391544	4.248839	H	-0.244398	-3.304773	3.628877
H	-0.353674	-5.066285	5.350383	H	-0.350859	-5.153972	5.990918	H	-0.369044	-5.047177	5.359707
H	-1.508713	-7.923223	2.346461	H	-1.566939	-7.978782	3.005252	H	-1.480965	-7.922190	2.379602
C	0.675225	4.261447	2.748904	C	0.715861	4.197007	3.380542	C	0.674590	4.250774	2.756460
C	1.065150	5.862202	5.008912	C	1.132688	5.774351	5.620955	C	1.054812	5.855955	5.059100
C	0.573193	3.713710	4.041176	C	0.609763	3.651467	4.669545	C	0.560553	3.702839	4.042507
C	0.977043	5.622634	2.602890	C	1.036259	5.553578	3.234788	C	0.977368	5.611870	2.626688
C	1.170307	6.411162	3.735385	C	1.243966	6.344092	4.365993	C	1.160042	6.387974	3.772331
C	0.767115	4.504989	5.159918	C	0.818809	4.435851	5.797201	C	0.747499	4.494830	5.168943
H	0.338402	2.659307	4.160292	H	0.361911	2.600000	4.790103	H	0.320010	2.648145	4.156475
H	1.060296	6.062610	1.620133	H	1.124614	5.992428	2.251525	H	1.066277	6.061490	1.647992

H	1.403168	7.463741	3.609002	H	1.492688	7.396172	4.267720	H	1.390685	7.444356	3.650361
H	0.683650	4.066354	6.150517	H	0.738726	4.018139	6.795687	H	0.649094	4.043319	6.153677
N	-0.716498	-3.557065	1.096878	N	-0.651632	-3.622114	1.714206	N	-0.699165	-3.556536	1.090335
H	-0.490991	-2.657723	1.508927	H	-0.429456	-2.723058	2.127642	H	-0.479940	-2.655548	1.501339
N	0.465562	3.382270	1.680343	N	0.497556	3.315952	2.304451	N	0.471172	3.371692	1.673843
H	0.272166	2.422140	1.944734	H	0.317719	2.353373	2.568293	H	0.279400	2.410595	1.934615
C	-0.960818	-7.690954	4.996271	F	-1.075615	-7.609780	5.527483	C	-0.976270	-7.706711	5.055853
C	1.297074	6.690295	6.232043	F	1.339671	6.547898	6.716658	H	-0.061364	-8.310376	5.040593
F	-1.327125	-7.231961	6.209491					H	-1.818607	-8.384643	4.887279
F	0.254030	-8.265975	5.168513					H	-1.074236	-7.276850	6.056698
F	-1.805241	-8.692741	4.684587					C	1.290015	6.706701	6.281722
F	2.449337	6.363662	6.863165					H	2.350687	6.708820	6.558932
F	0.319970	6.528492	7.151154					H	0.725822	6.329141	7.139513
F	1.368723	8.006808	5.967847					H	0.994402	7.744665	6.102861

Anion complex **2**@Cl$^-$ ΔG^0 = -2537.01404 au				Anion complex **3**@Cl$^-$ ΔG^0 = -2684.65076 au				Anion complex **4**@Cl$^-$ ΔG^0 = -3093.52609 au			
C	-0.205518	0.004661	-4.465160	C	-0.404673	0.011450	-4.318619	C	-0.367869	0.007425	-4.764501
N	-0.659027	-0.018595	-1.618240	N	-0.263329	0.007144	-1.437639	N	-0.246885	0.006166	-1.881835
C	-0.339221	-1.174769	-3.727467	C	-0.373248	-1.163340	-3.561158	C	-0.335507	-1.166654	-4.005303
C	-0.308298	1.171803	-3.703088	C	-0.359973	1.183985	-3.558378	C	-0.336173	1.180852	-4.004398
C	-0.530683	1.111197	-2.308710	C	-0.291358	1.131972	-2.147587	C	-0.276887	1.129269	-2.593594
C	-0.560478	-1.137027	-2.331826	C	-0.303224	-1.115407	-2.150571	C	-0.276036	-1.116408	-2.594389
S	-0.268930	-2.824136	-4.293552	S	-0.405835	-2.818919	-4.117210	S	-0.359540	-2.824044	-4.556940
S	-0.197480	2.830073	-4.236043	S	-0.374818	2.840995	-4.109810	S	-0.360722	2.838874	-4.554396
C	-0.530875	-3.439362	-2.667334	C	-0.327937	-3.420953	-2.468295	C	-0.292234	-3.422512	-2.907648
H	-0.563576	-4.507023	-2.512342	H	-0.323459	-4.487528	-2.301585	H	-0.287136	-4.488964	-2.738914
C	-0.669275	-2.456182	-1.739852	C	-0.280027	-2.429877	-1.541098	C	-0.253242	-2.429052	-1.983449
C	-0.445357	3.418564	-2.597866	C	-0.292317	3.438134	-2.459644	C	-0.293857	3.435724	-2.904323
H	-0.453959	4.483455	-2.421896	H	-0.277599	4.504234	-2.290369	H	-0.288653	4.502007	-2.734305
C	-0.606326	2.420548	-1.690135	C	-0.255020	2.444531	-1.534713	C	-0.254543	2.441222	-1.981310
C	0.092256	0.017658	-5.936870	C	-0.413907	0.010608	-5.820156	C	-0.369784	0.007413	-6.266058
H	-0.355338	0.908241	-6.395678	H	-0.929139	0.906195	-6.188649	H	-0.899786	0.893996	-6.635415
H	-0.368882	-0.855794	-6.414698	H	-0.978066	-0.856627	-6.185284	H	-0.917454	-0.868923	-6.634278
C	1.606254	0.009410	-6.208221	C	1.013655	-0.030925	-6.392317	C	1.059607	-0.007161	-6.834640
H	2.046923	-0.886099	-5.753304	H	1.522137	-0.928589	-6.020021	H	1.586503	-0.892524	-6.458651
H	2.063558	0.876023	-5.715045	H	1.574776	0.833503	-6.016831	H	1.602464	0.869954	-6.461968
C	1.905934	0.039185	-7.704239	C	1.004301	-0.028866	-7.918565	C	1.052839	-0.010888	-8.360855
H	2.984217	0.026210	-7.890117	H	2.022297	-0.063142	-8.318443	H	2.072086	-0.023906	-8.758703
H	1.492616	0.943207	-8.165061	H	0.518243	0.874776	-8.302803	H	0.547353	0.880119	-8.749437
H	1.463882	-0.828419	-8.206548	H	0.457877	-0.896567	-8.304704	H	0.526797	-0.892144	-8.744299
N	-0.889240	-2.508499	-0.370791	N	-0.213416	-2.467744	-0.154618	N	-0.196121	-2.463760	-0.594687
H	-0.952259	-1.604553	0.104805	H	-0.186217	-1.555027	0.308115	H	-0.167690	-1.549702	-0.132853
N	-0.820369	2.450375	-0.319538	N	-0.189850	2.479391	-0.148107	N	-0.196109	2.473296	-0.592581
H	-0.893014	1.538697	0.139163	H	-0.171801	1.566025	0.313927	H	-0.168098	1.558013	-0.133521
C	-1.017263	-3.661643	0.372123	C	-0.175269	-3.612310	0.603834	C	-0.178987	-3.603032	0.161018
C	-0.950522	3.591332	0.442247	C	-0.155419	3.622543	0.612746	C	-0.182296	3.610359	0.166455
O	-0.909726	-4.783375	-0.136169	O	-0.206099	-4.735773	0.101579	O	-0.217616	-4.729139	-0.326493
O	-0.860947	4.721928	-0.050266	O	-0.180528	4.747156	0.112690	O	-0.224781	4.737769	-0.317736
C	-1.259111	-4.534535	2.649322	C	-0.035330	-4.268561	3.007701	C	-0.074926	-4.239677	2.563493
H	-1.904463	-4.251103	3.486805	C	0.097459	-5.942591	5.262619	C	0.010401	-5.892633	4.791052
H	-1.697561	-5.419090	2.177983	C	-0.125618	-5.659370	2.856650	C	-0.162368	-5.635838	2.407234
C	0.148852	-4.847814	3.153437	C	0.120869	-3.726300	4.294315	C	0.053380	-3.694095	3.857960
H	0.568858	-3.946925	3.621472	C	0.185441	-4.555985	5.407568	C	0.096544	-4.511651	4.970416
H	0.789246	-5.092858	2.295434	C	-0.057474	-6.478487	3.985624	C	-0.119096	-6.456762	3.524975
C	0.157043	-6.002839	4.152695	H	-0.248107	-6.092622	1.873937	H	-0.263577	-6.069199	1.423140
H	-0.514085	-5.762500	4.988103	H	0.190657	-2.647271	4.412444	H	0.120067	-2.616336	3.978150
H	-0.253455	-6.899741	3.670280	H	0.306199	-4.114312	6.392723	H	0.196932	-4.089793	5.963607
C	1.556921	-6.300350	4.686685	H	-0.129278	-7.554770	3.853840	H	-0.186661	-7.532673	3.412674
H	1.969161	-5.425825	5.202724	H	0.147704	-6.592012	6.131316	C	-0.074277	4.238483	2.571104
H	2.238357	-6.557454	3.867768	C	-0.040836	4.272409	3.020003	C	0.024109	5.884092	4.803936
H	1.547410	-7.136291	5.393325	C	0.057844	5.937847	5.283315	C	0.065552	3.688786	3.862797
C	-1.215709	4.438844	2.725893	C	0.115395	3.726468	4.305031	C	-0.168929	5.634827	2.420703
H	-1.801170	4.097494	3.585139	C	-0.148274	5.662719	2.874729	C	-0.118374	6.452116	3.540954
H	-1.743005	5.289481	2.282885	C	-0.096747	6.477486	4.007813	C	0.114677	4.502693	4.977757
C	0.180763	4.865216	3.176190	C	0.162846	4.551733	5.422454	H	0.137309	2.610837	3.978649
H	0.694238	4.000737	3.618528	H	0.198150	2.647877	4.418584	H	-0.279862	6.070983	1.438863

H	0.761553	5.169506	2.295239	H	-0.271222	6.098966	1.893368	H	-0.189683	7.528292	3.432966
C	0.131196	6.010693	4.184997	H	-0.181622	7.553265	3.880307	H	0.224834	4.078216	5.968824
H	-0.464143	5.701022	5.054352	H	0.283869	4.107002	6.406164	N	-0.112650	-3.333095	1.514450
H	-0.393028	6.864554	3.735565	H	0.095027	6.583722	6.155242	H	-0.070878	-2.344904	1.778022
C	1.522557	6.444198	4.642946	N	-0.100506	-3.350616	1.947467	N	-0.112987	3.335778	1.518931
H	2.053034	5.610035	5.115968	H	-0.067349	-2.365147	2.215453	H	-0.066229	2.346182	1.777444
H	1.468721	7.263923	5.366382	N	-0.090021	3.357894	1.956105	N	0.082584	6.749506	5.968128
H	2.123502	6.783255	3.791604	H	-0.056817	2.371490	2.220998	O	0.202291	6.233225	7.072164
N	-1.294771	-3.447571	1.684346	Cl	-0.093020	0.008119	2.228510	O	0.012827	7.960297	5.799793
H	-1.246813	-2.490909	2.032892					N	0.059039	-6.762055	5.952536
N	-1.204550	3.357146	1.754546					O	-0.013891	-7.971980	5.779453
H	-1.164156	2.396349	2.091274					O	0.173339	-6.249920	7.059050
Cl	-1.054472	-0.053734	2.036936					Cl	-0.059136	0.002649	1.770782

Anion complex **5**@Cl^- ΔG^0 = -3358.53642 au				Anion complex **6**@Cl^- ΔG^0 = -2883.08076 au				Anion complex **7**@Cl^- ΔG^0 = -2763.19463 au			
C	-0.555740	0.143647	-4.948201	C	-0.380311	0.019405	-4.324972	C	-0.391445	0.003847	-4.964834
N	-0.359662	0.062704	-2.071415	N	-0.224127	0.012436	-1.445004	N	-0.241672	0.007555	-2.084732
C	-0.532099	-1.050899	-4.221969	C	-0.334449	-1.156223	-3.569192	C	-0.366800	-1.168793	-4.203725
C	-0.475582	1.295006	-4.158740	C	-0.341223	1.191363	-3.563542	C	-0.334531	1.178594	-4.208589
C	-0.380352	1.205315	-2.752043	C	-0.265196	1.137565	-2.153395	C	-0.262079	1.130127	-2.798385
C	-0.434801	-1.039843	-2.811871	C	-0.257986	-1.109581	-2.158867	C	-0.292981	-1.116948	-2.793444
S	-0.610319	-2.690996	-4.816685	S	-0.359180	-2.811072	-4.126510	S	-0.413954	-2.826255	-4.753070
S	-0.469535	2.966298	-4.666943	S	-0.374737	2.849571	-4.111572	S	-0.339909	2.834602	-4.764016
C	-0.511670	-3.335488	-3.185791	C	-0.269278	-3.414504	-2.478790	C	-0.338869	-3.422715	-3.101762
H	-0.525989	-4.405911	-3.046035	H	-0.257635	-4.481311	-2.313734	H	-0.344609	-4.488721	-2.931576
C	-0.426261	-2.369235	-2.236060	C	-0.222980	-2.424453	-1.550635	C	-0.281367	-2.428967	-2.178096
C	-0.342569	3.518672	-3.004065	C	-0.290884	3.444093	-2.460190	C	-0.248216	3.435319	-3.114917
H	-0.302136	4.579686	-2.808529	H	-0.286564	4.510014	-2.288973	H	-0.225922	4.501747	-2.948307
C	-0.304932	2.500223	-2.106999	C	-0.239452	2.448967	-1.537750	C	-0.214298	2.443519	-2.188154
C	-0.596423	0.189368	-6.448569	C	-0.401556	0.020818	-5.826369	C	-0.411741	0.001777	-6.466218
H	-1.134391	1.088429	-6.774349	H	-0.922583	0.915677	-6.188375	H	-0.950647	0.886045	-6.828626
H	-1.155350	-0.673755	-6.830540	H	-0.966919	-0.847168	-6.187948	H	-0.960068	-0.877239	-6.826827
C	0.817349	0.193198	-7.054244	C	1.020445	-0.015542	-6.411711	C	1.009689	-0.007235	-7.053457
H	1.350057	-0.708350	-6.728313	H	1.536095	-0.911243	-6.044532	H	1.542654	-0.893259	-6.687521
H	1.373853	1.052730	-6.661228	H	1.581982	0.850895	-6.041630	H	1.556067	0.869422	-6.685037
C	0.770289	0.252741	-8.578611	C	0.996418	-0.013301	-7.937786	C	0.982129	-0.004534	-8.579527
H	1.778393	0.254730	-9.004193	H	2.010544	-0.045096	-8.347537	H	1.995609	-0.014550	-8.991968
H	0.257002	1.160188	-8.915629	H	0.504331	0.889069	-8.317232	H	0.470127	0.887615	-8.956842
H	0.231410	-0.610434	-8.984759	H	0.448494	-0.882362	-8.318611	H	0.451355	-0.884347	-8.959800
N	-0.333618	-2.443207	-0.851587	N	-0.149389	-2.463346	-0.164280	N	-0.217002	-2.460374	-0.791264
H	-0.274368	-1.543105	-0.366811	H	-0.119550	-1.551404	0.300295	H	-0.184011	-1.545067	-0.334135
N	-0.206132	2.495096	-0.720783	N	-0.168687	2.478798	-0.151045	N	-0.143473	2.476965	-0.802163
H	-0.198266	1.568448	-0.284859	H	-0.133994	1.563513	0.306446	H	-0.139976	1.562350	-0.342689
C	-0.328893	-3.603786	-0.122601	C	-0.128564	-3.607954	0.593945	C	-0.180540	-3.602097	-0.026838
C	-0.089393	3.612200	0.064527	C	-0.165652	3.617381	0.616335	C	-0.122368	3.617762	-0.035432
O	-0.406400	-4.714674	-0.642831	O	-0.170723	-4.731715	0.093110	O	-0.203945	-4.727729	-0.525737
O	-0.078396	4.751045	-0.397883	O	-0.215981	4.744635	0.124282	O	-0.146631	4.744468	-0.531435
C	-0.189270	-4.310304	2.262746	C	-0.017806	-4.270165	2.995590	C	-0.044731	-4.254541	2.376338
C	-0.089328	-6.030999	4.465829	C	0.059460	-5.928458	5.215582	C	0.084488	-5.938620	4.650627
C	-0.314952	-5.696898	2.078149	C	-0.135526	-5.658384	2.840100	C	-0.141933	-5.643928	2.232995
C	-0.017220	-3.802387	3.562307	C	0.139417	-3.734829	4.284443	C	0.112731	-3.716130	3.663063
C	0.030718	-4.653450	4.655289	C	0.177798	-4.559964	5.401923	C	0.173329	-4.546471	4.775273
C	-0.264354	-6.543123	3.181260	C	-0.095052	-6.489620	3.960978	C	-0.076509	-6.459217	3.365036
H	-0.447801	-6.105576	1.086778	H	-0.258109	-6.088707	1.856364	H	-0.271536	-6.083202	1.253564
H	0.082279	-2.729764	3.707204	H	0.231111	-2.658823	4.409631	H	0.183204	-2.637381	3.785250
H	0.166861	-4.242256	5.650673	H	0.297592	-4.150570	6.399890	H	0.291766	-4.101626	5.760972
H	-0.360816	-7.614655	3.029597	H	-0.185426	-7.566190	3.855544	H	-0.157996	-7.536233	3.233323
C	0.176496	4.184407	2.473568	C	-0.078825	4.260238	3.024179	C	-0.065795	4.256380	2.375088
C	0.518547	5.776483	4.746955	C	-0.031098	5.901375	5.257167	C	-0.045258	5.929053	4.660727
C	0.324828	3.596749	3.744667	C	0.071281	3.715707	4.309985	C	-0.192097	3.715194	3.664135
C	0.197973	5.581549	2.356485	C	-0.204877	5.648726	2.878336	C	0.071383	5.642384	2.234486
C	0.371386	6.365842	3.495250	C	-0.179346	6.471423	4.005875	C	0.076811	6.452673	3.372320
C	0.494700	4.383926	4.869587	C	0.095070	4.532385	5.434079	C	-0.181090	4.540062	4.781788
H	0.305399	2.513768	3.837061	H	0.169138	2.639430	4.427720	H	-0.303841	2.639540	3.782230
H	0.080677	6.049749	1.389842	H	-0.322462	6.085795	1.896929	H	0.169580	6.083766	1.252124

H	0.388004	7.446404	3.393306	H	-0.276138	7.548183	3.908254	H	0.180280	7.527970	3.242526
H	0.609773	3.915209	5.843136	H	0.209971	4.116494	6.429952	H	-0.285039	4.094122	5.768709
N	-0.227065	-3.372314	1.230168	N	-0.056497	-3.347975	1.937547	N	-0.107117	-3.336435	1.313818
H	-0.159170	-2.392791	1.517684	H	-0.013249	-2.363319	2.208394	H	-0.091668	-2.349990	1.581241
N	0.012216	3.305510	1.402397	N	-0.101302	3.346750	1.958255	N	-0.077767	3.345231	1.305329
H	0.001263	2.312144	1.643991	H	-0.050044	2.360508	2.221531	H	-0.086884	2.355703	1.561821
C	-0.080738	-6.962328	5.635165	F	0.094672	-6.743168	6.302420	C	0.184808	-6.838143	5.857134
C	0.738457	6.602243	5.973061	F	-0.009327	6.707738	6.350474	H	1.231709	-7.062022	6.093577
F	0.495462	-6.421045	6.724902	Cl	0.018864	-0.002506	2.214628	H	-0.325401	-7.789914	5.682625
F	0.577102	-8.110958	5.373555					H	-0.257199	-6.365672	6.739545
F	-1.328199	-7.332152	6.013345					C	-0.008083	6.816953	5.879565
F	0.539606	7.915722	5.764538					H	0.993122	6.829179	6.326146
F	-0.077257	6.239597	6.987448					H	-0.704407	6.464109	6.646445
F	1.997695	6.478529	6.456432					H	-0.269467	7.847666	5.623512
Cl	-0.098347	-0.046870	1.586231					Cl	-0.109971	0.006655	1.617788

Anion complex **2**@$MeCO_2^-$ ΔG^0 = -2305.15974 au				Anion complex **3**@$MeCO_2^-$ ΔG^0 = -2452.80352 au				Anion complex **4**@$MeCO_2^-$ ΔG^0 = -2861.67536 au			
C	-0.339870	-0.120307	-4.617647	C	-0.373303	0.016088	-4.479691	C	-4.833354	0.010629	0.294817
N	-0.119962	-0.528868	-1.768956	N	-0.240906	0.011714	-1.592334	N	-1.943946	0.007946	0.189861
C	0.884545	-0.265417	-3.964945	C	-0.349736	-1.153121	-3.723895	C	-4.076396	-1.158078	0.273291
C	-1.448413	-0.189966	-3.773572	C	-0.326133	1.182777	-3.721879	C	-4.074917	1.177698	0.259064
C	-1.298434	-0.378918	-2.377883	C	-0.262072	1.145641	-2.304531	C	-2.657268	1.140946	0.208587
C	0.949907	-0.477704	-2.565844	C	-0.282282	-1.120526	-2.306689	C	-2.658809	-1.123931	0.220521
S	2.476377	-0.227014	-4.673139	S	-0.383325	-2.779421	-4.338118	S	-4.687923	-2.786045	0.302882
S	-3.129926	-0.072380	-4.218498	S	-0.326973	2.810970	-4.332413	S	-4.684309	2.806796	0.266395
C	3.217105	-0.514308	-3.110709	C	-0.306875	-3.422309	-2.718024	C	-3.067377	-3.426623	0.243972
H	4.291823	-0.581198	-3.048471	H	-0.301405	-4.491817	-2.586161	H	-2.934031	-4.496098	0.242393
C	2.318462	-0.635568	-2.096723	C	-0.260473	-2.469428	-1.746899	C	-2.098626	-2.471544	0.205757
C	-3.621506	-0.256026	-2.546500	C	-0.244469	3.448598	-2.710275	C	-3.062606	3.444314	0.203924
H	-4.673428	-0.241969	-2.308297	H	-0.220904	4.517537	-2.575428	H	-2.927589	4.513519	0.190120
C	-2.578314	-0.399334	-1.685469	C	-0.219282	2.492608	-1.741586	C	-2.095202	2.487447	0.180745
C	-0.451968	0.152041	-6.090325	C	-0.375670	0.016469	-5.981347	C	-6.334951	0.011126	0.289568
H	-1.391550	-0.265721	-6.472867	H	-0.873324	0.921433	-6.351037	H	-6.705882	0.909456	0.798299
H	0.363139	-0.354016	-6.622754	H	-0.954756	-0.839241	-6.350583	H	-6.706567	-0.851925	0.856001
C	-0.403150	1.658983	-6.395136	C	1.053322	-0.049975	-6.547557	C	-6.897850	-0.034840	-1.141384
H	0.538087	2.072128	-6.012826	H	1.545671	-0.954415	-6.170037	H	-6.521605	-0.932688	-1.646481
H	-1.214930	2.160361	-5.854129	H	1.627099	0.806483	-6.172933	H	-6.520920	0.829019	-1.702305
C	-0.524455	1.931086	-7.891904	C	1.050558	-0.053314	-8.073546	C	-8.424023	-0.034951	-1.140843
H	-0.484653	3.004449	-8.101149	H	2.069774	-0.101572	-8.468830	H	-8.818102	-0.071134	-2.161002
H	-1.472189	1.543955	-8.282031	H	0.577447	0.854874	-8.463262	H	-8.812422	0.868667	-0.658115
H	0.289908	1.447181	-8.442359	H	0.495228	-0.915616	-8.459017	H	-8.811921	-0.902490	-0.595528
N	2.529889	-0.893688	-0.749688	N	-0.197174	-2.649301	-0.368786	N	-0.717808	-2.648537	0.157557
H	1.702549	-1.061813	-0.165171	H	-0.198980	-1.812990	0.226046	H	-0.126729	-1.808887	0.129255
N	-2.580039	-0.542848	-0.304846	N	-0.159834	2.665703	-0.362630	N	-0.713911	2.660910	0.137645
H	-1.675579	-0.472667	0.177884	H	-0.184592	1.826214	0.227259	H	-0.125058	1.819260	0.126473
C	3.774609	-1.062339	-0.174246	C	-0.139225	-3.887920	0.237156	C	-0.111786	-3.880830	0.142279
C	-3.718654	-0.638748	0.469000	C	-0.114463	3.901140	0.250501	C	-0.104203	3.891333	0.124175
O	4.823312	-0.876652	-0.807043	O	-0.132815	-4.939296	-0.405897	O	-0.739030	-4.937972	0.165303
O	-4.851519	-0.694136	-0.027312	O	-0.088720	4.955242	-0.387735	O	-0.728751	4.950330	0.131903
C	4.957269	-1.561825	1.912337	C	-0.024597	-4.888494	2.512850	C	2.166799	-4.859305	0.081012
H	4.750709	-2.258832	2.730620	C	0.103322	-6.870319	4.508926	C	4.142329	-6.815886	0.042677
H	5.741156	-2.005360	1.289603	C	0.018353	-6.245074	2.158464	C	1.811756	-6.222855	0.075036
C	5.433553	-0.222418	2.473633	C	-0.003566	-4.538833	3.874773	C	3.534242	-4.505367	0.065304
H	4.637250	0.207872	3.096227	C	0.060388	-5.518069	4.858406	C	4.519793	-5.471686	0.046412
H	5.602656	0.472963	1.640664	C	0.081735	-7.216774	3.159674	C	2.802395	-7.193753	0.056048
C	6.712636	-0.365644	3.295601	H	0.003432	-6.536011	1.117887	H	0.772621	-6.515664	0.085031
H	6.536269	-1.071891	4.117995	H	-0.037048	-3.487572	4.152427	H	3.810324	-3.454582	0.068910
H	7.497718	-0.807626	2.667636	H	0.076029	-5.220861	5.903264	H	5.567101	-5.193544	0.035400
C	7.196220	0.969448	3.858889	H	0.114623	-8.263145	2.868073	H	2.532999	-8.243622	0.051544
H	6.434785	1.416653	4.508061	H	0.153772	-7.637297	5.275862	C	2.178268	4.862056	0.083221
H	7.405035	1.679375	3.050509	C	-0.040068	4.890415	2.532637	C	4.160254	6.812062	0.047208
H	8.111799	0.848535	4.446629	C	0.068974	6.863550	4.538419	C	3.544596	4.503520	0.074133
C	-4.575196	-0.658026	2.760741	C	-0.024887	4.534520	3.892999	C	1.827689	6.226745	0.072577
H	-4.209813	-1.093137	3.696490	C	-0.001009	6.248919	2.184770	C	2.821526	7.194358	0.054613
H	-5.383678	-1.297537	2.393119	C	0.053470	7.216180	3.190668	C	4.533320	5.466627	0.056337
C	-5.098604	0.756334	3.009782	C	0.029360	5.509522	4.881407	H	3.817218	3.451784	0.081628
H	-4.277338	1.380665	3.387462	H	-0.055136	3.481830	4.165547	H	0.789452	6.522831	0.078457

H	-5.419854	1.189790	2.053488	H	-0.012205	6.544293	1.145416	H	2.555620	8.245102	0.046619
C	-6.260188	0.774687	4.001281	H	0.083613	8.264033	2.904191	H	5.579747	5.185065	0.049956
H	-5.937705	0.316488	4.945749	H	0.040421	5.207635	5.924996	N	1.266290	-3.807658	0.099686
H	-7.075391	0.149679	3.613203	H	0.112175	7.627056	5.309270	H	1.670250	-2.862239	0.093534
C	-6.777149	2.187650	4.265690	N	-0.086347	-3.822718	1.603948	N	1.274117	3.813653	0.102342
H	-5.985601	2.819185	4.684752	H	-0.108552	-2.883828	2.015276	H	1.674801	2.866711	0.106708
H	-7.614369	2.183278	4.971062	N	-0.095673	3.829279	1.617924	N	5.190559	7.832544	0.030153
H	-7.121173	2.655230	3.336069	H	-0.130168	2.887886	2.023467	O	6.363028	7.477119	0.024314
N	3.743633	-1.455828	1.121441	C	-0.213117	0.004910	2.594576	O	4.848802	9.008953	0.022244
H	2.833620	-1.574227	1.568548	O	-0.227280	1.112089	1.993638	N	5.169137	-7.839889	0.024879
N	-3.481760	-0.710661	1.804566	O	-0.193097	-1.108889	2.006432	O	4.823349	-9.015137	0.022012
H	-2.535609	-0.498566	2.124315	C	-0.183972	0.016278	4.115205	O	6.342772	-7.488456	0.013937
C	0.194794	-0.920645	2.237997	H	-0.604749	-0.905492	4.521921	C	2.231633	0.003109	0.123287
O	-0.676131	-0.122890	1.789949	H	-0.720168	0.883083	4.507542	O	1.634385	1.112478	0.109617
O	0.933645	-1.648547	1.519191	H	0.860562	0.088669	4.439304	O	1.637583	-1.106901	0.078813
C	0.358522	-0.989061	3.751001					C	3.751044	0.008433	0.159761
H	1.065079	-1.767086	4.045112					H	4.136030	-0.917931	0.590393
H	-0.613523	-1.176194	4.218038					H	4.121664	0.869507	0.720008
H	0.715556	-0.020413	4.116110					H	4.120376	0.088991	-0.868939

Anion complex **5**@$MeCO_2^-$ ΔG^0 = -3126.68875 au				Anion complex **6**@$MeCO_2^-$ ΔG^0 = -2651.23024 au				Anion complex **7**@$MeCO_2^-$ ΔG^0 = -2531.34619 au			
C	0.304191	5.047339	-0.513036	C	-0.337808	0.007427	-4.489798	C	-5.072786	0.007519	0.337291
N	0.212561	2.172507	-0.250424	N	-0.250747	0.005018	-1.600732	N	-2.186549	0.005166	0.228703
C	0.532675	4.387859	0.694677	C	-0.325199	-1.161315	-3.733027	C	-4.314459	-1.162657	0.336346
C	0.023936	4.196236	-1.581780	C	-0.304690	1.174674	-3.731704	C	-4.314152	1.176556	0.278251
C	0.001131	2.789840	-1.415496	C	-0.263539	1.138774	-2.313661	C	-2.898916	1.135020	0.237325
C	0.465811	2.976049	0.785751	C	-0.279113	-1.127512	-2.315134	C	-2.899985	-1.122757	0.270496
S	0.910305	5.105963	2.236659	S	-0.352437	-2.788897	-4.345444	S	-4.900678	-2.802902	0.407087
S	-0.335326	4.645838	-3.227679	S	-0.299875	2.802848	-4.342736	S	-4.902440	2.816104	0.242794
C	0.960394	3.530775	2.998423	C	-0.298933	-3.429790	-2.723544	C	-3.266744	-3.426426	0.332051
H	1.172079	3.470511	4.054408	H	-0.296246	-4.499111	-2.590136	H	-3.116221	-4.494286	0.344367
C	0.704327	2.506041	2.141569	C	-0.264469	-2.475532	-1.753516	C	-2.317080	-2.455847	0.258745
C	-0.493107	2.965489	-3.689593	C	-0.245005	3.441337	-2.720066	C	-3.267656	3.438005	0.188553
H	-0.728911	2.728360	-4.715063	H	-0.225725	4.510345	-2.585512	H	-3.119157	4.505679	0.154786
C	-0.284909	2.098517	-2.662996	C	-0.233781	2.485866	-1.750608	C	-2.315197	2.467363	0.200461
C	0.294706	6.543941	-0.636203	C	-0.318094	0.008046	-5.991297	C	-6.574241	0.009386	0.337420
H	0.624441	6.834260	-1.641583	H	-0.825892	0.904789	-6.367327	H	-6.940661	0.900034	0.863194
H	1.011539	6.977222	0.072424	H	-0.877288	-0.856995	-6.369036	H	-6.944354	-0.862662	0.890559
C	-1.103323	7.126767	-0.367782	C	1.118925	-0.033451	-6.539532	C	-7.145986	-0.012608	-1.089976
H	-1.427808	6.829742	0.637042	H	1.622134	-0.930153	-6.158000	H	-6.780046	-0.906714	-1.609116
H	-1.817026	6.688990	-1.076227	H	1.673600	0.831620	-6.156372	H	-6.766926	0.855447	-1.642617
C	-1.108163	8.647921	-0.491525	C	1.135150	-0.033039	-8.065544	C	-8.672280	-0.000259	-1.078686
H	-2.104696	9.055182	-0.295721	H	2.159738	-0.066826	-8.448239	H	-9.074204	-0.023816	-2.096161
H	-0.806639	8.957267	-1.498399	H	0.654870	0.869604	-8.459168	H	-9.049225	0.902100	-0.584761
H	-0.410560	9.098267	0.223201	H	0.596158	-0.901635	-8.459780	H	-9.063553	-0.869271	-0.538278
N	0.625728	1.140660	2.391885	N	-0.216602	-2.651126	-0.374056	N	-0.935941	-2.572099	0.165942
H	0.316301	0.539215	1.618617	H	-0.206270	-1.810872	0.214935	H	-0.402881	-1.707531	0.013070
N	-0.291168	0.707595	-2.664513	N	-0.199540	2.659871	-0.370551	N	-0.930573	2.587415	0.195728
H	0.039230	0.234481	-1.813451	H	-0.237674	1.820836	0.219363	H	-0.381186	1.732917	0.352572
C	0.819617	0.570782	3.626763	C	-0.167005	-3.886542	0.237975	C	-0.256124	-3.769860	0.185119
C	-0.596792	-0.057885	-3.762577	C	-0.147059	3.894290	0.243148	C	-0.261844	3.787019	0.097604
O	1.169757	1.219044	4.612429	O	-0.167490	-4.941755	-0.399381	O	-0.826393	-4.850506	0.349019
O	-0.993616	0.422632	-4.823632	O	-0.095096	4.948906	-0.393185	O	-0.846316	4.857869	-0.080337
C	0.667777	-1.659802	4.710236	C	-0.048103	-4.881786	2.513805	C	2.069778	-4.629490	-0.029276
C	0.781481	-3.578338	6.746750	C	0.110425	-6.834777	4.480424	C	4.194826	-6.503300	-0.135843
C	1.177130	-1.320142	5.972849	C	0.006022	-6.237789	2.159065	C	1.851178	-5.969167	0.316627
C	0.218698	-2.976447	4.484430	C	-0.025829	-4.532419	3.875390	C	3.360077	-4.236943	-0.421064
C	0.275984	-3.925259	5.489660	C	0.054465	-5.504017	4.865311	C	4.398561	-5.158074	-0.468957
C	1.229309	-2.281981	6.979658	C	0.086101	-7.216367	3.151892	C	2.909058	-6.879046	0.257202
H	1.528558	-0.316918	6.165956	H	-0.011190	-6.530714	1.119385	H	0.870123	-6.297150	0.631355
H	-0.178407	-3.243624	3.508420	H	-0.069283	-3.483369	4.157543	H	3.542009	-3.197290	-0.685514
H	-0.075566	-4.935664	5.298139	H	0.074374	-5.236043	5.916857	H	5.388424	-4.823747	-0.773057
H	1.626098	-2.006624	7.951819	H	0.131048	-8.268742	2.889171	H	2.718759	-7.914443	0.532696
C	-0.618416	-2.453221	-4.433274	C	-0.074099	4.886521	2.522161	C	2.064196	4.670278	0.143398
C	-0.954341	-4.698374	-6.071978	C	0.097902	6.834170	4.492904	C	4.175100	6.558160	0.005446
C	-0.226934	-3.731551	-3.991184	C	-0.062000	4.534501	3.883233	C	3.373870	4.317702	0.508464
C	-1.188737	-2.316349	-5.709059	C	-0.004579	6.242514	2.170128	C	1.817415	5.978326	-0.291377
C	-1.351941	-3.439597	-6.515311	C	0.083144	7.218356	3.164985	C	2.868535	6.896503	-0.350684
C	-0.393550	-4.842733	-4.799656	C	0.024501	5.503518	4.875213	C	4.404715	5.245931	0.438610
H	0.213715	-3.841398	-3.003471	H	-0.118433	3.485446	4.163376	H	3.575771	3.305285	0.851781
H	-1.497778	-1.344503	-6.065750	H	-0.016147	6.537320	1.130901	H	0.818493	6.277293	-0.577621

H	-1.793062	-3.321416	-7.500465	H	0.139819	8.270726	2.904409	H	2.654740	7.908728	-0.688076
H	-0.081837	-5.820246	-4.442865	H	0.036346	5.233575	5.926378	H	5.408522	4.944174	0.730912
N	0.579693	-0.783711	3.630233	N	-0.118755	-3.816547	1.604133	N	1.092922	-3.621415	0.008608
H	0.236917	-1.179905	2.744324	H	-0.132651	-2.876248	2.013429	H	1.417894	-2.668110	-0.190069
N	-0.409626	-1.403024	-3.541261	N	-0.157356	3.824216	1.610235	N	1.095056	3.656996	0.225658
H	-0.001073	-1.654676	-2.634944	H	-0.208411	2.884678	2.018376	H	1.438895	2.721244	0.463986
C	0.849823	-4.627025	7.808430	F	0.196391	-7.792283	5.441068	C	5.317786	-7.506256	-0.223121
C	-1.178571	-5.907813	-6.919578	F	0.190592	7.789015	5.455631	H	5.443126	-7.867660	-1.250643
F	1.717390	-5.618981	7.496839	C	-0.259329	0.005043	2.586948	H	5.119461	-8.374885	0.411399
F	-0.340672	-5.237606	8.005539	O	-0.309449	1.113777	1.990744	H	6.269092	-7.062702	0.086352
F	1.237043	-4.142861	9.002084	O	-0.181576	-1.103491	1.993780	C	5.304264	7.553320	-0.092901
F	-2.328531	-6.554017	-6.607806	C	-0.256758	0.008291	4.107611	H	5.906009	7.376836	-0.992101
F	-0.195791	-6.822307	-6.781683	H	-0.677074	-0.919654	4.500715	H	5.975010	7.477375	0.768711
F	-1.260371	-5.616930	-8.232327	H	-0.806594	0.867966	4.496279	H	4.923202	8.577270	-0.143388
C	0.372956	-1.838202	0.128966	H	0.781491	0.084908	4.450186	C	1.861871	-0.023899	0.240697
O	0.729536	-1.362712	-0.982886					O	1.288164	0.917600	0.854553
O	-0.259162	-1.185830	1.008519					O	1.262806	-0.867478	-0.484471
C	0.734236	-3.278905	0.456986					C	3.365535	-0.185207	0.406221
H	1.456527	-3.282715	1.281510					H	3.548072	-0.995948	1.121522
H	1.169574	-3.789734	-0.403521					H	3.825448	0.727586	0.788961
H	-0.154761	-3.816263	0.799503					H	3.825991	-0.471799	-0.542546

Anion complex **2**@$PhCO_2^-$ ΔG^0 = -2496.78196 au				Anion complex **3**@$PhCO_2^-$ ΔG^0 = -2644.42200 au				Anion complex **4**@$PhCO_2^-$ ΔG^0 = -3053.29626 au			
C	-0.226776	0.029890	-5.189450	C	-0.456410	0.057753	-5.004475	C	-5.291164	0.058997	0.084948
N	-0.679385	0.021555	-2.338859	N	-0.113963	0.089955	-2.135325	N	-2.404112	0.089805	-0.052082
C	-0.324047	-1.143504	-4.440784	C	-0.448345	-1.100591	-4.228045	C	-4.516020	-1.099048	0.140349
C	-0.365143	1.198668	-4.438572	C	-0.276569	1.231961	-4.275178	C	-4.550050	1.232266	-0.048698
C	-0.570278	1.152027	-3.038088	C	-0.120983	1.208417	-2.866583	C	-3.134332	1.207502	-0.103808
C	-0.564074	-1.103086	-3.046292	C	-0.266541	-1.044866	-2.823759	C	-3.102718	-1.043143	0.057981
S	-0.177854	-2.786913	-5.005727	S	-0.612589	-2.741319	-4.786101	S	-5.081976	-2.741518	0.273285
S	-0.319450	2.843502	-5.012160	S	-0.186350	2.855141	-4.899999	S	-5.164838	2.855864	-0.195340
C	-0.465783	-3.407214	-3.390237	C	-0.421578	-3.350911	-3.157677	C	-3.443853	-3.348631	0.203889
H	-0.474583	-4.475077	-3.232656	H	-0.443551	-4.416931	-2.995926	H	-3.282240	-4.414267	0.246387
C	-0.666250	-2.431048	-2.466049	C	-0.246851	-2.373086	-2.228080	C	-2.507342	-2.369733	0.090210
C	-0.550821	3.456379	-3.385375	C	0.043918	3.498620	-3.289618	C	-3.542644	3.496569	-0.315426
H	-0.587560	4.523493	-3.231657	H	0.161768	4.563749	-3.166469	H	-3.409766	4.561107	-0.430501
C	-0.656468	2.477566	-2.446561	C	0.050036	2.546092	-2.319174	C	-2.576699	2.542962	-0.243773
C	0.065989	0.037176	-6.662326	C	-0.571072	0.036653	-6.501900	C	-6.792654	0.038744	0.087738
H	-0.434642	0.892721	-7.133214	H	-1.060865	0.955562	-6.847308	H	-7.173451	0.960477	0.544868
H	-0.345981	-0.868616	-7.124476	H	-1.208858	-0.800576	-6.812066	H	-7.150568	-0.795050	0.704326
C	1.576351	0.110669	-6.942201	C	0.806085	-0.092811	-7.174951	C	-7.359544	-0.097895	-1.336008
H	2.072673	-0.736456	-6.452764	H	1.290881	-1.012599	-6.825878	H	-6.972451	-1.018643	-1.789043
H	1.981483	1.023582	-6.488830	H	1.440787	0.743123	-6.856411	H	-6.996553	0.736527	-1.948298
C	1.868114	0.095032	-8.440315	C	0.685026	-0.108303	-8.696352	C	-8.885502	-0.118489	-1.329705
H	2.943545	0.156623	-8.633042	H	1.667744	-0.199850	-9.168716	H	-9.281624	-0.216261	-2.345009
H	1.384269	0.941905	-8.939634	H	0.218745	0.814941	-9.057936	H	-9.284940	0.805171	-0.896561
H	1.492905	-0.826576	-8.899005	H	0.068300	-0.950673	-9.028813	H	-9.260271	-0.959744	-0.736180
N	-0.964762	-2.525712	-1.114601	N	-0.044165	-2.485893	-0.857704	N	-1.124167	-2.479967	-0.011766
H	-1.241596	-1.658608	-0.644050	H	0.039978	-1.615014	-0.323699	H	-0.590585	-1.605294	-0.052521
N	-0.816961	2.571721	-1.070392	N	0.187653	2.691520	-0.943869	N	-1.193201	2.687011	-0.275880
H	-0.707476	1.709975	-0.522865	H	-0.055943	1.885117	-0.356483	H	-0.629083	1.883664	0.028924
C	-0.951155	-3.692648	-0.378730	C	0.155125	-3.681676	-0.203307	C	-0.457758	-3.669805	-0.154052
C	-0.941911	3.759604	-0.378119	C	0.492065	3.879947	-0.319362	C	-0.550269	3.869506	-0.528752
O	-0.536511	-4.761490	-0.842548	O	0.090287	-4.769223	-0.777626	O	-1.023346	-4.760418	-0.138824
O	-1.034187	4.851924	-0.952777	O	0.756203	4.909941	-0.940618	O	-1.133577	4.898885	-0.860143
C	-1.191020	-4.548863	1.908275	C	0.688613	-4.525397	2.074644	C	1.865622	-4.479457	-0.495006
H	-1.926596	-4.385527	2.703171	C	1.206116	-6.388122	4.116781	C	3.948342	-6.287802	-0.825239
H	-1.371296	-5.543408	1.488073	C	0.375247	-5.879536	1.888585	C	1.613454	-5.864252	-0.441012
C	0.222927	-4.460258	2.481475	C	1.261870	-4.115931	3.290225	C	3.183468	-4.026954	-0.723870
H	0.412168	-3.426053	2.799527	C	1.512021	-5.038058	4.300812	C	4.223437	-4.921072	-0.885388
H	0.947775	-4.691550	1.688799	C	0.641079	-6.794234	2.908736	C	2.657715	-6.762166	-0.607567
C	0.427249	-5.398926	3.667828	H	-0.071285	-6.210269	0.960829	H	0.611399	-6.228673	-0.268705
H	-0.312423	-5.157490	4.443900	H	1.512733	-3.066810	3.432877	H	3.378524	-2.959176	-0.778998
H	0.229077	-6.433511	3.356610	H	1.953855	-4.697777	5.233242	H	5.233488	-4.569287	-1.060225
C	1.834434	-5.292761	4.252795	H	0.393499	-7.840490	2.750436	H	2.469751	-7.828933	-0.564036
H	2.032704	-4.270061	4.595754	H	1.404217	-7.110370	4.902990	C	1.774653	4.747176	-0.581479
H	2.589180	-5.545501	3.499239	C	0.720985	4.787939	1.988688	C	3.834435	6.577111	-0.930161
H	1.968034	-5.967346	5.104669	C	1.177293	6.674720	4.024762	C	3.106448	4.400549	-0.266712
C	-0.985786	4.772512	1.849685	C	0.431269	4.481358	3.329222	C	1.497540	6.034571	-1.080804
H	-1.413708	4.456991	2.806592	C	1.248859	6.048763	1.675198	C	2.530674	6.944380	-1.251968
H	-1.652996	5.527332	1.421957	C	1.467399	6.976599	2.695429	C	4.134702	5.306315	-0.437660
C	0.405255	5.367472	2.067027	C	0.658593	5.415033	4.333788	H	3.321017	3.406180	0.115704
H	1.063470	4.595297	2.488208	H	0.027100	3.501433	3.574199	H	0.484616	6.315085	-1.329490

H	0.825706	5.653165	1.093621	H	1.483099	6.299129	0.650052	H	2.323481	7.936720	-1.635807
C	0.374938	6.581649	2.993421	H	1.875612	7.950111	2.437136	H	5.155680	5.037452	-0.193406
H	-0.056595	6.288763	3.959876	H	0.426651	5.155265	5.362897	N	0.901076	-3.498684	-0.330491
H	-0.294597	7.342118	2.569994	H	1.353072	7.405801	4.808048	H	1.232151	-2.525310	-0.306450
C	1.763022	7.181762	3.210412	N	0.434935	-3.520282	1.128433	N	0.820045	3.764923	-0.379005
H	2.439814	6.444350	3.656962	H	0.487266	-2.551192	1.460368	H	1.155565	2.871532	-0.005968
H	1.725490	8.051342	3.874390	N	0.477526	3.776653	1.048319	N	4.907632	7.536851	-1.111823
H	2.200064	7.502623	2.257919	H	0.133969	2.887015	1.418743	O	6.048945	7.195747	-0.826860
N	-1.450728	-3.555480	0.877411	C	-0.559272	0.131356	1.865834	O	4.630316	8.649683	-1.541199
H	-1.690794	-2.608431	1.172779	O	-0.753754	1.256832	1.335097	N	5.034676	-7.235348	-0.993756
N	-0.987970	3.615221	0.970684	O	0.230532	-0.744575	1.419812	O	4.776538	-8.432100	-0.966016
H	-0.750475	2.700563	1.354719	C	-1.335065	-0.179424	3.131905	O	6.167584	-6.799541	-1.156648
C	-0.957891	-0.138210	1.594824	C	-2.757546	-0.727174	5.482521	C	1.486555	0.123745	0.797855
O	-0.255284	0.833190	1.208976	C	-1.375261	-1.477516	3.648414	O	0.961420	1.265117	0.884734
O	-1.898952	-0.664075	0.937663	C	-2.021085	0.841803	3.797642	O	1.168848	-0.747297	-0.057230
C	-0.637914	-0.755956	2.944426	C	-2.723766	0.571695	4.971246	C	2.576586	-0.210714	1.794947
C	-0.014685	-2.003002	5.373163	C	-2.085643	-1.753544	4.815949	C	4.599618	-0.806987	3.636326
C	-1.584181	-1.556776	3.592290	H	-0.856724	-2.277912	3.130973	C	2.921821	-1.538883	2.059791
C	0.618848	-0.571088	3.529937	H	-1.995950	1.847486	3.388817	C	3.244357	0.817635	2.467750
C	0.933190	-1.200672	4.734213	H	-3.246516	1.372972	5.485951	C	4.258658	0.522190	3.377147
C	-1.278860	-2.172324	4.804910	H	-2.114130	-2.767907	5.204584	C	3.924671	-1.838316	2.980744
H	-2.562082	-1.697797	3.138886	H	-3.307379	-0.939611	6.395225	H	2.391018	-2.341904	1.557833
H	1.350305	0.054114	3.025870					H	2.965135	1.848001	2.267748
H	1.918485	-1.069615	5.172961					H	4.780989	1.327152	3.886380
H	-2.021083	-2.793332	5.299441					H	4.178210	-2.874565	3.186428
H	0.232107	-2.497515	6.309025					H	5.386438	-1.037847	4.349055

Anion complex **5**@$PhCO_2^-$ ΔG^0 = -3318.30710 au				Anion complex **6**@$PhCO_2^-$ ΔG^0 = -2842.85217 au				Anion complex **7**@$PhCO_2^-$ ΔG^0 = -2722.96742 au			
C	-5.437059	0.052398	0.011546	C	-0.029354	0.031410	5.146066	C	-0.084082	-0.008666	5.607436
N	-2.548046	0.077894	-0.077443	N	-0.126533	0.080752	2.261322	N	-0.100936	0.001161	2.719269
C	-4.665982	-1.108310	0.059624	C	0.054950	1.210378	4.404545	C	-0.087432	1.164442	4.852644
C	-4.690782	1.225856	-0.090426	C	-0.164384	-1.121205	4.371371	C	-0.086482	-1.176462	4.844283
C	-3.274335	1.198828	-0.123737	C	-0.191512	-1.055588	2.957332	C	-0.107535	-1.129633	3.428775
C	-3.251182	-1.055073	0.002953	C	-0.012230	1.192592	2.990141	C	-0.084202	1.126959	3.436741
S	-5.238761	-2.751174	0.156836	S	0.252199	2.834489	5.005976	S	-0.098073	2.804492	5.442135
S	-5.300851	2.852736	-0.218016	S	-0.300526	-2.767514	4.928290	S	-0.064909	-2.820536	5.422733
C	-3.600894	-3.361478	0.104271	C	0.219832	3.480336	3.378292	C	-0.076369	3.429762	3.807370
H	-3.442224	-4.427996	0.131221	H	0.315429	4.545849	3.238822	H	-0.072412	4.498258	3.659661
C	-2.659211	-2.384011	0.025128	C	0.067871	2.527391	2.420574	C	-0.064932	2.459741	2.854723
C	-3.675597	3.491537	-0.302931	C	-0.368686	-3.358100	3.280250	C	-0.109253	-3.434763	3.784292
H	-3.539374	4.557437	-0.398864	H	-0.453836	-4.420179	3.111475	H	-0.113907	-4.502262	3.629519
C	-2.711495	2.535569	-0.234036	C	-0.293281	-2.371477	2.348136	C	-0.136363	-2.458627	2.838309
C	-6.938442	0.037604	-0.008186	C	0.088475	-0.001109	6.642838	C	-0.019959	-0.014332	7.107697
H	-7.322462	0.948374	0.468144	H	-0.497860	-0.838124	7.041822	H	-0.544172	-0.896322	7.496254
H	-7.307952	-0.810901	0.580804	H	-0.335736	0.917503	7.067069	H	-0.541456	0.866289	7.502880
C	-7.486589	-0.059377	-1.442381	C	1.552752	-0.142973	7.092347	C	1.431820	-0.017492	7.616400
H	-7.098433	-0.970738	-1.913235	H	2.135367	0.692125	6.684773	H	1.951295	0.864482	7.222430
H	-7.111037	0.788121	-2.028644	H	1.971209	-1.062447	6.665204	H	1.950285	-0.897954	7.217881
C	-9.012669	-0.071354	-1.456353	C	1.671027	-0.170860	8.613669	C	1.488477	-0.021679	9.141509
H	-9.396306	-0.143506	-2.478572	H	2.714730	-0.276876	8.924872	H	2.523325	-0.029367	9.496885
H	-9.412461	0.844467	-1.007070	H	1.104654	-1.010120	9.032362	H	0.983424	-0.905648	9.546438
H	-9.399543	-0.923804	-0.887043	H	1.278290	0.753516	9.051443	H	0.994733	0.866432	9.551224
N	-1.275323	-2.495389	-0.050959	N	-0.031995	2.656589	1.041323	N	-0.026025	2.574652	1.470788
H	-0.740590	-1.621094	-0.069212	H	-0.292818	1.815876	0.510487	H	0.105336	1.710271	0.932450
N	-1.328112	2.679657	-0.247472	N	-0.275915	-2.452556	0.960076	N	-0.201431	-2.565282	1.454260
H	-0.767289	1.874047	0.055286	H	-0.087973	-1.581423	0.451086	H	-0.365682	-1.700814	0.924625
C	-0.603683	-3.688412	-0.164058	C	0.104909	3.844806	0.362291	C	-0.091084	3.771855	0.793034
C	-0.681618	3.866586	-0.487109	C	-0.291530	-3.634185	0.255427	C	-0.136242	-3.757051	0.767528
O	-1.176712	-4.777322	-0.159513	O	0.429560	4.895178	0.919137	O	-0.265228	4.847652	1.368661
O	-1.270851	4.897143	-0.810491	O	-0.473312	-4.726654	0.793859	O	0.072645	-4.833796	1.329630
C	1.726502	-4.517263	-0.413415	C	-0.013634	4.696326	-1.975495	C	-0.067995	4.629858	-1.541111
C	3.824072	-6.354818	-0.658183	C	0.209550	6.476428	-4.090393	C	-0.286193	6.496005	-3.662749
C	1.476937	-5.893555	-0.286088	C	0.343747	6.031245	-1.737367	C	-0.025559	6.005338	-1.281299
C	3.040386	-4.079565	-0.666324	C	-0.255968	4.277659	-3.294822	C	-0.220832	4.198600	-2.867936
C	4.079645	-4.988032	-0.786846	C	-0.147899	5.164075	-4.359289	C	-0.323502	5.117079	-3.905432
C	2.526137	-6.798504	-0.413243	C	0.456722	6.923054	-2.805392	C	-0.140064	6.911412	-2.337748
H	0.476037	-6.248596	-0.086984	H	0.532721	6.372594	-0.729401	H	0.092419	6.364511	-0.268095
H	3.237614	-3.015328	-0.769861	H	-0.529660	3.242176	-3.484702	H	-0.265286	3.131891	-3.077950
H	5.086667	-4.630816	-0.980854	H	-0.334002	4.843998	-5.379668	H	-0.443215	4.752900	-4.923969
H	2.322868	-7.860639	-0.311397	H	0.733835	7.958829	-2.635222	H	-0.109929	7.976036	-2.114719
C	1.638837	4.764605	-0.507744	C	0.020203	-4.518292	-2.018911	C	-0.209556	-4.606184	-1.567917
C	3.703701	6.633823	-0.788145	C	0.260734	-6.489492	-3.942756	C	-0.035574	-6.468950	-3.695901
C	2.961489	4.432230	-0.163647	C	0.977507	-4.405256	-3.034240	C	-0.087342	-4.172643	-2.897081
C	1.362845	6.051142	-1.001829	C	-0.817386	-5.639817	-1.982562	C	-0.249612	-5.981652	-1.309315
C	2.396316	6.971427	-1.136684	C	-0.690574	-6.638969	-2.946332	C	-0.158286	-6.886358	-2.369231
C	3.985915	5.356466	-0.303178	C	1.097962	-5.388349	-4.013144	C	-0.009442	-5.089547	-3.937990
H	3.178885	3.437855	0.218419	H	1.640395	-3.544815	-3.049989	H	-0.055095	-3.105540	-3.107036
H	0.353685	6.324939	-1.273612	H	-1.566863	-5.729824	-1.205262	H	-0.352857	-6.341948	-0.294782

H	2.173769	7.964093	-1.519161	H	-1.329522	-7.516239	-2.933188	H	-0.192883	-7.951245	-2.148208
H	4.999460	5.079753	-0.030375	H	1.834739	-5.309493	-4.806173	H	0.078843	-4.723528	-4.959132
N	0.756233	-3.524422	-0.286624	N	-0.153317	3.721093	-0.977828	N	0.053126	3.629629	-0.560952
H	1.090766	-2.553572	-0.272285	H	-0.462195	2.795007	-1.289509	H	0.198354	2.673630	-0.900788
N	0.683202	3.764507	-0.338739	N	-0.093590	-3.463641	-1.091082	N	-0.312708	-3.609058	-0.582018
H	1.024024	2.867245	0.017293	H	0.198040	-2.529407	-1.397396	H	-0.496988	-2.657294	-0.912402
C	4.929617	-7.343127	-0.844238	F	0.320588	7.350776	-5.124890	C	-0.373100	7.488889	-4.794889
C	4.791207	7.639965	-0.984991	F	0.378417	-7.460657	-4.884583	H	0.609941	7.648224	-5.253389
F	6.129039	-6.852878	-0.473993	C	-0.393585	0.112237	-1.732082	H	-0.734680	8.458762	-4.441439
F	5.068555	-7.727458	-2.136808	O	0.438793	-0.687880	-1.224996	H	-1.047418	7.134052	-5.580243
F	4.735174	-8.476240	-0.141374	O	-0.991979	1.025400	-1.100586	C	0.086555	-7.460336	-4.826028
F	5.127663	7.789016	-2.289012	C	-0.703684	-0.033294	-3.208158	H	1.133643	-7.588830	-5.124660
F	5.926214	7.316991	-0.338476	C	-1.278350	-0.317480	-5.932987	H	-0.466428	-7.124173	-5.708562
F	4.434958	8.870941	-0.558417	C	-1.775290	0.660975	-3.778815	H	-0.297358	-8.441225	-4.531271
C	1.353088	0.111063	0.836512	C	0.079797	-0.865863	-4.012489	C	-0.196821	0.004062	-1.314644
O	0.838134	1.257797	0.914022	C	-0.202973	-1.007479	-5.370201	O	-0.924088	-0.831825	-0.712995
O	1.022467	-0.768768	-0.003712	C	-2.064553	0.516972	-5.135095	O	0.550070	0.849724	-0.751559
C	2.448825	-0.222732	1.828513	H	-2.380523	1.308889	-3.151427	C	-0.221770	-0.008851	-2.829782
C	4.483648	-0.820770	3.656537	H	0.918482	-1.391345	-3.566841	C	-0.269632	-0.031739	-5.627796
C	2.811180	-1.550407	2.073085	H	0.414411	-1.653711	-5.988137	C	0.756236	0.676070	-3.557528
C	3.106477	0.803890	2.513747	H	-2.902214	1.054348	-5.570974	C	-1.223826	-0.705578	-3.512388
C	4.125911	0.507722	3.417335	H	-1.502655	-0.428177	-6.990295	C	-1.251861	-0.713421	-4.906327
C	3.820158	-1.850767	2.987011					C	0.736834	0.661169	-4.951547
H	2.290205	-2.351788	1.558254					H	1.531050	1.218106	-3.022756
H	2.815104	1.833584	2.327968					H	-1.978967	-1.240165	-2.942955
H	4.639559	1.311594	3.937075					H	-2.036907	-1.252000	-5.429839
H	4.087808	-2.886675	3.176195					H	1.503769	1.190830	-5.509914
H	5.274884	-1.052241	4.364214					H	-0.288239	-0.040447	-6.714193

7. References

[1] APEX2,. Bruker AXS Inc., Madison, Wisconsin, USA, **2013**.

[2] SADABS,. Bruker AXS Inc., Madison, Wisconsin, USA, **2012**.

[3] Sheldrick G. M., *Acta Crystallogr.* **1990**, A46, 467-473; Sheldrick, G. M. *Acta Cryst*., **2008**, A64, 112–122.

[4] *International Tables for Crystallography*, Ed. A. J. C. Wilson, Kluwer: Dordrecht, **1992**, Vol.C.

[5] Rodríguez-Barrientos, D.; Rojas-Hernández, A.; Gutiérrez, A.; Moya-Hernández, R.; Gómez-Balderas, R.; Ramírez-Silva, M. T., *Talanta*, **2009**, *80*, 754-762.
